# EasyGrid: a versatile platform for automated cryo-EM sample preparation and quality control

**DOI:** 10.1038/s41592-026-03127-5

**Published:** 2026-06-23

**Authors:** Olivier Gemin, Victor Armijo, Léa Lecomte, Michael Hons, Thibault Deckers, Caroline Bissardon, Christopher Rossi, Kévin Lauzier, Robert Janocha, Franck Felisaz, Jérémy Sinoir, Romain Linares, Anastasiia Babenko, Kirill Kovalev, Irina Prokhorova, Iskander Khusainov, Claudia Schreiner, Olga Kolesnikova, Veijo T. Salo, Sarah Schneider, Matthew W. Bowler, Georg Wolff, Wojciech P. Galej, Julia Mahamid, Christoph W. Müller, Kristina Djinovic Carugo, Sebastian Eustermann, Simone Mattei, Florent Cipriani, Gergely Papp

**Affiliations:** 1 Molecular Systems Biology Unit, European Molecular Biology Laboratory, Heidelberg, Germany; 2 European Molecular Biology Laboratory, EMBL Grenoble, Grenoble, France; 3 Centre for Structural Systems Biology (CSSB), DESY, Hamburg, Germany; 4 Collaboration for joint PhD degree between EMBL and Heidelberg University, Faculty of Biosciences, Heidelberg, Germany; 5 European Molecular Biology Laboratory, EMBL Hamburg, Hamburg, Germany; 6 Department of Structural and Computational Biology, Center for Molecular Biology, University of Vienna, Vienna, Austria; 7 Max Perutz Laboratories, Vienna Biocenter Campus, Vienna, Austria; 8 EMBL Imaging Centre, European Molecular Biology Laboratory, Heidelberg, Germany; 9 Cell Biology and Biophysics Unit, European Molecular Biology Laboratory, Heidelberg, Germany; 10 The Comammox Research Platform, University of Vienna, Vienna, Austria

**Keywords:** Molecular biology, Cell biology

## Abstract

Optimized sample preparation is essential for imaging biological macromolecules in their native state using single-particle cryo-electron microscopy (cryo-EM) or in situ cryo-electron tomography (cryo-ET). Here we present EasyGrid, a modular, automated platform designed to streamline and standardize cryo-EM/ET sample preparation. EasyGrid integrates in-line plasma treatment of the sample support, microfluidic dispensing, blot-less sample spreading, jet-based vitrification and grid quality control via light interferometry. We demonstrate its effectiveness by preparing grids for multiple purified macromolecular complexes and resolving their structures with cryo-EM. Additionally, EasyGrid achieves improved vitrification of large mammalian cells compared to conventional plunge-freezing. By enabling systematic and high-throughput optimization, EasyGrid provides a robust and time-saving solution for both structural and cellular cryo-EM applications.

## Abstract (web summary)

EasyGrid is a fully automated and modular platform for high-throughput sample vitrification for cryo-EM and cryo-ET. It integrates in-line plasma treatment of the sample support, microfluidic dispensing, blot-less sample spreading, jet-based vitrification and grid quality control via light interferometry.

## Main

Cryo-electron microscopy (cryo-EM) allows investigating the structure of macromolecular assemblies both in purified solutions using single-particle analysis^1^ (SPA) and inside cells using cryo-electron tomography^2–4^ (cryo-ET). Even with recent advances in cryo-EM/ET methods, the challenges associated with preparing high-quality vitreous samples remain a limiting factor.

Preparing an adequate sample for SPA—forming a thin film (<50 nm) of vitreous water containing intact macromolecules and spread on a meshed support (grid) compatible with transmission electron microscopy (TEM)—requires the previous optimization of numerous parameters of sample preparation. Among these parameters, the thickness of the vitrified sample determines the signal-to-noise ratio of TEM images. Sample thickness depends on the microfluidic behavior of the sample solution and on the wettability of the meshed support during grid preparation. Therefore, sample thickness is influenced not only by sample composition, viscosity and ionic strength but also by grid geometry, grid surface properties, sample handling and vitrification speed^5^. Despite of recent studies aiming to understand, model and better control sample thinning during the vitrification process and therefore the resulting vitrified ice thickness^6–8^, it is common practice to screen the parameter space of sample preparation until producing adequate grids. New tools were developed to facilitate grid optimization: some instruments rely on the long-standing plunge-freezing approach^9^, while others harness microfluidic dispensing and/or ethane-jetting technologies^10–14^. Even with these tools, sample preparation remains a major bottleneck for most cryo-EM projects, emphasizing the need to develop automated screening of grid preparation parameters. Besides, the latest sample preparation instruments preclude vitrifying cells grown on grids for cryo-ET experiments, either because they can only handle grids that are already clipped in a copper cartridge^11^, which is toxic to cells, or because they are not equipped to remove culture medium from the cell-seeded grid before vitrification^10,12–14^. Additionally, plunge-freezing often fails to fully vitrify thick cellular samples due to insufficient cooling rates inside cells resulting in crystalline ice formation, deteriorating preservation of cellular ultrastructure^15^. In contrast, actively jetting cryogen (liquid ethane) onto nonclipped grids after quickly removing liquids from cell-seeded grids would yield higher cooling rates allowing to vitrify bulkier samples^16,17^. In summary, a modular and automated framework entailing all steps of grid production and including ethane-jetting technology is required to streamline grid optimization.

Beyond sample preparation, grid quality control is a major bottleneck of sample optimization because it still requires expensive access to cryo-EM instrumentation. In more detail, the standard procedure for controlling the quality of SPA grids is a two-step imaging process. An overview map (atlas) of the grid is first acquired at ~×100 magnification (~120 nm per pixel) to identify grid regions with adequately low sample thickness. Grids covered with thick or crystalline ice are readily discarded to make time for valuable grids. Second, a ‘high-resolution’ quality control is performed by imaging a selection of grid squares at typically >×40,000 magnification (<0.5 nm per pixel) to assess particle concentration, distribution and homogeneity, as well as potential ice contamination. Notably, only the first of these steps (sample thickness mapping) is necessary to control the quality of cell-seeded cryo-ET grids. Therefore, an instrument capable of performing ice-thickness mapping at cryo-temperature right after grid production would considerably speed up sample optimization for all cryo-EM pipelines; however, cryo-light microscopes (cryo-LM) lack the axial resolution necessary to characterize sample thicknesses below 400 nm due to the fundamental diffraction limit of light. Yet, digital holographic microscopy (DHM) is a light-based interferometry method that can map sample thickness variations in the nm-to-µm range^18^. Adapting DHM to cryogenic temperatures would enable a cost-effective solution for assessing grid quality before cryo-EM beamtime.

To address the challenges associated with cryo-EM/ET sample preparation, we developed EasyGrid, a fully automated and modular platform for high-throughput sample vitrification based on ethane-jetting technology. EasyGrid enables precise control over vitrification parameters. We demonstrated the system’s suitability for optimizing SPA grids by obtaining high-resolution reconstructions of four distinct macromolecular complexes (apoferritin, yeast ribosomes, guanidinase and pentamers of KR2 bacterial rhodopsin) as well as a medium-resolution reconstruction of the INO80–nucleosome complex. In addition, we validated that EasyGrid achieves superior vitrification quality for large human cells compared to conventional plunge-freezing methods. We further introduce the EasyGrid Control module, which performs automated cryogenic grid quality assessment by generating ice-thickness maps of the inserted samples. We show that the ice-thickness measurements obtained with this module are consistent with those derived from electron tomography, offering a rapid and cost-effective alternative to conventional grid-screening workflows on cryo-electron microscopes. Thus, the EasyGrid platform provides new solutions to improve and accelerate cryo-EM studies both in vitro and in situ.

## Results

### EasyGrid modules automate sample preparation and quality control

EasyGrid is a standalone platform equipped with multiple modules that perform all key functions for cryo-EM/ET sample preparation, vitrification and short-term storage at liquid nitrogen (LN_2_) temperature (Supplementary Video 1 and Supplementary Note 1). EasyGrid includes the following modules:

#### Grid loading and handling modules

**Fig. 1:**
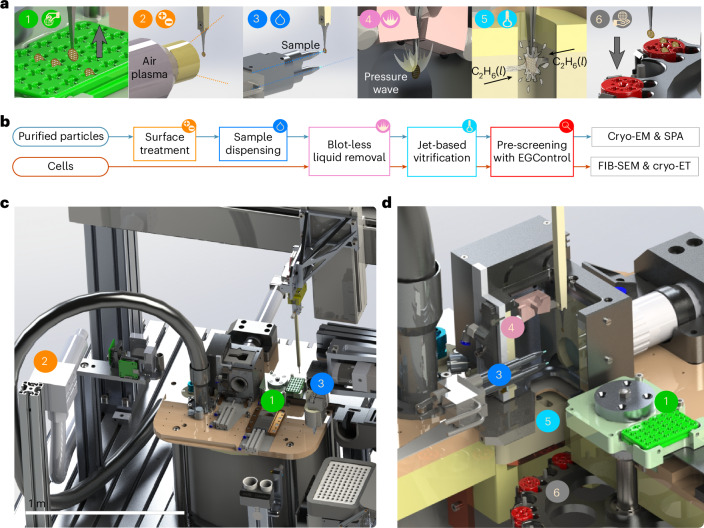
EasyGrid, a modular platform combining key technologies for cryo-EM grid preparation and quality control. **a**, Overview of the EasyGrid platform: schematics of EasyGrid modules. (1) Grid loading rack. (2) Plasma torch. (3) Two-nozzled microfluidic dispenser. (4) Pressure-wave generator. (5) Ethane-jetting slot. (6) Grid-storing carousel. **b**, Flow-chart of the two main workflows developed for EasyGrid, which aim at vitrifying purified protein solutions for cryo-EM and SPA or vitrifying cells for in situ imaging such as cryo-ET of lamellae milled with a FIB-SEM. **c**, Rendering of the whole EasyGrid machine. (1) Grid loading rack. (2) Plasma torch. (3) Two-nozzled microfluidic dispenser (in park position). **d**, Close-up cut view on the high-humidity chamber and inner parts of the dewar. (1) Grid loading rack. (3) Two-nozzled microfluidic dispenser (in dispense position within the high-humidity chamber). (4) pressure-wave generator. (5) Ethane-jetting slot. (6) Grid-storing carrousel. Live close-ups in Supplementary Video 1. Scale bar, 1 m.

Sample supports are first loaded in the instrument using a custom grid rack (Fig. 1a). Compatible supports entail 3-mm-diameter cryo-EM grids covered with a perforated support foil. Individual grids are picked up by the automated grid handling module composed of a metallic gripper motorized by a three-axis cartesian robot that drives the grid to the other modules. A system of two cameras set up perpendicularly to the gripper axis is used to calibrate gripper position and to monitor grid gripping before and during grid preparation. Automated grid handling reduces risks of grid bending and the deterioration of the support foil.

#### Plasma treatment module

EasyGrid is equipped with an atmospheric plasma treatment module (Fig. 1a) enabling the rapid and controlled enhancement of grid hydrophilicity. The water-cooled plasma torch is set up on a fixed holder, oriented perpendicularly to the grid and it outputs a power of 1 kVA at a frequency of 21 kHz. During plasma treatment, the grid is moved back and forth several times 12 mm in front of the torch to charge the surface of the grid without damaging the gripper. Five to ten such passages (~5–10-s total duration) are typically performed before delivering sample solutions on the grid.

#### Dual-nozzle picoliter drop dispenser

Sample solutions are provided by the user in a 96-well plate (Thermo Scientific, 0.2-ml non-skirted 96-well PCR Plate) inserted in a covered and thermoregulated dock (4–37 °C). Sample pipetting and deposition on the grid is performed automatically by the pipette robot (Fig. 1a) composed of two custom-designed, motorized and temperature-controlled (4–37 °C) piezoelectric picoliter (pl) drop dispenser heads. Once loaded with >15 µl of solution, the piezoelectric elements of the dispenser heads are first tuned to achieve monodisperse droplet dispensing using a stroboscopic observation module synchronized with drop-dispensing events. The dispenser heads can then deliver droplets of ~50 pl at a rate of up to 1,000 droplets per second at custom locations on the grid. A single charge of sample solution allows the preparation of at least 30 grids.

#### Humidity-controlled, pressure-wave-based sample-spreading module

Direct freezing of sample droplets dispensed on grids resulted in samples that were too thick for TEM imaging. To prepare electron-transparent, thin sample layers, EasyGrid is equipped with a pressure-wave generator composed of two nozzles aiming at each side of the grid from a grazing angle (Fig. 1a). These nozzles are mounted in a small temperature-regulated chamber (4–37 °C) separated from the vitrification module by a trapdoor. High humidity (typically >85%) is maintained in this chamber to limit sample evaporation. The nozzles conveying the pressure wave to the grid are powered with oil-free, filtered, compressed air and operated with an over-sampled digital output module (~1-µs switching time) ensuring the programmability and repeatability of pressure pulses. Sample spreading is performed by positioning the grid at the aiming point of the pressure-wave generator and releasing a brief blast of air to sweep both sides of the grid. Typical spreading durations are 30 ms for biochemical solutions and 300 ms to remove culture medium from a cell-seeded grid. After spreading and an optional, adjustable time delay, the grid is driven within 200 ms to the ethane-jetting vitrification module. A detailed assessment of the pressure-wave-based spreading system is available in the Supplementary Note 1.

#### Vitrification and storage module

The sample vitrification and storage module includes a dewar that is automatically topped-up with LN_2_, a temperature-regulated ethane reservoir with two output orifices facing each other (Fig. 1a), an ethane recycling pool and a grid storage carousel (Fig. 1a). Ethane is liquefied in the precooled reservoir by flowing ethane gas through it. A single charge of ethane lasts for a whole day of operation thanks to the ethane recycling device. Ethane jets are generated by pressurizing the ethane reservoir to push cryogen toward the jetting orifices. Once colliding jets are stable for ~500 ms, the grid is driven to the jets’ collision point to achieve sample vitrification. The grid is then kept in nitrogen vapor (−180 °C) to prevent thawing and to limit ice contamination. The grid is finally inserted in a slotted box of the storage module, composed of a carousel that can accommodate up to ten EasyGrid cryo-boxes. These custom boxes contain four grid slots and a cryo-compatible RFID chip (eCryoID tag) that facilitates sample tracking and grid inventory.

The modules described above can be programmed into custom workflows to prepare two types of samples: solutions of purified particles for SPA, or cells for in situ cryo-ET (Fig. 1b).

#### Grid pre-screening with the EasyGrid Control module

**Fig. 2:**
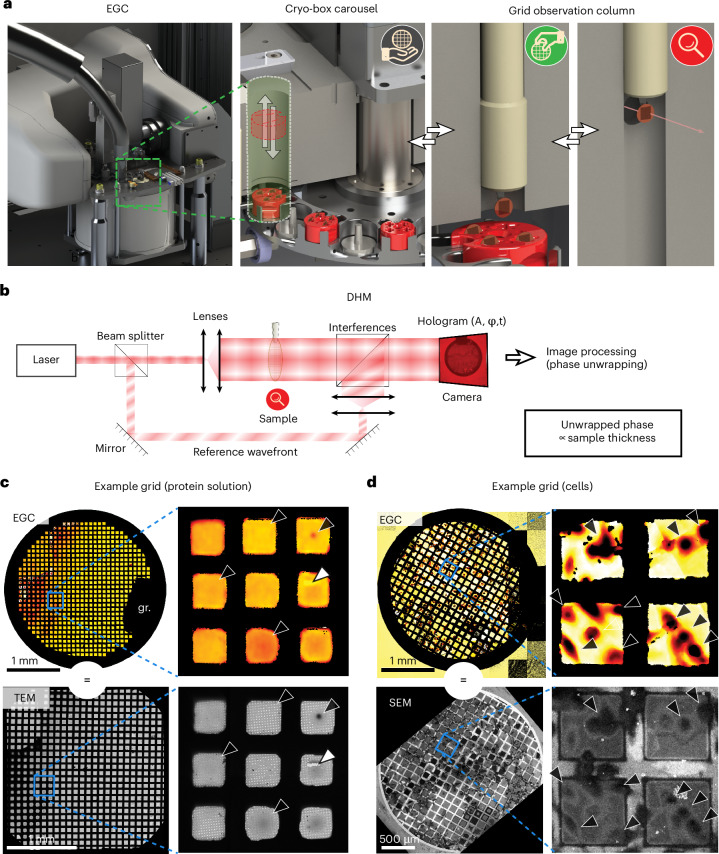
EasyGrid Control enables ice-thickness mapping at cryo-temperature to determine optimal grids for cryo-EM imaging. **a**, Schematics of the EGC module (first panel to the left) and close-ups on: the grid box exchange trapdoor (highlighted in green with white arrows representing box exchange by the user) and cryo-carousel (second panel), the grid pick-up point (third panel) and the grid observation point (fourth panel). **b**, Principle of DHM to acquire thickness maps with sub-wavelength axial resolution using interferometry. **c**, Atlases and close-ups of one grid prepared using the SPA process and imaged with both EGC (top) and cryo-EM (bottom). Insets show features visible in both modalities. White arrowhead: a hole in the carbon film. Black arrowheads, local maxima of ice thickness. More examples in Extended Data Fig. 1; gr, grid gripper. **d**, Atlas of one cell-seeded grid imaged with both EGC (top) and cryo-SEM (bottom). The insets show cells (arrowheads) visible in both modalities. More examples are in Extended Data Fig. 1. Source data

A primary criterion of grid usability for SPA is an appropriate thickness of the vitrified sample, which can be assessed with the EasyGrid Control (EGC) module. EGC is either integrated in the EasyGrid platform or built as a standalone ice-thickness mapping device. In this case, the motorized cryo-observation column is installed on top of a carousel similar to that of the EasyGrid main machine (Fig. 2a) with a capacity for 40 grids that are sequentially picked up by a motorized gripper (Fig. 2a). To maintain the gripper temperature stable below −170 °C, the column inner tube is made of copper, immersed in LN_2_ and thermally insulated from the exterior. Portholes of high optical quality installed across the observation point (Fig. 2a) enable mapping sample thickness with the interferometry setup depicted in Fig. 2b: a laser beam is split into an unmodulated ‘reference’ and a ‘measurement’ beam that illuminates the grid across the entire field of view of the charge-coupled device camera. The variations of refractive index and sample thickness across the grid modulate the wavefront of the measurement beam. The two beams are re-combined to interfere with each other, and the resulting hologram is recorded on a charge-coupled device camera. Unwrapping and analyzing the phase of the hologram allows deriving sub-wavelength measurements of optical thickness across the grid. Currently, EGC comprises three complementary imaging modes: thickness mapping at 2× magnification, screening at 10× magnification, and thickness mapping at 10× magnification.

##### Thickness mapping at ×2 magnification (~3 µm per pixel)

This observation mode allows rapidly (~5 min per grid) assessing overall sample quality and ice contamination.

##### Screening at ×10 magnification (~0.6 µm per pixel)

Because of the limited field of view at ×10 magnification (~0.35 × 0.35 mm), grids are imaged as a montage of 9 × 9 tiles (~15 min per grid).

##### Thickness mapping at ×10 magnification

**Extended Data Fig. 1:**
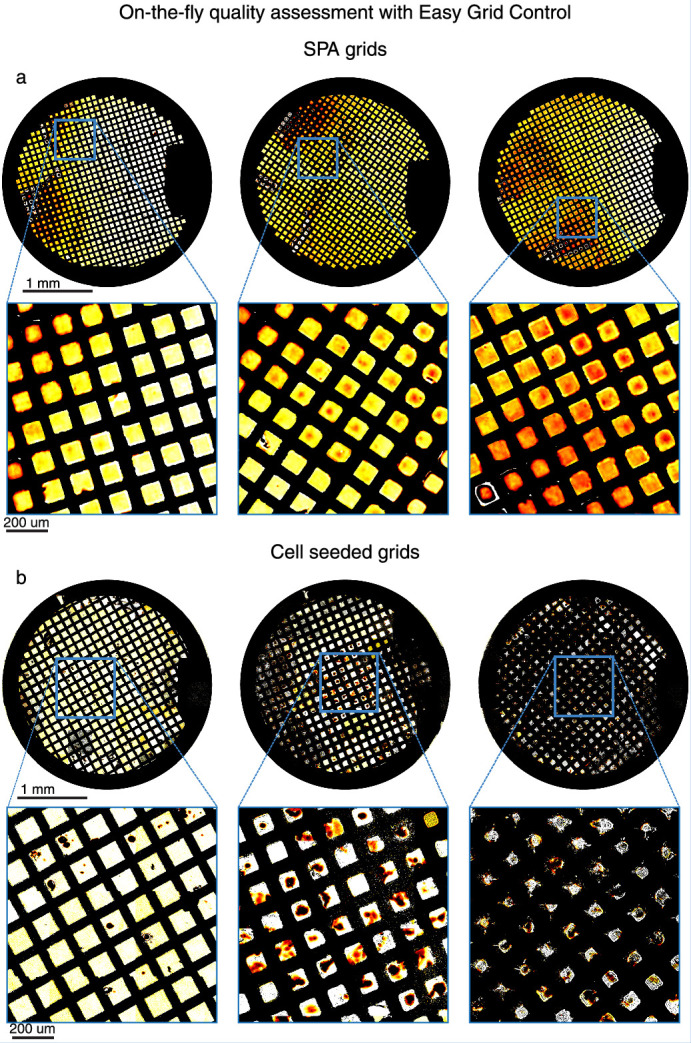
EasyGrid Control enables on-the−fly selection of optimal grids. **a**. EGC atlases of SPA grids. The gradient of thin ice in the 1^st^ inset suggests a potentially high-quality region for cryo-EM imaging; scale bars, 1 mm (top row) and 200 µm (bottom row). Colormap in arbitrary units (see Discussion). **b**. EGC atlases of cell-seeded grids. Thicker regions on the 2^nd^ and 3^rd^ atlases indicate that cells remained hydrated throughout sample processing; scale bars, 1 mm (top row) and 200 µm (bottom row). Source data

Deriving thickness measurements from a ×10 tile requires a thickness reference for each tile (for example ‘0 nm’ measured in a broken square) but such references are difficult to propagate across the montage because of measurement discontinuities (for example grid bars or thick squares). We addressed this issue by calibrating the average thickness value of each ×10 tile using a ×2 map of the same grid (Supplementary Note 1). Calibrated ×10 maps provide absolute ice-thickness measurements. They reliably correlate with cryo-EM atlases and describe the distribution of ice thickness at the level of individual squares for both SPA and cryo-ET samples (Fig. 2c,d). Thus, EGC allows rapid, inexpensive and automatic screening of batches of up to 40 grids to bring forward optimal specimens (Extended Data Fig. 1) for subsequent cryo-EM/ET imaging. A detailed performance assessment of the EGC module is available in Supplementary Note 1.

### EasyGrid provides high-quality samples for structural biology

#### EasyGrid automates cryo-EM grid preparation for single-particle analysis

**Fig. 3:**
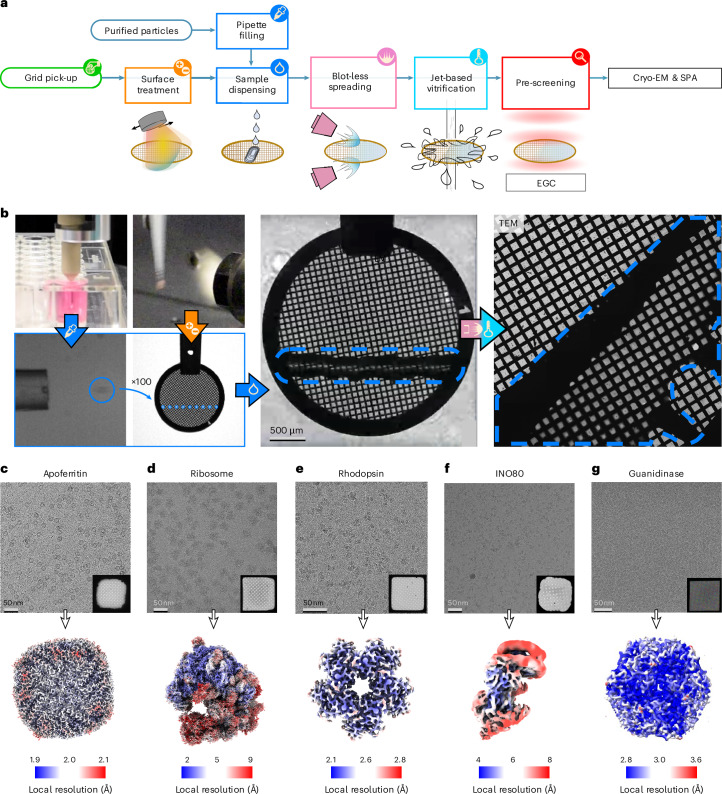
Automated grid preparation with EasyGrid enables high-quality cryo-EM acquisition and structure determination. **a**, Flow-chart of SPA grid preparation with EasyGrid which entails conditioning grids for optimal sample spreading before dispensing, spreading and jet-vitrifying a sample solution on the grid for subsequent cryo-EM imaging. Optional on-the-fly grid screening with EGC allows grid quality control before cryo-EM imaging. **b**, Illustrations of the SPA grid preparation process. Initial steps include (1) filling the pipette robot with the sample solution (top-left) then tuning the piezoelectric dispensing system to reproducibly dispense sample droplets (blue circle) and (2) treating the picked-up grid with plasma (second panel) and optional precoating steps (not shown). The pipette robot is then programmed to dispense droplets at desired locations (small blue circles), typically to form a line of ~10 nl of solution close to the midline of the grid (dashed blue line in the middle panel). After sample spreading and vitrification, the grid can be imaged in cryo-EM (right, the dashed blue line outlines the region where the dispensed sample was spread). Please note that the illustrations shown in this sequence do not correspond to the preparation steps of the same sample grid. The appearance of the resulting sample grid (right) may also vary substantially depending on the processing parameters and the nature of the sample. **c**–**g**. Top: example micrographs acquired on grids prepared with EasyGrid using apoferritin (**c**), yeast ribosomes (**d**), KR2 rhodopsin (**e**), INO80–0N80 (**f**) and guanidinase (**g**) solutions. Insets show grid squares in which the high-resolution micrographs were acquired. Bottom: cryo-EM density maps color-coded by local resolution, reconstructed after performing SPA on grids prepared with EasyGrid. Source data

**Extended Data Fig. 2:**
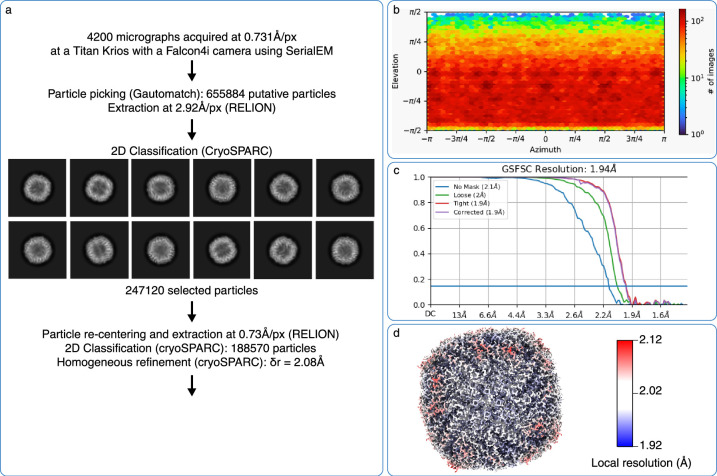
Processing scheme of the single−particle analysis of apoferritin prepared with EasyGrid. **a**. SPA processing workflow for the apoferritin data. 656k particles were picked from motion-corrected and dose^−^weighted micrographs using Gautomatch and extracted from micrographs binned 4 times (pixel size of 2.92 Å) using RELION. A subset of 247k particles were selected using 2D-classification in cryoSPARC, then re^−^extracted from unbinned micrographs and submitted to a second round of 2D-classification. The selected 189k particles underwent Homogenous Refinement in cryoSPARC. Re-centered particle stacks were extracted in RELION and underwent Bayesian polishing in RELION, then Homogenous Refinement in cryoSPARC, yielding a cryo-EM density map of apoferritin at 1.94 Å resolution (δr). **b**. Angular distribution calculated in cryoSPARC for particle projections. Heat map shows the number of particles for each viewing angle. **c**. Gold-standard Fourier shell correlation (GSFSC) plot for the final half-maps. The horizontal blue line indicates the resolution estimation threshold at 0.143. **d**. Final cryo-EM density map of apoferritin color-coded by local resolution computed in RELION^32^ and displayed in ChimeraX^38^.

**Extended Data Fig. 3:**
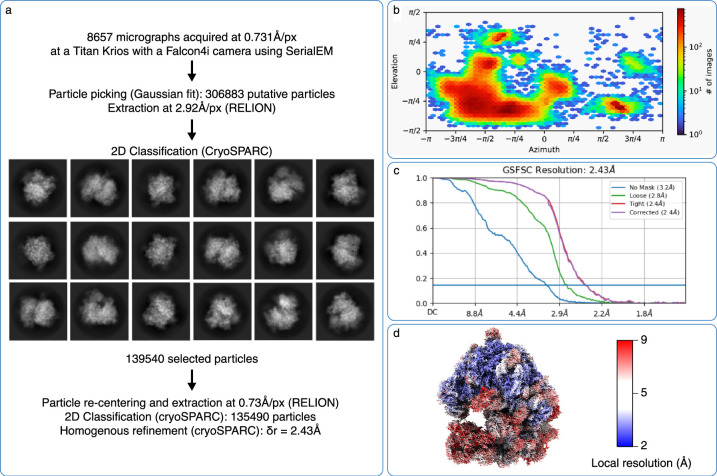
Processing scheme of the single-particle analysis of yeast ribosome prepared with EasyGrid. **a**. SPA processing workflow for the ribosome data. 307k particles were picked from motion-corrected and dose-weighted micrographs using python-based picking of signal densities measuring ~300 Å, then extracted from micrographs binned 4 times (pixel size of 2.92 Å) using RELION^32^. A subset of 140k particles were selected using 2D-classification in cryoSPARC^38^, then re-extracted from unbinned micrographs and submitted to a second round of 2D-classification. The selected 135k particles underwent Homogenous Refinement in cryoSPARC, yielding a cryo-EM density map of the full ribosome at 2.43 Å resolution (δr). **b**. Angular distribution calculated in cryoSPARC for particle projections. Heat map shows the number of particles for each viewing angle. **c**. Gold-standard Fourier shell correlation (GSFSC) plot for the final half-maps. The horizontal blue line indicates the resolution estimation threshold at 0.143. **d**. Final cryo-EM density map of the ribosome color-coded according to local resolution computed in RELION^32^ and displayed in ChimeraX. Because the small ribosomal subunit (SSU) can ratchet relative to the large ribosomal subunit^50^ (LSU), and particle alignment was driven by the LSU, the resolution of the SSU is comparatively lower (white/red domain at the base of the map).

**Extended Data Fig. 4:**
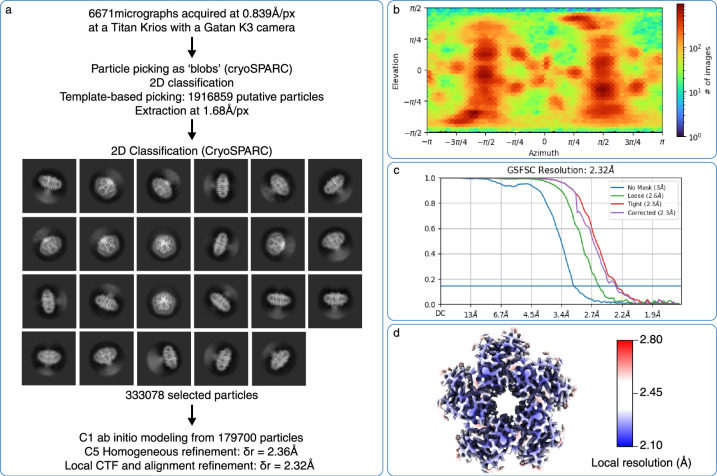
Processing scheme of the single-particle analysis of rhodopsin prepared with EasyGrid. **a**. SPA processing workflow for the KR2 rhodopsin data, performed exclusively with cryoSPARC. 1917k particles were picked from motion-corrected and dose-weighted micrographs, then extracted from micrographs binned 2 times (pixel size of 1.68 Å). A subset of 333k particles were selected using 2D-classification. 180k particles from those were used to generate a non-symmetrized (C1) *ab initio* model of the rhodopsin pentamer, which allowed structure refinement with C5 symmetry for the 333k particles. Subsequent local refinement yielded a cryo-EM density map of the KR2 rhodopsin pentamer at 2.32 Å resolution (δr). **b**. Angular distribution calculated in cryoSPARC for particle projections. Heat map shows the number of particles for each viewing angle. **c**. Gold-standard Fourier shell correlation (GSFSC) plot for the final half-maps. The horizontal blue line indicates the resolution estimation threshold at 0.143. **d**. Final cryo-EM density map of the rhodopsin complex color-coded by local resolution computed in cryoSPARC and displayed in ChimeraX.

**Extended Data Fig. 5:**
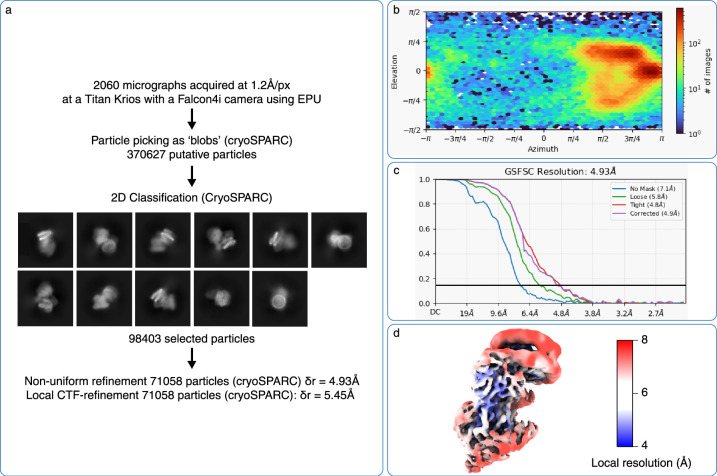
Processing scheme of the single-particle analysis of INO80–0N80 complex prepared with EasyGrid. **a**. SPA processing workflow for the INO80-0N80 data, performed exclusively with cryoSPARC. Patch motion correction was performed on the raw movies, followed by patch CTF estimation. After exposure curation, 1641 micrographs were selected for further processing. Blob particle picking and particle extraction with a 320×320 pixel box size were performed, followed by a round of 2D classification. Eleven relevant 2D classes exhibiting different particle orientations were selected corresponding to 98403 particles and used to generate two 3D *ab initio* models. One model, built using 71058 particles, was submitted to non-uniform refinement and resulted in a structure resolved at 4.93 Å (FSC threshold 0.143). Local CTF Refinement estimated the resolution at 5.45 Å (FSC threshold 0.143). **b**. Angular distribution calculated in cryoSPARC for particle projections. Heat map shows the number of particles for each viewing angle. **c**. Gold-standard Fourier shell correlation (GSFSC) plot for the final half-maps. The horizontal blue line indicates the resolution estimation threshold at 0.143. **d**. Final cryo-EM density map of the INO80–nucleosome complex color-coded by local resolution computed in cryoSPARC and displayed in ChimeraX.

**Extended Data Fig. 6:**
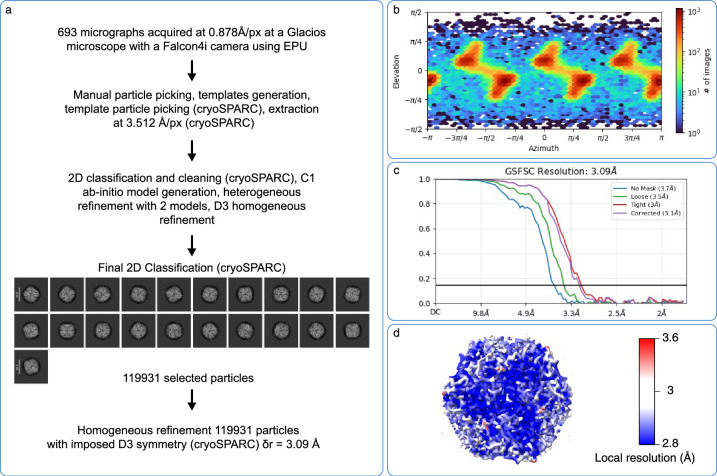
Processing scheme of the single-particle analysis of Guanidinase prepared with EasyGrid. **a**. SPA processing workflow for the Guanidinase data was performed exclusively with cryoSPARC. Patch motion correction was performed on the raw movies, followed by patch CTF estimation. After exposure curation, 502 micrographs were selected for further processing. Templates for template particle picking were generated by manual particle picking and 2D classification. 468313 particles picked using the templates were extracted at 3.512 Å/px with 56×56 pixel box size, cleaned by several rounds of 2D classification and re-extracted at 1.756 Å/px with 112×112 pixel box size. A set of 228937 particles was used for generation of two ab initio models without imposed symmetry. After heterogeneous refinement of the two classes followed by homogeneous refinement, 134644 particles corresponding to the good class were re-extracted at 0.878 Å/px, used for 2D classification. After final particle cleaning 119931 particles were subjected to homogeneous refinement with imposed D3 symmetry and resulted in a structure resolved at 3.09 Å (FSC threshold 0.143). **b**. Angular distribution calculated in cryoSPARC for particle projections. Heat map shows the number of particles for each viewing angle. **c**. Gold-standard Fourier shell correlation (GSFSC) plot for the final half-maps. The horizontal blue line indicates the resolution estimation threshold at 0.143. **d**. Final cryo-EM density map of the guanidinase color-coded by local resolution computed in cryoSPARC and displayed in ChimeraX.

**Extended Data Fig. 7:**
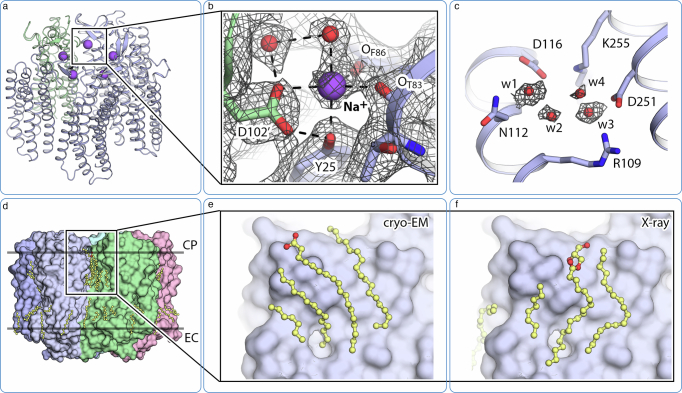
Cryo-EM reveals the high variability of lipid conformation on KR2 rhodopsin complexes. **a**. Overview of the KR2 pentamer with bound interprotomeric sodium ions shown as purple spheres. **b**. Detailed view of the interprotomeric sodium binding site. Cryo-EM densities were contoured at the level of 4 root mean square deviations (r.m.s.d.) and displayed as a black mesh. **c**. View of the active center of KR2. N112 is in the expanded conformation. The cryo-EM densities depicting the 4 water molecules in the internal cavity are shown as a black mesh and contoured at 4 r.m.s.d. **d**. Side view of the KR2 pentamer featuring lipidic molecules (yellow sticks and spheres) visible in the cryo-EM map. CP: cytoplasmic side; EC: extracellular side. **e**. Close-up on lipid fragments located on the cytoplasmic half of the interprotomeric cleft, as revealed by our cryo-EM study. **f**. Close-up on lipid fragments located on the cytoplasmic half of the interprotomeric cleft, as described in a previous study using X-ray crystallography (PDB ID: 6YC3).

The aim of SPA workflows is to derive the three-dimensional (3D) structure of molecular assemblies from thousands of cryo-EM images of macromolecular complexes immobilized in a thin vitrified ice layer. Preparing large areas of sample film that are suitable for high-resolution cryo-EM is challenging in practice: exploratory optimization is often required to simultaneously obtain a minimal thickness of the frozen sample layer, a dense particle distribution, varied particle orientations and the absence of ice crystals. To facilitate SPA sample optimization, we developed a versatile and reproducible workflow encompassing all steps of grid production (Fig. 3a). The EasyGrid SPA routine starts with enhancing the hydrophilicity of a grid using atmospheric plasma treatment, then dispensing the sample on the grid in a user-designed pattern (typically a line of 100 droplets) using microfluidic nozzles (Fig. 3b). A rapid sequence of sample spreading with the pressure-wave system followed by jet vitrification allows freezing intrinsically unstable thin sample films^19^ (Fig.3b) before storing them in cryo-boxes at LN_2_ temperature. In case a precoating solution (for example, Blue Dextran or a sacrificial protein layer) is used, it is dispensed and spread on the grid after the plasma treatment stage and then left to dry. This precoating step is subsequently followed by the standard sample preparation process, as described above. To demonstrate the applicability of our workflow we implemented SPA grid preparation protocols for different types of cryo-EM samples. These included established benchmarks such as human apoferritin and yeast ribosomes, but also less robust samples including the bacterial membrane protein KR2 rhodopsin, the bacterial enzyme guanidinase and the fungal multi-subunit ATP-dependent chromatin remodeler INO80 in complex with a nucleosome (Fig. 3c–g). Conventional cryo-EM acquisition and processing schemes yielded reconstructions at 1.9 Å, 2.4 Å, 2.3 Å, 3 Å and 5.9 Å, respectively. 3D refinements showed rich distributions of particle orientations (Extended Data Figs. 1–6). The KR2 rhodopsin complex structure was not previously determined by cryo-EM although a structure obtained at high salt concentrations with X-ray crystallography was already available^20^. Our cryo-EM map at 2.3 Å resolution shows key features of KR2 rhodopsin, including the interprotomeric sodium binding site and the functional, expanded conformation of the central region of the pentamer with numerous water molecules populating the internal cavities. The cryo-EM map additionally reveals all four of the water molecules located in the Schiff base cavity of KR2, whereas only three water molecules were resolved in X-ray crystallography data at a comparable map resolution (2.2 Å)^20^. Last, we observed a different organization of the aliphatic chains of lipid molecules in the interprotomeric clefts compared to published crystal structures, suggesting the high flexibility of lipid-protein interactions in KR2 complexes (Extended Data Fig. 7).

In summary, these data demonstrate that EasyGrid can prepare high-quality SPA grids, allowing the discovery of macromolecular structures through fully automated processes that decrease human error factors in grid preparation efforts.

#### EasyGrid improves cell vitrification quality for in situ cryo-ET

**Fig. 4:**
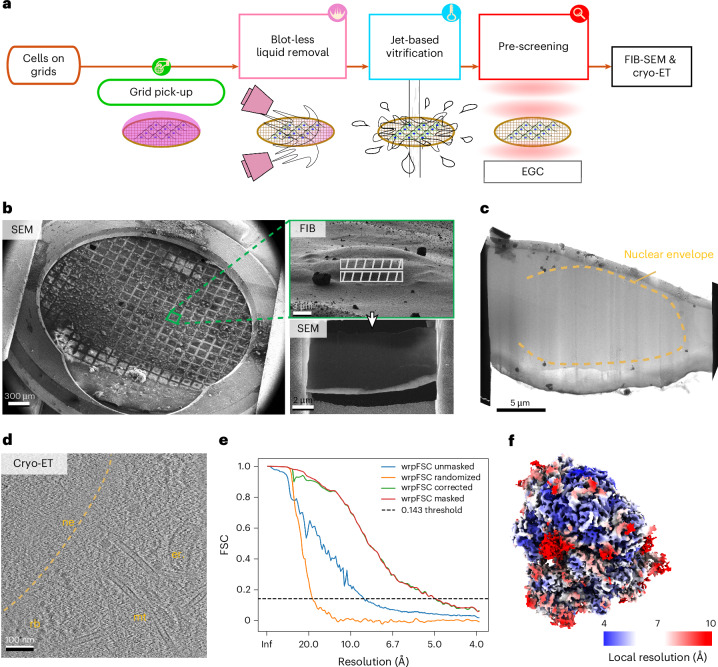
EasyGrid provides high-quality samples for in situ cryo-ET in large cells. **a**, Flow-chart of cell-seeded grid preparation from growing adherent cells on cryo-EM grids to sample thinning, vitrification and quality control, followed by FIB lamella preparation and cryo-ET. **b**, Example of lamella preparation in HeLa cells that were jet-vitrified on a cryo-EM grid and observed using SEM (scanning EM; left). The FIB image (top panel) is tilted 52° from the SEM optical path and shows the areas that were FIB-milled (gray patterns) to produce an electron-transparent lamella (lower panel) in the bulk of the cell. **c**, TEM overview of a lamella milled in the bulk of a HeLa cell prepared with EasyGrid. The absence of crystalline artifacts on the lamella illustrates good ice quality at a mesoscopic level. **d**. Central section of a cryo-tomographic reconstruction of a HeLa cell prepared with EasyGrid. er, endoplasmic reticulum; ne, nuclear envelope; mt, microtubule; rb, ribosomes. **e**, FSC curves for focused-refined maps of the ribosomal structure with the threshold of 0.143. FSC, Fourier shell correlation; wrpFSC, wrapped Fourier shell correlation; Inf, infinity. **f**, Cryo-EM density map at 6.8 Å resolution (δr) of a ribosome color-coded by local resolution, reconstructed after performing particle picking in cryo-ET volumes acquired on grids prepared with EasyGrid, followed by sub-tomogram averaging. Source data

**Extended Data Fig. 8:**
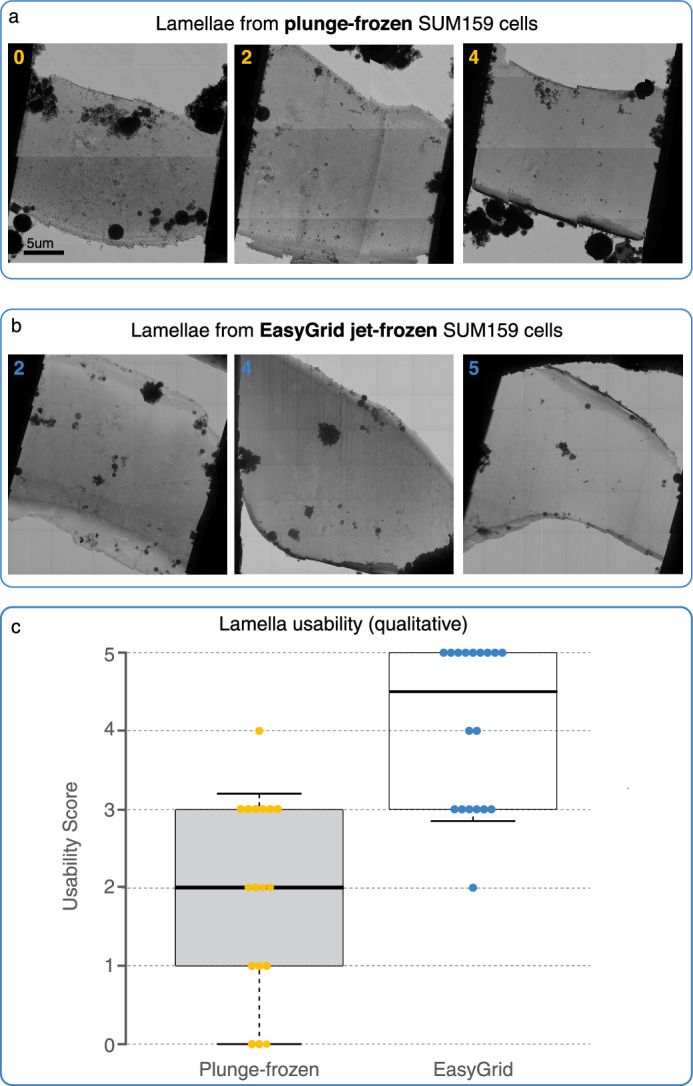
Qualitative assessment of ice quality in plunge- and jet-vitrified SUM159 cells. **a**. TEM images of lamellae from plunge-frozen SUM159 cells prepared with Leica GP2. A qualitative score (red number) was attributed to each lamella based on visual inspection. The score ranges from 0 describing a completely crystalline, unusable lamella to 5 describing a vitreous, high-quality lamella – independently of milling artifacts; scale bar, 5 µm. **b**. TEM images of lamellae from jet-vitrified SUM159 cells prepared with EasyGrid. **c**. Qualitative comparison of lamella usability in lamellae prepared by either plunge- or jet-vitrified SUM159 cells; n = 17 and 18 lamellae respectively. Source data

Sample vitrification without crystalline ice formation is required to preserve the integrity of nanometric, subcellular features for high-quality cryo-ET imaging, but plunge-freezing provides insufficient cooling rates to fully vitrify samples thicker than a few µm. Jet vitrification, as implemented in EasyGrid (Fig. 4a), was proposed to improve cooling rates^11,16,17^. To demonstrate the applicability of our cryo-ET sample preparation workflow, we cultured HeLa cells on micropatterned grids made of a gold mesh overlaid with perforated SiO_2_ support film, vitrified them with EasyGrid and then used a dual-beam focused ion beam/scanning electron microscope (FIB-SEM) to produce thin lamellae following established protocols^21–23^ (cryoFIB-milling; Fig. 4b). Cryo-EM overviews of the lamellae and the subsequent tilt-series (TS) acquisitions exhibited no crystalline ice artifacts in the central regions of cells, which often displayed crystalline artifacts in lamellae milled from plunge-frozen cells (Extended Data Fig. 8). The tomograms reconstructed from these cells did not exhibit crystalline artifacts (Fig. 4c) and displayed a preserved subcellular architecture (Fig. 4d).

**Fig. 5:**
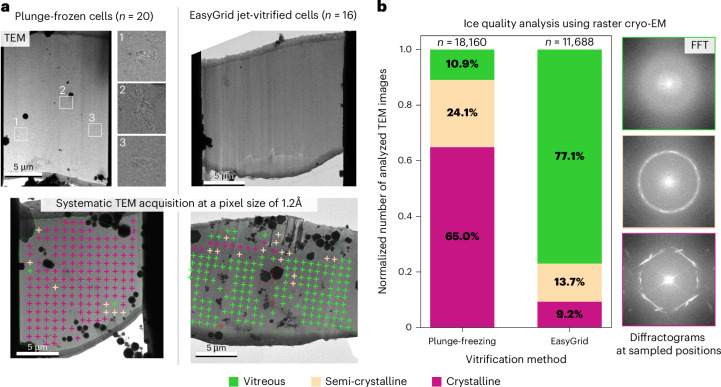
Jet vitrification improves the vitreous quality of ice in lamellae milled in large mammalian cells. **a**, Cryo-EM maps of lamellae milled in the bulk of either plunge-frozen or jet-vitrified HeLa cells (left and right columns, respectively). Insets in the upper left panel highlight crystalline ice artifacts that are already visible at ×2,250 magnification (5.6 nm per pixel). Crosses in the lower row represent positions where cryo-EM images were systematically acquired at ×105,000 magnification (0.12 nm per pixel) to quantify ice quality across lamellae. The color code added a posteriori is ‘unambiguously vitreous’, green; ‘semi-crystalline’, orange; ‘unambiguously crystalline’, magenta. **b**, Quantitation with EM diffraction analysis of ice crystallinity in lamellae prepared by either plunge-freezing or jet-freezing from Tokyo HeLa cells (*n* = 20 and 16 lamellae, respectively). Insets show 2D Fourier transforms of images acquired in three different positions and displaying either a faint water ring without peaks in vitreous regions (top inset); stronger signal in the water ring with possible peaks in semi-crystalline and/or thicker regions (middle inset); or strong peaks at the spatial frequencies of water in crystalline regions (bottom inset with a hexagonal ice diffraction pattern indicating low local cooling rate^15^). No *P* value was calculated for these categorical data. FFT, fast Fourier transform. Source data

**Extended Data Fig. 9:**
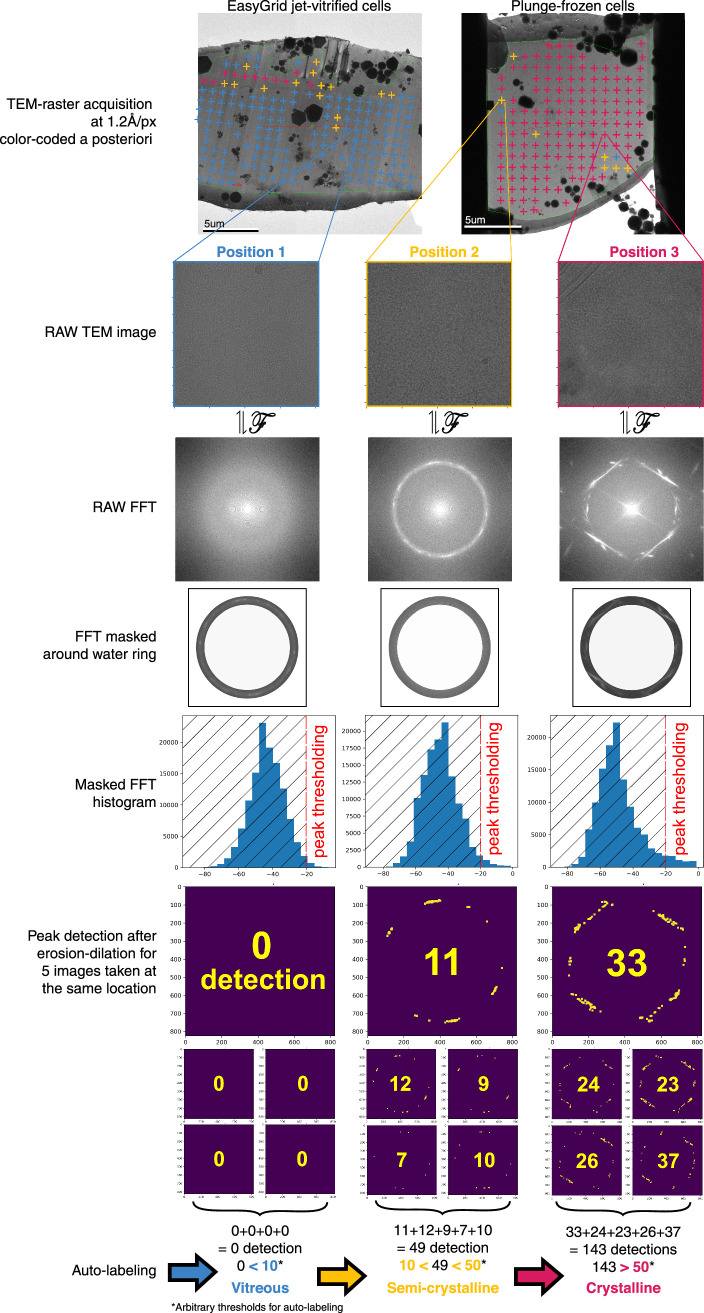
Ice quality quantitation analysis scheme. Step-by-step analysis workflow of ice crystallinity in lamellae using raster-TEM imaging, exemplified with images taken at three different positions and exhibiting either vitreous (left column), crystalline (right column) or ambiguous signal (middle column). At each location marked by a cross on the left-most panels, 5 TEM images were acquired at 1.2 Å pixel size to account for the observed variation of the 2D Fourier transforms upon repeated acquisition. In the Fourier power spectrum of those images, the water ring was masked and diffraction peaks were detected in this region by thresholding out low power values, eroding away single pixels to remove shot noise, dilating peaks back to their original size in the diffraction signal, and performing connected component analysis to count the number of diffraction peaks per image. The diffraction score of each sampled position was defined as the total number of peaks detected on all 5 images taken at this location. Empirical thresholds were arbitrarily defined to automatically label positions in three categories: ‘unambiguously vitreous’ (green color, score < 10), ‘unambiguously crystalline’ (magenta color, score > 50) and ‘ambiguous: thick and/or semi-crystalline’ (orange color, all other positions). All images were then visually inspected to correct false attributions, yielding the ice quality quantitation displayed in Fig.5; scale bars, 5 µm.

**Extended Data Fig. 10:**
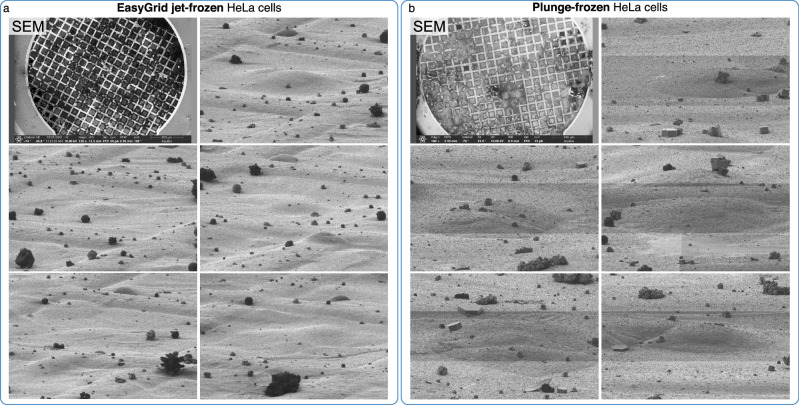
SEM images of jet-vitrified VS plunge-vitrified cells. **a**. Top Left: atlas of a HeLa Tokyo cell-seeded grid, vitrified by EasyGrid and imaged in an Aquilos FIB-SEM, prior to lamella milling. The rest of the images show individual cells as seen in the Aquilos FIB-SEM prior to milling out the lamellas. We notice the that the overall shape of the cells is similar to the ones vitrified using plunge-freezing method shown on b. **b**. Top Left: atlas of a HeLa Tokyo cell-seeded grid, vitrified in a Leica GP2 plunger and imaged in an Aquilos FIB-SEM, prior to lamella milling. The rest of the images show individual cells as seen in the Aquilos FIB-SEM prior to milling out the lamellas. Source data

To characterize the improvement of ice quality in cells prepared with EasyGrid compared to plunge-freezing, we first conducted a qualitative study on mammalian SUM159 cells (Extended Data Fig. 9) and performed a quantitative analysis of lamellae FIB-milled at mid-height of HeLa cells. Low magnification overviews of most lamellae milled in plunge-frozen cells displayed crystalline ice artifacts, whereas the majority of lamellae milled in jet-vitrified cells seemed vitreous (Fig. 5a and Extended Data Fig. 9). To quantify crystallinity, we carried out systematic cryo-EM raster imaging of the milled lamellae at a pixel size of 1.2 Å and analyzed the two-dimensional (2D) Fourier transform of these images to visualize the diffraction of ice around 0.27 Å^−1^ (often described as the ‘ice ring at 3.7 Å’)^24^. We excluded regions contaminated with large exogenous ice particles to restrict our analysis to electron-transparent regions of the lamellae (Fig. 5a). Performing peak detection analysis in the water ring region of the 2D power spectra of acquired images allowed categorizing each of the 5,968 sampled positions as either ‘unambiguously vitreous’, ‘semi-crystalline’ or ‘unambiguously crystalline’ (Extended Data Fig. 10). Our analysis showed that 77 ± 14% of positions were vitreous in cells prepared with EasyGrid (*n* = 16 lamellae) compared to 11 ± 24% in cells prepared with standard plunge-freezing (*n* = 20 lamellae; Fig. 5b). These results indicate that cells vitrified with EasyGrid displayed improved ice quality compared to plunge-frozen preparations, paving the way to further ultrastructural characterization of large cellular specimens. Additionally, the images of vitrified cells recorded within the cryoFIB-milling instrument before lamellae production (Extended Data Fig. 10) exhibit no observable morphological differences attributable to the EasyGrid sample preparation technique when compared to plunge-frozen cells.

Finally, we reconstructed a ribosome structure at a resolution of 6.8 Å by applying sub-tomogram averaging workflow to EasyGrid-vitrified HeLa cell lamellae, which were also used for ice quality assessment (Fig. 4e,f). This result demonstrates the applicability of EasyGrid-based cell vitrification for high-resolution in situ cryo-ET studies.

## Discussion

Here, we present EasyGrid, a modular technology that addresses major challenges of cryo-EM sample preparation and enables systematic, high-throughput cryo-EM/ET. We first demonstrated how EasyGrid allows optimizing macromolecular samples for SPA and subsequently determined five structures of which three at about 2 Å resolution. We then showed how EasyGrid improves the vitrification of large cells, thus increasing the throughput and reproducibility of cryo-ET workflows. The versatility of EasyGrid and the ability to automatically assess sample quality with EGC make this technology stand out from other grid preparation instruments.

Sample spreading in EasyGrid differs from blotting, wicking and pin-printing. Blotting is slow (seconds) and it possibly releases contaminants on grids^25^. By contrast, our pressure-wave system allows spreading samples within tens to hundreds of milliseconds and does not leach particles in spread samples. Pressure-wave-based spreading did not prevent us from solving macromolecular structures at ~2 Å resolution, suggesting that EasyGrid is compatible with high-resolution SPA analysis. Furthermore, we demonstrate that the pressure-wave-based sample spreading in EasyGrid is well suited for preparing large adherent cells for cryo-ET while preserving their ultrastructure, making it a versatile tool for both cryo-EM and cryo-ET workflows. In contrast, to our knowledge, there is no evidence that wicking or pin-printing methods are suitable for such preparations.

EasyGrid robotics ensure precise grid positioning, accurate timing of operations and consistent repeatability, which minimizes variability at each step of sample preparation compared to semi-automated preparation. Precise control over SPA sample delivery (±50 pl) and critical timing, such as the delay between spreading and vitrification (minimum 200 ms, adjustable with ±1-ms accuracy), enables fine-tuning of sample thickness. This is achieved through tailored dispensing and/or regulated evaporation of the specimen on the grid before freezing; however, chaotic phenomena (for example the microfluidic behavior of SPA samples, the seeding of cells on cryo-ET grids and the nucleation of ice crystals) make each vitrified sample inherently unique. To address this irreducible sample variability, we propose automated sample preparation combined with integrated quality control.

EGC provides grid atlases to facilitate sample screening for optimal ice thickness. The current version provides average ice-thickness measurements per grid square but cannot yet resolve thickness within individual foil holes. To improve measurement accuracy, we are introducing higher magnification optics (up to ×50), enabling direct measurement of ice thickness at the hole level. Additionally, the next-generation instrument will incorporate a fluorescence microscopy module, allowing for the collection of valuable information from cellular samples. Once integrated with a neural network trained for atlas analysis, EGC will be instrumental in fully automating grid optimization.

To address samples that are currently difficult to prepare for SPA, EasyGrid (through its highly accurate control of preparation parameters) allows automated screening of biochemical conditions, enabling optimization of samples with incrementally varying composition, similar to protein crystallization platforms. This workflow, however, has not yet been implemented. One could combine this functionality with sample mixing^26^ to generate libraries of samples in which target macromolecules interact with arrays of selected ligands for example to develop new drugs. Further progress includes the insertion of a light injection line in the sample spreading module and/or the implementation of time-resolved mixing procedures^26–28^ to trigger (photo-chemical) reactions on grids with a time resolution in the millisecond range^29–31^. These upgrades will enable us to capture transient reaction intermediates using time-resolved, stop-motion cryo-EM, providing critical insights into the biochemical mechanisms underlying complex biomolecular processes.

Finally, we aim at transferring the EasyGrid technology to other structural biology laboratories and facilities. EasyGrid’s modular design allows flexible implementation, either as a complete platform or as standalone units with specific functionalities. For example, EGC alone can support laboratories with limited cryo-EM access by enabling grid screening and ensuring sample quality meets the standards of high-end cryo-EM facilities. When deployed as a full platform, EasyGrid enhances cryo-EM/ET facility capabilities by streamlining sample screening, enabling jet vitrification of cells, and automating the optimization of both cellular and biochemical samples. The instrument is currently being assessed for cellular sample vitrification for nano X-ray imaging applications.

## Methods

### Apoferritin preparation parameters

Purified apoferritin solution was prepared at the Protein Expression and Purification Facility at the European Molecular Biology Laboratory (EMBL) Heidelberg. In brief, *Escherichia* *coli* DL21 (DE3) were transformed with a pET-24a vector coding for FTH1 (ferritin heavy chain 1). The *FTH1* expression construct was transformed into *E.* *coli* BL21(DE3) RIL^+^ cells. A 20-ml pre-culture was grown overnight at 37 °C and used to inoculate 1 l of TB-FB auto-induction medium supplemented with glucose, galactose, MgSO_4_ and kanamycin (10-ml pre-culture per liter of medium) in 5-l baffled flasks. Cultures were grown at 37 °C until an OD_600_ of approximately 0.8 was reached. The temperature was then reduced to 18 °C, and expression was continued overnight. Cells were collected by centrifugation and the pellets were stored at −20 °C until further use. Cell pellets obtained from 1 l of culture were resuspended in 50 ml of Buffer A (30 mM HEPES, pH 7.5, 300 mM NaCl and 2 mM MgSO_4_) supplemented with cOmplete protease inhibitor cocktail (Roche). Cells were lysed using a Microfluidizer (five passes), with samples maintained on ice between cycles. Cell debris were removed by centrifugation at 180,000*g* for 30 min at 4 °C using a Ti45 rotor (Beckman ultracentrifuge). The supernatant was filtered and subsequently subjected to heat treatment at 70 °C for 10 min. Following heat treatment, precipitated material was removed by centrifugation at 166,000*g* for 30 min at 4 °C (Ti45 rotor). Solid ammonium sulfate was added to the clarified supernatant to a final concentration of 33.25 g 100 ml^−1^. The solution was stirred on ice for 10 min and centrifuged at 78,000*g* for 20 min at 4 °C (Ti45 rotor). The resulting pellet was resuspended in 20 ml of precipitation Buffer B (20 mM HEPES, pH 7.5 and 300 mM NaCl) and dialyzed overnight at 4 °C against 1 l of precipitation Buffer B. Next, anion exchange chromatography was performed using a 5-ml HiTrap Q column (GE Healthcare). The column was equilibrated with Buffer C (20 mM HEPES, pH 7.5, 75 mM NaCl) before sample application. Following overnight dialysis, the protein solution was diluted 1:5 with dilution buffer (20 mM HEPES, pH 7.5) to a final volume of 100 ml and loaded onto the column at a flow rate of 1 ml min^−1^. After sample application, the column was washed with Buffer C until a stable baseline was reached. Bound protein was eluted using a linear gradient to 100% Buffer D (20 mM HEPES, pH 7.5 and 1 M NaCl) over 75 ml. Fractions of 5 ml were collected during elution. Finally, the sample was injected into a gel filtration column Superdex S200 16/60, which was run with a flow rate of 1 ml min^−1^ with SEC buffer (20 mM HEPES, pH 7.5 and 100 mM NaCl). The best fractions were combined and diluted to 3.8 mg ml^−1^. In total, 19.3 mg of mFTH1 was purified with a A260/A280 of 0.56.

Apoferritin (at a concentration of 3.8 mg ml^−1^) was dispensed without dilution in two consecutive steps on holey-carbon R2/2 300 mesh copper grids (Quantifoil) plasma-treated ten times in EasyGrid right before sample dispensing. First, the grids were coated with a layer of sample by spreading a line of 100 droplets dispensed on the grid with a 2.2-bar pressure wave applied on both sides of the grid for the duration of 30 ms, and then leaving the grid to dry for 10 s at room humidity to clear up the foil holes from coating solution. This coat ensured that the next sequence of dispensing and spreading would produce a thin film of solution. Another line of 100 droplets was then dispensed on coated grids and spread at 20 °C and >90% humidity with a 2.2-bar pressure wave for the duration of 30 ms. No delay was added between sample spreading and jet vitrification of the grids. A total of two iterations of the sample preparation optimization protocol (Supplementary Note 3) were required to establish the final preparation parameters. The grids were then shipped in cold nitrogen vapor to the EMBL Imaging Centre for cryo-EM imaging.

### Apoferritin image acquisition and processing

Data acquisition was carried out using a Titan Krios G4i TEM operated at an acceleration voltage of 300 kV and equipped with a Falcon4i direct electron detector and a SelectrisX energy filter (Thermo Fisher Scientific) operated in zero-loss mode with a filter slit size of 10 eV. A total of 4,200 micrographs were acquired at a nominal magnification of ×165,000, resulting in a calibrated pixel size of 0.731 Å. The TEM was operated with a spot size of 5 and each target was exposed for 4 s, accumulating a total dose of 40 e^−^ per Å^2^ in counting mode in 1,135 Electron Event Representation (EER) frames. The target defocus range was set between −1.6 and −0.6 μm. Motion correction of EER videos was performed with the CPU implementation of MotionCor2 from Relion4 (ref. ^32^). Initial contrast transfer function (CTF) estimation was performed using CTFFIND4 (ref. ^33^). Particle picking was performed using Gautomatch^34^. Particle extraction and Bayesian polishing were performed with Relion3 (ref. ^32^). All other cryo-EM data processing steps were performed in cryoSPARC^35^ v.4.2.1. Conversion of cryoSPARC ‘.cs’ files to Relion ‘.star’ files was performed using UCSF pyem^36^ in combination with in-house bash scripts^37^. All final homogeneous refinements conducted in cryoSPARC^35^ had options ‘per-particle defocus’, ‘per-group CTF parameter optimization’ and ‘Ewald Sphere Correction’ switched on, resulting in a structure resolved at 1.94 Å (FSC threshold 0.143) from 188,570 particles. Local resolution estimation was performed using Relion4^32^. Cryo-EM density renderings were generated with ChimeraX^38^.

### Ribosome preparation parameters

*Schizosaccharomyces* *pombe* K972 yeast cells grown overnight in yeast extract with supplements (YES; Formedium) medium was used to inoculate 500 ml YES to a density of OD_600nm_ = 0.05 and grown overnight at 30 °C while shaken at 225 rpm until they reached an OD_600nm_ of 1.5 to 2. Cells were collected by centrifugation at 3,000*g* for 5 min at room temperature and the resulting cell pellet was resuspended in sterile milliQ-H_2_O and transferred to a 50-ml centrifugation tube. Following a sedimentation at 3,000*g* for 5 min at room temperature, the cells were resuspended in lysis buffer (20 mM HEPES, pH 7.4, 100 mM KCl and 5 mM Mg(OAc)_2_ containing a protease inhibitor cocktail tablet from Roche) and sedimented again as above. The resulting pellets were resuspended in 2 ml of lysis buffer per 1 g wet weight and lysed using sterilized glass beads (ø3–5 mm) by four cycles of vortexing at 4 °C for 30 s, with 30 s of cooling on ice between each cycle. Following a sedimentation, as described above, of the cell debris and glass beads, the supernatant was subjected to a clearing spin at 12,000*g* for 10 min at 4 °C. The cleared lysate was loaded onto a 50% (*w*/*v*) sucrose cushion containing lysis buffer and subsequently centrifuged at 55,000 rpm (SW55Ti rotor, 368,000*g*) for 20 h at 4 °C. Following centrifugation, the ribosome pellet was carefully resuspended in 150 µl cryo-EM buffer (20 mM HEPES pH 7.4, 60 mM KCl and 5 mM Mg(OAc)_2_) and diluted to a concentration of 250 μg µl^−1^ as determined by spectrophotometric analysis (A_260nm_). Freshly prepared ribosome samples were applied to cryo-EM grids as follows:

Quantifoil R2/2 300 mesh copper grids were first plasma-treated 15 times in EasyGrid, then coated on half of their surface with Blue Dextran (BD) (Merck; reference D4772) by dispensing a horizontal line of 100 droplets of 2 mg ml^−1^ BD at a vertical offset of +0.2 mm from the central axis of the grid and spreading it with a 2.2 bar pressure wave at >80% humidity and 4 °C for the duration of 30 ms, then leaving the grid to dry at room humidity and temperature for 10 s to clear up the foil holes from coating solution. After coating, pure ribosome solution was applied on the grids as a line of 100 droplets dispensed at an offset of +0.3 mm from the central axis of the grid and spread with a pressure wave for the duration of 50 ms at >80% humidity and 4 °C. No delay was added between sample spreading and jet vitrification of the grids. One iteration of the sample preparation optimization protocol (Supplementary Note 3) was required to establish the final preparation parameters. The grids were then shipped in cold nitrogen vapor to the EMBL Imaging Centre for cryo-EM imaging.

### Ribosome image acquisition and processing

Data acquisition was carried out using a Titan Krios G4i TEM operated at an acceleration voltage of 300 kV and equipped with a Falcon4i direct electron detector and a Selectris energy filter (Thermo Fisher Scientific) operated in zero-loss mode with a filter slit size of 10 eV. A total of 9,747 micrographs were acquired at a nominal magnification of ×165,000, resulting in a calibrated pixel size of 0.731 Å. The TEM was operated with a spot size of 4 and each target was exposed for 1.6 s, accumulating a total dose of 48 e^−^/Å^2^ in counting mode in 504 EER frames. The target defocus range was set between −1.6 and −0.6 μm. Motion correction of EER videos was performed with the CPU implementation of MotionCor2 from Relion4 (ref. ^32^). Initial CTF estimation was performed using CTFFIND4 (ref. ^33^). Particle picking was performed using in-house Python scripts applying bandpass filtering and peak detection to detect ribosomal particles in the micrographs^39^. Particle extraction and 3D classification with alignment were performed with Relion4. All other cryo-EM data processing steps were performed in cryoSPARC^35^ v.4.2.1. All final homogeneous refinements were carried out in cryoSPARC^35^ with per-particle defocus and per-group CTF parameter optimization as well as Ewald Sphere Correction switched on, resulting in a structure resolved at 2.43 Å (FSC threshold 0.143) from 135,490 particles. Local resolution estimation was performed using Relion4. Cryo-EM density renderings were generated with ChimeraX^38^.

### Rhodopsin preparation parameters

The KR2 rhodopsin was produced as previously described^20^. In brief, KR2-coding DNA (*NaR* gene from *Krokinobacter eikastus*) was first optimized for *E.* *coli* codons using GeneArt (Thermo Fisher Scientific). That gene was then synthesized commercially (Eurofins) and subcloned into a pET15b plasmid with a C-terminal 6xHis-tag. *E.* *coli* cells of C41 strain were transformed with pET15b plasmid containing the *KR2* gene. Transformed cells were grown at 37 °C in shaking baffled flasks in the auto-inducing medium ZYP-5052 (ref. ^40^) containing 10 mg l^−1^ ampicillin. Cells were induced at an OD_600_ of 0.6–0.7 with 1 mM isopropyl-β-D-thiogalactopyranoside (IPTG). Subsequently, 10 μM all-*trans*-retinal was added and the incubation continued for 3 hours. The cells were collected by centrifugation at 5,000*g* for 20 min. Collected cells were disrupted in an M-110P Lab Homogenizer (Microfluidics) at 12,000 psi in a buffer containing 20 mM Tris-HCl, pH 7.5, 5% glycerol, 0.5% Triton X-100 (Sigma-Aldrich) and 50 mg l^−1^ DNase I (Sigma-Aldrich). The membrane fraction of the cell lysate was isolated by ultracentrifugation at 125,000*g* for 1 h at 4 °C. The pellet was resuspended in a buffer containing 50 mM Tris-HCl, pH 8.0, 0.2 M NaCl and 1% *n*-dodécyl-β-D-maltoside (DDM, Anatrace, Affymetrix) and stirred overnight for solubilization. The insoluble fraction was removed by ultracentrifugation at 125,000*g* for 1 h at 4 °C. The supernatant was loaded on an Ni-NTA column (QIAGEN), and the protein was eluted in a buffer containing 50 mM Tris-HCl, pH 8.0, 0.2 M NaCl, 0.4 M imidazole and 0.1% DDM. The eluate was subjected to size-exclusion chromatography on a Superdex 200 Increase 10/300 GL (Cytiva) in a buffer containing 50 mM Tris-HCl, pH 8.0, 0.2 M NaCl and 0.05% DDM. In the end, the protein was concentrated to 60 mg ml^−1^ using 100,000 MWCO Amicon Ultra-15 filters (Millipore) and stored at –80 °C. For the preparation of the cryo-EM grids the protein was diluted to the concentration of 200 nM using the buffer containing 50 mM Tris-HCl, pH 8.0, 0.1 M NaCl and 0.03% DDM.

Quantifoil R2/2 300 mesh copper grids were first plasma-treated 15 times in EasyGrid, then coated twice on half of their surface with BD (Merck; ref. D4772) by dispensing a horizontal line of 100 droplets of 2 mg ml^−1^ BD at a vertical offset of +0.2 mm from the central axis of the grid and spreading it with a 2.2-bar pressure wave at >80% humidity and 4 °C for the duration of 30 ms, then leaving the grid to dry at room humidity and temperature for 10 s. Rhodopsin solution at 10 mg ml^−1^ was then applied on the grids as a line of 100 droplets dispensed at an offset of +0.3 mm from the central axis of the grid and spread with 80 ms 2.2-bar pressure wave at >90% humidity and 4 °C. A delay of 500 ms was added between sample spreading and jet vitrification of the grids. A total of two iterations of the sample preparation optimization protocol (Supplementary Note 3) were required to establish the final preparation parameters.

### Rhodopsin image acquisition and processing

Data acquisition was carried out using a Krios G3 TEM (Thermo Fisher Scientific, European Synchrotron Radiation Facility, Grenoble) operated at an acceleration voltage of 300 kV and equipped with a Gatan K3 direct electron detector and a Gatan Quantum energy filter operated in zero-loss mode with a filter slit size of 20 eV. A total of 6,671 40-frame videos were acquired at a nominal magnification of ×105,000, resulting in a calibrated pixel size of 0.839 Å. The TEM was operated with a spot size of 6 and each micrograph was exposed for 1.65 s, accumulating a total dose of 40.3 e^−^/Å^2^ in counting mode. The target defocus range was set between 0.8 and 2.4 μm, in steps of 0.2 μm. Image data processing was entirely performed using the cryoSPARC^35^ software (v.4.2.1). Patch motion correction was performed on the raw videos, followed by patch CTF estimation. After exposure curation, 4,748 micrographs were selected for further processing. Blob particle picking and particle extraction with a 320 × 320 pixel box size were performed, followed by a round of 2D classification. Five relevant 2D classes exhibiting different particle orientations were selected and used as templates for a subsequent template picking step. Then, 1,916,859 particles were picked and extracted using a 320 × 320 pixel box size, then binned to 160 × 160 pixel box size. Two additional rounds of 2D classification and particle selection were then performed, resulting in a homogenous set of 333,078 particles that were then unbinned. A nonsymmetrized ab initio initial model was generated using a subset of 179,700 particles and used to perform homogenous refinement with imposed C5 symmetry, resulting in a structure resolved at 2.36 Å (FSC threshold 0.143). Local CTF refinement and a final round of local refinement were performed, improving the resolution to 2.32 Å (FSC threshold 0.143). Cryo-EM density renderings were generated with ChimeraX^38^.

### INO80–0N80 sample preparation

INO80 was purified as previously described^41^. Likewise, the nucleosomes (0N80) were reconstituted using a salt gradient as previously described^41^. INO80 and 0N80 were mixed with an equimolar ratio (800 nM) and dialyzed to a binding buffer containing 30 mM HEPES–KOH, pH 7.5, 50 mM NaCl and 0.25 mM dithiothreitol at 4 °C, under agitation for 30 min. The INO80–0N80 mix was spun down for 10 min at 4 °C and at 12,000*g*, before addition of 0.05% of octyl-β-glucoside.

Quantifoil R2/2 300 mesh copper grids were first plasma-treated 15 times in EasyGrid, then coated on half of their surface with BD (Merck; ref. D4772) by dispensing a horizontal line of 100 droplets of 1 mg ml^−1^ BD by spreading it with a 2.0-bar pressure wave at >75% humidity and 4 °C for the duration of 30 ms while the grid was simultaneously lifted by the gripper of 1.5 mm. The grid was left to dry at room humidity and temperature for 30 s. INO80–0N80 solution was then applied onto the grids as a line of 100 droplets dispensed and spread over 100 ms, divided into ten pulses of 10 ms, with a 2.0-bar pressure wave at >75% humidity and 4 °C. The grid was lifted by 1.5 mm during the spreading process. A delay of 100 ms was added between sample spreading and jet vitrification of the grids. A total of five iterations of the sample preparation optimization protocol (Supplementary Note 3) were required to establish the final preparation parameters.

### INO80–0N80 image acquisition and processing

Data acquisition was carried out using a Glacios TEM (Thermo Fisher Scientific, EMBL, Grenoble) operated at an acceleration voltage of 200 kV and equipped with Falcon4i direct electron detector and a Gatan Quantum energy filter operated in zero-loss mode with a filter slit size of 20 eV. A total of 2,060 40-frame videos were acquired at a nominal magnification of ×100,000, resulting in a calibrated pixel size of 1.2 Å. The TEM was operated with a spot size of 6 and each micrograph was exposed for 7.9 s, accumulating a total dose of 60.0 e^−^/Å^2^ in counting mode. The target defocus range was set between 0.5 and 2 μm, in steps of 0.2 μm. Image data processing was entirely performed using the cryoSPARC^36^ software (v.4.2.1). Patch motion correction was performed on the raw videos, followed by patch CTF estimation. After exposure curation, 1,641 micrographs were selected for further processing. Blob particle picking and particle extraction with a 320 × 320 pixel box size were performed, followed by a round of 2D classification. Eleven relevant 2D classes exhibiting different particle orientations were selected corresponding to 98,403 particles and used to generate two 3D ab initio models. One model, built using 71,058 particles, was submitted to nonuniform refinement and resulted in a structure resolved at 4.93 Å (FSC threshold 0.143). Local CTF refinement estimated the resolution at 5.45 Å (FSC threshold 0.143). Cryo-EM density renderings were generated with ChimeraX^39^.

### Guanidinase sample preparation

The guanidinase from *Nitrospira* *inopinata* was prepared as previously described^42^. The sample was thawed on ice, diluted to 4 μM concentration in the buffer containing 20 mM HEPES–NaOH, pH 7.5 and 150 mM NaCl and was spun down for 10 min at 4 °C and at 12,000*g*.

Quantifoil R1.2/1.3 300 mesh copper grids were first plasma-treated five times in EasyGrid, then coated on half of their surface with BD (Merck; ref. D4772) by dispensing a horizontal line of 100 droplets of 1 mg ml^−1^ BD by spreading it with a 1.8-bar pressure wave at >85% humidity and 4 °C for the duration of 30 ms. The grid was left to dry at room humidity and temperature for 60 s. Guanidinase sample was then applied on the grids as a line of 100 droplets dispensed and spread with 120 ms with a 2.2-bar pressure wave at >85% humidity and 4 °C. A delay of 500 ms was added between sample spreading and jet vitrification of the grids. One iteration of the sample preparation optimization protocol (Supplementary Note 3) was required to establish the final preparation parameters.

### Guanidinase image acquisition and processing

Data acquisition was carried out using a Glacios TEM (Thermo Fisher Scientific, EMBL, Grenoble) operated at an acceleration voltage of 200 kV and equipped with Falcon4i direct electron detector and a Gatan Quantum energy filter operated in zero-loss mode with a filter slit size of 20 eV. A total of 693 40-frames videos were acquired at a nominal magnification of ×130,000, resulting in a calibrated pixel size of 0.878 Å. The TEM was operated with a spot size of 6 and each micrograph was exposed for 3.99 s, accumulating a total dose of 40.0 e^−^/Å^2^ in counting mode. The target defocus range was set between 0.75 and 2 μm, in steps of 0.25 μm. Image data processing was entirely performed using the cryoSPARC^35^ software (v.4.2.1). Patch motion correction was performed on the raw videos, followed by patch CTF estimation. After exposure curation, 502 micrographs were selected for further processing. Manual picking of 140 particles was performed from two micrographs followed by particle extraction with a 224 × 224 pixel box size and by a round of 2D classification. Relevant 2D classes were selected and used as templates for template particle picking that resulted in 468,313 picked particles. Four-times binned particles were extracted and cleaned by several rounds of 2D classification. A set of 228,937 particles was re-extracted with a 112 × 112 pixel box size (twice binned) and used to generate two 3D ab initio models followed by the heterogeneous refinement of the two models. One model, built using 135,214 particles, was submitted to homogeneous refinement and resulted in a structure resolved at 3.68 Å (FSC threshold 0.143). Particles, used to generate this model, were then re-extracted without binning with a 224 × 224 pixel box size, subjected to a final round of 2D classification and cleaning. Final set of 119,931 particles was used for homogeneous refinement with imposed D3 symmetry including optimization of per-particle defocus, resulting in a structure resolved at 3.09 Å (FSC threshold 0.143). Cryo-EM density renderings were generated with ChimeraX^39^.

### Cell culture, vitrification and lamella milling

HeLa Tokyo cells were grown at 37 °C in DMEM (Invitrogen) supplemented with penicillin, streptomycin and fetal bovine serum. SUM159 (human breast cancer) cells were maintained in DMEM/F-12 GlutaMAX (Thermo Fisher Scientific) supplemented with 5 mg ml^−1^ insulin (Cell Applications), 1 mg ml^−1^ hydrocortisone (Sigma), 5% FBS (v/v), 50 mg ml^−1^ streptomycin and 50 U ml^−1^ penicillin. SiO_2_ R1.2/20 gold grids (Quantifoil) were micropatterned using a PRIMO device (Alveole) following a previously described protocol^23^. To seed cells on grids, confluent cell cultures were rinsed in PBS and treated with 0.5 ml trypsin for 2 min before seeding them onto grids. For SUM159 cells, 4.0 × 10^5^ trypsinized cells were seeded onto grids in 35-mm low µ-dishes (ibidi) (4–5 grids per dish) and incubated for 20–30 min. After cell attachment, grids were transferred to another dish with fresh DMEM. The next day, grids were inserted one at a time into the EasyGrid loading station, then immediately picked up by the gripper robot and transferred to the high-humidity chamber maintained at 30 °C and >75% humidity to limit cell stress induced by DMEM evaporation. The grids prepared with EasyGrid were then treated with an 1.8-bar pressure wave for the duration of 400 ms and immediately jet-vitrified. The total duration between picking a cell-seeded grid from the culture dish and jet-freezing it did not exceed 40 s. The plunge-frozen grids were prepared with a Leica GP2 plunger (Leica Microsystems) maintained at 37 °C and >90% humidity. Grids seeded with SUM159 cells were supplemented with 3 µl of culture medium before blotting. Grids were blotted for 2 s from the reverse side of the grid, then plunged into liquid ethane maintained at −182 °C and stored in LN_2_ until further processing. Grids were screened in EGC and inserted into an Aquilos FIB/SEM (Thermo Fisher). Lamellae and stress-relief trenches^43^ were automatically milled at mid-height of cells located at the center of grid squares with an angle of 13° using serialFIB^44^, followed by manual polishing down to ≈190-nm thickness.

### In-cell cryo-ET

Tilt series were acquired in 14 lamellae of HeLa cells prepared with EasyGrid, using a Titan Krios G3i TEM (Thermo Fisher) operated at 300 kV and equipped with a Bioquantum energy filter and a K3 direct electron detector (Gatan) operated in zero-loss mode. On each grid, lamellae were mapped with a pixel size of 28 Å, −100 μm defocus, a 70-μm objective aperture and a 30 eV energy slit. The 14 lamellae were mapped for TS acquisition in SerialEM^45^ following an established protocol^46^. Data were acquired at a nominal magnification of ×42,000 resulting in a calibrated pixel size of 2.075 Å on the camera. A 50-μm C2 and a 70-μm objective aperture were inserted and the width of the energy filter slit was set to 10 eV. The TEM was operated in nanoprobe mode at a spot size of 7. TS were acquired using dose-symmetric tilt-scheme with a 2°-tilt increment and tilt angles ranging from −35° to +61° centered on lamella pretilt (+13°). Videos were acquired in counting mode over a 270-ms exposure time with a total accumulated dose of ~2.63 e^−^ per Å^2^ per video (~15 e^−^ per px s^−1^ over an empty area on the camera level) and saved in the TIF file format. Eleven frames were saved in each raw tilt image. The accumulated dose of each TS amounted to 130 e^−^ per Å^2^. The target defocus was set from −2 µm to −4 μm with 0.5-μm steps between TS. Tomograms were aligned and reconstructed using AreTomo^47^ v.1.1.1 and visually inspected using IMOD^48^ v.4.12.17.

### Ribosome STA processing

Particle positions were identified using a custom blob-picking script (see ‘Code Availability’ section). Tomograms were normalized to zero mean and unit variance, then bandpass filtered by subtracting two Gaussian-blurred volumes: a low-pass component (σ = 3 pixels) and a high-pass component (σ = 7 pixels). To enhance features, the filtered volumes were block-reduced (binning factor = 1), and thresholded at –0.55 × s.d. Blob-like densities were segmented and reduced to 3D skeletal features using a skeletonization algorithm, and the resulting coordinates were extracted as candidate particle centers.

Coordinates were trimmed to exclude positions within 30 pixels of tomogram borders in *x*–*y* and *z*. Proximity-based cleaning was applied to remove detections within 8 pixels of each other. Final particle coordinates were exported as .txt files, and quality control volumes representing each processing step were generated to aid visual inspection.

Picked particles were imported into M for structure determination. Initial denoising was followed by iterative geometric and CTF refinement, using combinations of ImageWarp (with grids 3 × 3, 4 × 4 and 6 × 6), particle pose and stage angle optimization, and volume warping with coarse and fine 3D grids (Table 1). A final round of defocus refinement was performed using a coarse spatial grid to improve local CTF modeling. The final map reached a resolution of 6.8 Å, estimated by gold-standard FSC (0.143 criterion).

**Table 1:** M refinement iterations

Iteration step	ImageWarp grid	Particle poses	Stage angles	Volume warp grid	Defocus	Grid search	Resolution (Å)
0	-	-	-	-	✗	✗	8
1	3×3	✗	✗	-	✗	✗	8.3
2	4×4	✗	✗	-	✗	✗	8.3
3	4×4	✓	✓	✗	✗	✗	7.2
4	4×4	✓	✓	4×4×8×7	✗	✗	6.9
5	6×6	✓	✓	6×6×8×7	✗	✗	6.8
6	-	✗	✗	✗	✓	✓	6.8
7	6×6	✓	✓	6×6×8×7	✓	✓	6.8

### Sample thickness mapping in EasyGrid Control

Hologram acquisition and analysis with EGC is described in Supplementary Note 1.

### Ice quality assessment by cryo-EM raster imaging of lamellae

Tokyo HeLa cells were either plunge-frozen with a GP2 plunger (Leica) or prepared with EasyGrid, followed by lamella milling as described above. Lamellae were then imaged using a Titan Krios G4i TEM (Thermo Fisher) operated at 300 kV and equipped with a SelectrisX energy filter and a Falcon4i direct electron detector (Thermo Fisher) operated in zero-loss mode. A raster of TEM images spaced by 1 µm from one another and avoiding ice-contaminated areas and the platinum layer at the front of the lamella were acquired on each lamella with a pixel size of 1.189 Å and a total dose of 20 e^−^ per Å^2^. Five images were acquired for each position to overcome possible drift, resulting in 18,160 images at 3,632 positions on 20 lamellae from 20 plunge-frozen cells from two different grids, and 11,688 images at 2,336 positions on 16 lamellae from 16 jet-vitrified cells from two different grids. Frames acquired in .eer format were motion-corrected and converted to.mrc files using Relion4^32^. The power spectra of these frames were computed using the IMOD function ‘clip spectrum’ and analyzed using Python scripts^39^. Spectral components around the 3.7 Å ring of amorphous ice scattering^24^ were masked and further analyzed. Power peaks were detected using signal thresholding with a fixed threshold for all considered images. Resulting hits were first eroded to limit false positives and then dilated to merge neighboring hits into peaks corresponding to the FFT data. Individual peaks were counted in each spectrum using connected component analysis. A crystallinity score was calculated by summing the peak counts for all five images per position. Positions with a crystallinity score <10 were automatically labeled ‘vitreous’, those >50 were labeled ‘crystalline’, and the others were labeled ‘semi-crystalline’ (Extended Data Fig. 8). Each image was then visually checked for the presence or absence of crystalline artifacts in the EM image and peaks in the FFT, and re-labeled by a trained microscopist to limit false attributions. This curation modified vitreous:semi-crystalline:crystalline proportions from 15:37:48 to 11:24:65 for plunge-frozen cells, and from 75:14:11 to 77:14:9 for jet-vitrified cells (Fig. 5b).

Lamellae were milled in SUM159 cells following a similar protocol. A total of *n* = 17 lamellae were milled in six grids of cells prepared with a GP2 plunger (Leica) and *n* = 18 lamellae were milled in four grids of cells prepared with EasyGrid. Qualitative scores from 0 to 5 were attributed to each lamella depending on crystallinity and usability (Extended Data Fig. 9).

### Reporting summary

Further information on research design is available in the Nature Portfolio Reporting Summary linked to this article.

## Online content

Any methods, additional references, Nature Portfolio reporting summaries, source data, extended data, supplementary information, acknowledgements, peer review information; details of author contributions and competing interests; and statements of data and code availability are available at 10.1038/s41592-026-03127-5.

## Supplementary information

Supplementary Information Technical description of the EasyGrid and EasyGrid Control instruments. Supplementary results on the behavior of the pressure-wave-based spreading technique. Sample optimization protocol used on the EasyGrid system.

Reporting Summary

Peer Review File

Supplementary Video 1 Video showing the grid preparation process with the EasyGrid instrument.

## Source data

Source Data Fig. 2 Original images of the figure in high resolution.

Source Data Fig. 3 Original images of the figure in high resolution.

Source Data Fig. 4 Original images of the figure in high resolution.

Source Data Fig. 5 Original images of the figure in high resolution.

Source Data Extended Data Fig. 1 Original images of the figure in high resolution.

Source Data Extended Data Fig. 8 Original images of the figure in high resolution.

Source Data Extended Data Fig. 10 Original images of the figure in high resolution.

## Data Availability

The apoferritin structure was deposited in the Electron Microscopy Data Bank (EMDB) under accession code EMD-19880. The rhodopsin structure and model were deposited in the Protein Data Bank under accession code 8RQ5, and the EMDB under accession code EMD-19434. The cryo-EM structure of guanidinase was deposited in the EMDB under accession code EMD-56607. The cryo-EM structure of INO80 was deposited in the EMDB under accession code EMD-56821. The cryo-EM structure of ribosome was deposited in the EMDB under accession code EMD-56855. The structure of ribosome obtained with STA was deposited in the EMDB under accession code EMD-56861. Raw Cryo-EM data corresponding to the presented structures are stored on tapes and are available upon request from the authors. Source data are provided with this paper.

## Code availability

Source code for the ice quality quantification and blob picking is available on Zenodo at 10.5281/zenodo.20130640 (ref. ^49^) and on GitHub at https://git.embl.de/gemin/easygrid.

## References

[1] Wu, M. & Lander, G. C. Present and emerging methodologies in cryo-EM single-particle analysis. *Biophys. J.* **119**, 1281–1289 (2020). 32919493 10.1016/j.bpj.2020.08.027 PMC7567993

[2] Wan, W. & Briggs, J. A. Cryo-electron tomography and subtomogram averaging. *Methods Enzymol*. **579**, 329–367 (2016). 27572733 10.1016/bs.mie.2016.04.014

[3] Tegunov, D., Xue, L., Dienemann, C., Cramer, P. & Mahamid, J. Multi-particle cryo-EM refinement with M visualizes ribosome-antibiotic complex at 3.5 A in cells. *Nat. Methods* **18**, 186–193 (2021). 33542511 10.1038/s41592-020-01054-7 PMC7611018

[4] Saibil, H. R. Cryo-EM in molecular and cellular biology. *Mol. Cell* **82**, 274–284 (2022). 35063096 10.1016/j.molcel.2021.12.016

[5] deGennes, P.-G. Wetting: statics and dynamics. *Rev. Mod. Phys.* **57**, 827–863 (1985).

[6] Weissenberger, G., Henderikx, R. J. M. & Peters, P. J. Understanding the invisible hands of sample preparation for cryo-EM. *Nat. Methods* **18**, 463–471 (2021). 33963356 10.1038/s41592-021-01130-6

[7] Zheng, L. et al. Uniform thin ice on ultraflat graphene for high-resolution cryo-EM. *Nat. Methods* **20**, 123–130 (2023). 36522503 10.1038/s41592-022-01693-y PMC9834055

[8] Armstrong, M. et al. Microscale fluid behavior during cryo-EM sample blotting. *Biophys. J.* **118**, 708–719 (2020). 31952802 10.1016/j.bpj.2019.12.017 PMC7004840

[9] Xu, Y. & Dang, S. Recent technical advances in sample preparation for single-particle cryo-EM. *Front. Mol. Biosci.* **9**, 892459 (2022). 35813814 10.3389/fmolb.2022.892459 PMC9263182

[10] Jain, T., Sheehan, P., Crum, J., Carragher, B. & Potter, C. S. Spotiton: a prototype for an integrated inkjet dispense and vitrification system for cryo-TEM. *J. Struct. Biol.* **179**, 68–75 (2012). 22569522 10.1016/j.jsb.2012.04.020 PMC3378829

[11] Ravelli, R. B. G. et al. Cryo-EM structures from sub-nl volumes using pin-printing and jet vitrification. *Nat. Commun.* **11**, 2563 (2020). 32444637 10.1038/s41467-020-16392-5 PMC7244535

[12] Rubinstein, J. L. et al. Shake-it-off: a simple ultrasonic cryo-EM specimen-preparation device. *Acta Crystallogr. D* **75**, 1063–1070 (2019). 10.1107/S2059798319014372 PMC6889916 31793900

[13] Kontziampasis, D. et al. A cryo-EM grid preparation device for time-resolved structural studies. *IUCrJ* **6**, 1024–1031 (2019). 31709058 10.1107/S2052252519011345 PMC6830222

[14] Arnold, S. A. et al. Miniaturizing EM sample preparation: opportunities, challenges, and visual proteomics. *Proteomics* **18**, e1700176 (2018). 29441686 10.1002/pmic.201700176

[15] Dubochet, J. et al. Cryo-electron microscopy of vitrified specimens. *Q. Rev. Biophys.* **21**, 129–228 (1988). 3043536 10.1017/s0033583500004297

[16] Burstein, L. & Maurice, D. M. Cryofixation of tissue surfaces by a propane jet for electron microscopy. *Micron* **9**, 191–198 (1978).

[17] Plattner, H. & Knoll, G. in *The Science of Biological Specimen Preparation for Microscopy and Microanalysis* (eds Revel, J.-P. et al.) 139–146 (SEM Inc., 1984).

[18] Marquet, P. et al. Digital holographic microscopy: a noninvasive contrast imaging technique allowing quantitative visualization of living cells with subwavelength axial accuracy. *Opt. Lett.* **30**, 468–470 (2005). 15789705 10.1364/ol.30.000468

[19] Elbaum, M. & Lipson, S. G. How does a thin wetted film dry up? *Phys. Rev. Lett.* **72**, 3562–3565 (1994). 10056231 10.1103/PhysRevLett.72.3562

[20] Kovalev, K. et al. Molecular mechanism of light-driven sodium pumping. *Nat. Commun* **11**, 2137 (2020). 32358514 10.1038/s41467-020-16032-y PMC7195465

[21] Marko, M., Hsieh, C., Schalek, R., Frank, J. & Mannella, C. Focused-ion-beam thinning of frozen-hydrated biological specimens for cryo-electron microscopy. *Nat. Methods* **4**, 215–217 (2007). 17277781 10.1038/nmeth1014

[22] Rigort, A. et al. Focused ion beam micromachining of eukaryotic cells for cryoelectron tomography. *Proc. Natl Acad. Sci. USA* **109**, 4449–4454 (2012). 22392984 10.1073/pnas.1201333109 PMC3311327

[23] Toro-Nahuelpan, M. et al. Tailoring cryo-electron microscopy grids by photo-micropatterning for in-cell structural studies. *Nat. Methods* **17**, 50–54 (2020). 31740821 10.1038/s41592-019-0630-5 PMC6949126

[24] McMullan, G., Vinothkumar, K. R. & Henderson, R. Thon rings from amorphous ice and implications of beam-induced Brownian motion in single particle electron cryo-microscopy. *Ultramicroscopy* **158**, 26–32 (2015). 26103047 10.1016/j.ultramic.2015.05.017 PMC4584428

[25] Glaeser, R. M. Proteins, interfaces, and cryo-EM grids. *Curr. Opin. Colloid Interface Sci.* **34**, 1–8 (2018). 29867291 10.1016/j.cocis.2017.12.009 PMC5983355

[26] Dandey, V. P. et al. Time-resolved cryo-EM using Spotiton. *Nat. Methods* **17**, 897–900 (2020). 32778833 10.1038/s41592-020-0925-6 PMC7799389

[27] Berriman, J. & Unwin, N. Analysis of transient structures by cryo-microscopy combined with rapid mixing of spray droplets. *Ultramicroscopy* **56**, 241–252 (1994). 7831735 10.1016/0304-3991(94)90012-4

[28] Klebl, D. P., White, H. D., Sobott, F. & Muench, S. P. On-grid and in-flow mixing for time-resolved cryo-EM. *Acta Crystallogr. D* **77**, 1233–1240 (2021). 10.1107/S2059798321008810 PMC8489233 34605427

[29] Frank, J. Time-resolved cryo-electron microscopy: recent progress. *J. Struct. Biol.* **200**, 303–306 (2017). 28625887 10.1016/j.jsb.2017.06.005 PMC5732889

[30] Yoder, N. et al. Light-coupled cryo-plunger for time-resolved cryo-EM. *J. Struct. Biol.* **212**, 107624 (2020). 32950604 10.1016/j.jsb.2020.107624 PMC7959588

[31] Torino, S., Dhurandhar, M., Stroobants, A., Claessens, R. & Efremov, R. G. Time-resolved cryo-EM using a combination of droplet microfluidics with on-demand jetting. *Nat. Methods* **20**, 1400–1408 (2023). 37592181 10.1038/s41592-023-01967-z

[32] Scheres, S. H. Processing of structurally heterogeneous cryo-EM data in RELION. *Methods Enzymol.* **579**, 125–157 (2016). 27572726 10.1016/bs.mie.2016.04.012

[33] Rohou, A. & Grigorieff, N. CTFFIND4: Fast and accurate defocus estimation from electron micrographs. *J. Struct. Biol.* **192**, 216–221 (2015). 26278980 10.1016/j.jsb.2015.08.008 PMC6760662

[34] Jack Zhang Lab Gautomatch-v0.53. *GitHub* https://github.com/JackZhang-Lab/Gautmatch (2021).

[35] Punjani, A., Rubinstein, J. L., Fleet, D. J. & Brubaker, M. A. cryoSPARC: algorithms for rapid unsupervised cryo-EM structure determination. *Nat. Methods* **14**, 290–296 (2017). 28165473 10.1038/nmeth.4169

[36] Asarnow, D., Palovcak, E. & Cheng, Y. ECSF pyem v.0.5. *Zenodo*10.5281/zenodo.3576630 (2019).

[37] Fromm, S. A. miscEM v.0.1 *Zenodo*10.5281/zenodo.7529572 (2022).

[38] Pettersen, E. F. et al. UCSF ChimeraX: structure visualization for researchers, educators, and developers. *Protein Sci.* **30**, 70–82 (2020). 32881101 10.1002/pro.3943 PMC7737788

[39] Gemin, O. EasyGrid. *GitHub* https://git.embl.de/gemin/easygrid/ (2023).

[40] Studier, F. W. Protein production by auto-induction in high density shaking cultures. *Protein Expr. Purif.* **41**, 207–234 (2005). 15915565 10.1016/j.pep.2005.01.016

[41] Zhang, M. et al. Hexasome-INO80 complex reveals structural basis of noncanonical nucleosome remodeling. *Science* **381**, 313–319 (2023). 37384673 10.1126/science.adf6287

[42] Palatinszky, M. et al. Growth of complete ammonia oxidizers on guanidine. *Nature* **633**, 646–653 (2024). 39143220 10.1038/s41586-024-07832-z PMC11410670

[43] Wolff, G. et al. Mind the gap: micro-expansion joints drastically decrease the bending of FIB-milled cryo-lamellae. *J. Struct. Biol.* **208**, 107389 (2019). 31536774 10.1016/j.jsb.2019.09.006

[44] Klumpe, S. et al. A modular platform for automated cryo-FIB workflows. *eLife*10.7554/eLife.70506 (2021). 10.7554/eLife.70506 PMC8769651 34951584

[45] Mastronarde, D. N. Automated electron microscope tomography using robust prediction of specimen movements. *J. Struct. Biol*. **152**, 36–51 (2005). 16182563 10.1016/j.jsb.2005.07.007

[46] Turonova, B. et al. Benchmarking tomographic acquisition schemes for high-resolution structural biology. *Nat. Commun.* **11**, 876 (2020). 32054835 10.1038/s41467-020-14535-2 PMC7018747

[47] Zheng, S. et al. AreTomo: an integrated software package for automated marker-free, motion-corrected cryo-electron tomographic alignment and reconstruction. *J. Struct. Biol. X* **6**, 100068 (2022). 35601683 10.1016/j.yjsbx.2022.100068 PMC9117686

[48] Kremer, J. R., Mastronarde, D. N. & McIntosh, J. R. Computer visualization of three-dimensional image data using IMOD. *J. Struct. Biol.* **116**, 71–76 (1995). 10.1006/jsbi.1996.0013 8742726

[49] Gemin, O. & Papp, G. FFT-based ice quality quantitation in lamellae of plunge- or jet-vitrified cells. *Zenodo*10.5281/zenodo.20130640 (2026).

[50] Opron, K. & Burton, Z. F. Ribosome structure, function, and early evolution. *Int. J. Mol. Sci.*10.3390/ijms20010040 (2018). 10.3390/ijms20010040 PMC6337491 30583477

