# Supplementary material for: EasyGrid: a versatile platform for automated cryo-EM sample preparation and quality control
Source: Nat Methods. 2026 Jun 23;23(7):1359–67. doi: 10.1038/s41592-026-03127-5 (PMC13345917; doi:10.1038/s41592-026-03127-5)
Supplement: Supplementary file 1 — Technical description of the EasyGrid and EasyGrid Control instruments. Supplementary results on the behavior of the pressure-wave-based spreading technique. Sample optimization protocol used on the EasyGrid system. [file 41592_2026_3127_MOESM1_ESM.pdf]

nature methods

Supplementary information

https://doi.org/10.1038/s41592-026-03127-5

EasyGrid: a versatile platform for automated cryo-EM sample preparation and quality control

In the format provided by the authors and unedited

Supplementary Note

Table of Contents

SUPPLEMENTARY NOTE **1**

NOTE S1: DESCRIPTION OF EASYGRID INSTRUMENTS **2**

1. EASYGRID INSTRUMENT **2**

MAIN COMPONENTS 2
Fig. SN2. Photograph of the EasyGrid sample preparation platform *5*
GRAPHICAL USER INTERFACE (GUI) 6
Fig. SN3. Graphical User Interface of the EasyGrid machine *7*

2. EASYGRID CONTROL INSTRUMENT **8**

MAIN COMPONENTS 8
Fig. SN4. Schematics of the EasyGrid Control machine *9*
Fig. SN5. Photograph of the EasyGrid Control machine *10*
GRAPHICAL USER INTERFACE (GUI) 11
Fig. SN6. Graphical User Interface of the EasyGrid Control machine *11*
EGC includes a standalone grid analyzer tool (Suppl. Note Fig. SN7) to leverage grid measurements from EGC. The grid analyzer allows browsing squares and selecting them according to various criteria. The grid analyzer GUI components are the following: *11*
FIG. SN7. EASYGRID CONTROL GRID ANALYZER GUI 12
IMAGE ACQUISITION AND PROCESSING STEPS 13
Fig. SN8. EGC image acquisition and processing scheme *15*
Fig. SN9. Example images produced with EGC at intermediate processing steps *16*
Fig. SN10. Calibrated 10x thickness map of a cryo-EM grid prepared with EasyGrid *17*
PERFORMANCE ASSESSMENT 18
Measurement of empty sample holder grids *18*
Comparative measurement between EasyGrid Control and Cryo-Electron Tomography *19*
Fig. SN12. Comparative ice thickness measurement with EasyGrid Control and Cryo-ET. *19*

NOTE S2: ADDITIONAL INFORMATION ON PRESSURE WAVE BASED SPREADING SYSTEM BEHAVIOR **20**

EFFECT OF PRESSURE WAVE PARAMETERS 20
Effect of air pressure *20*
Effect of pulse duration *22*

NOTE S3. PREPARATION PARAMETERS OPTIMIZATION PROTOCOL **25**

Fig. SN16. Visual representation of the sample optimization protocol used on the EasyGrid instruments *26*

Note S1: Description of EasyGrid instruments

This document provides additional information about EasyGrid modules, EasyGrid Control (EGC) and their Graphical User Interface (GUI). The image processing pipeline implemented in EGC is also presented here.

1. EasyGrid instrument

Main components

To carry out tailorable sample preparation protocols, the EasyGrid sample preparation machine (Suppl. Note Figs. SN1&2) contains the following modules:

1. Grid handling module
   The grid handling module is an industrial pneumatic gripper (Schunk PGN-plus-P 40) mounted on a three axis cartesian robot equipped with absolute position encoders. The cartesian robot ensures precise positioning of the grid gripper at all stages of the preparation procedure. The gripper is equipped with custom-made epoxy jaws that limit heat transfer to the sample.

2. Drop dispensing module
   The drop dispensing module is composed of a four-axis robot (three cartesian axes and one rotation) that holds a temperature regulated dock, itself equipped with two microfluidic droplet dispenser heads manufactured by Microdrop (https://www.microdrop.de). Temperature regulation of the pipette dock is mediated by a Peltier element connected to a ventilated heat sink. The custom-designed drop dispenser pipettes have an internal nozzle diameter of 70um and are controlled by a Microdrop SL-T-4166 controller. To program sample dispensing, users can edit the number of drops and dispensing pattern geometry with the corresponding panel of the EasyGrid GUI (Fig. SN3, panel 11).

3. Pressure wave generator
   The pressure wave generator module is composed of two nozzles connected to a solenoid valve and supplied by standard oil-free compressed air. The supply pressure is typically set to 1.55 bar. The pressure waveforms are tailored by the user e.g.: number of pressure pulses, pulse duration, delay between pulses, grid movement during spreading. The typical waveform we used for preparing macromolecular solutions or adherent cells was a square wave lasting respectively ~80 ms or ~300 ms.

4. Preparation chamber
   The preparation chamber allows controlling environmental parameters during sample preparation. It is mounted on top of the vitrification and storage module, and it is separated from it by a trapdoor. The chamber is insulated with polyurethane foam and contains a water/air heat exchanger connected to a cryo-compact circulator (Julabo CF31). Humid air is supplied to the chamber from the humidifier module (described below) at a typical flow rate of 8 L/min to limit sample evaporation during the preparation process. The preparation chamber also features a lateral opening to insert drop dispenser heads during EasyGrid operation, and it is equipped with a camera & backlight system to monitor the sample preparation process.

5. Humidifier system
   The humidifier system is a nebulizer featuring a flow-regulated supply of compressed air, a water tank and an outlet of humid air. The tank of the humidifier system is manually filled with ultra-pure milli-Q water (Millipore). The resistor-based heating system installed on the tank allows heating up the tank to increase air humidity in the nebulizer. The humid air output by the system can be supplied to the preparation chamber to reach > 90% humidity around the grid within a few seconds.

6. Liquid ethane-based jet-vitrification module
   This module is composed of a closed tank partially immersed in liquid nitrogen (LN_2). The tank contains a heating resistor and a temperature sensor allowing fine temperature regulation of the liquid ethane to typically -180 ˚C. To fill the tank, a flow of 0.8 L/min gaseous ethane ($C_2H_{6,\ (g)}$) is progressively liquified in the cold tank. The capacity of the tank is ~40 mL, and filling it takes ~8min. Once the tank is filled, ethane can be jetted by pressurizing the tank with gaseous nitrogen $N_{2,\ (g)}$ at a flowrate of 0.5 L/min. The N_2 gas pushes liquid ethane towards the jetting apertures. These apertures face each other, allowing the collision of ethane jets. Jetted ethane is then collected in a recovery pool. Opening the recycling valve to pour the recovered ethane back into the reservoir allows reusing the same liquid ethane for a whole preparation session and removes the need for refilling the machine with ethane during operation. To empty the ethane tank, a ventilation system allows aspirating ethane away at any time point, typically at the end of a preparation session.

7. Cryo-storage module
   The cryo-storage plate is a carrousel set up in the dewar vessel just above the LN_2 level to maintain its temperature at ~ -185 °C and to limit ice contamination on stored grids by keeping them in N_2 vapor. The carrousel is driven by an absolute-encoded motor able to position box slots either under the user trapdoor to manually load/unload grid boxes, or under the preparation chamber to store prepared grids in cryo-boxes. The carousel features 11 slots to fit custom cryo-EM storage boxes equipped with eCryoID™ tags. Upon loading such a box on the carrousel, the unique ID of the box is automatically read and registered in the list of available containers. When storing a grid in a registered box, sample preparation metadata is saved in a text file and indexed to the box ID and slot number.

8. LN_2 regulation system
Regulation of the LN2 level in the storage dewar is carried out by a standalone LN2 micro-dosing pump (Norhof #915) that delivers small amounts of LN2 through an insulated tube based on feedback from a sensor placed in the dewar vessel.

9. Atmospheric plasma treatment system
Prior to grid preparation, grid hydrophilicity can be enhanced using an atmospheric plasma treatment system (Plasmatreat FG5001S). The plasma nozzle is set up on a fixed support and the grids are presented in front of nozzle using the gripper robot. The typical plasma treatment procedure is as follows: (1) plasma generation, (2) moving the grid back and forth through the plasma, perpendicularly to the nozzle, at a speed of 250 mm/s and at a distance of 12 mm, (3) plasma termination. The plasma torch outputs a power of 1 kVA at a frequency of 21kHz. Typically, ten to twenty of passages are executed during step (2) to adjust grid surface properties to specific sample solutions.

10. Sample storage module
The sample storage module is a thermo-regulated dock in which a standard low-profile PCR plate is inserted. The dock is equipped with a lid that can be opened and closed using a pneumatical jack to cover the PCR plate and improve thermal regulation, which is carried out by a Peltier element connected to a ventilated heat-sink. The temperature of the module can be freely set between 4 ˚C and 37 ˚C. Sample solutions are manually pipetted in the PCR plate, then automatedly picked-up by the drop dispensing module just before sample preparation. Typically, a volume of ~20 µL is used for purified protein samples.

11. Drop dispensing test module
Once the drop dispenser heads are filled, droplet dispensing parameters (i.e., piezo-electric waveform and voltage commanding individual droplet dispensing events) are tested in front of a camera to establish a monodisperse dispensing regime. The drop dispensing test module is composed of a camera and a stroboscopic backlight synchronized on drop dispensing events. An air-blowing nozzle connected to a cryo-compact circulator (Julabo CF31) through an air/water heat exchanger flows temperature-controlled compressed air at 5 L/min on the dispenser heads to keep them at a desired working temperature (4-37˚C). Drop dispensing is considered suitable for sample delivery on grids when individual drops of sample solution appear at the same position on successive stroboscopic images (monodisperse regime), and their trajectory is aligned with the central axis of the dispenser head (Fig. SN3, panel 5).

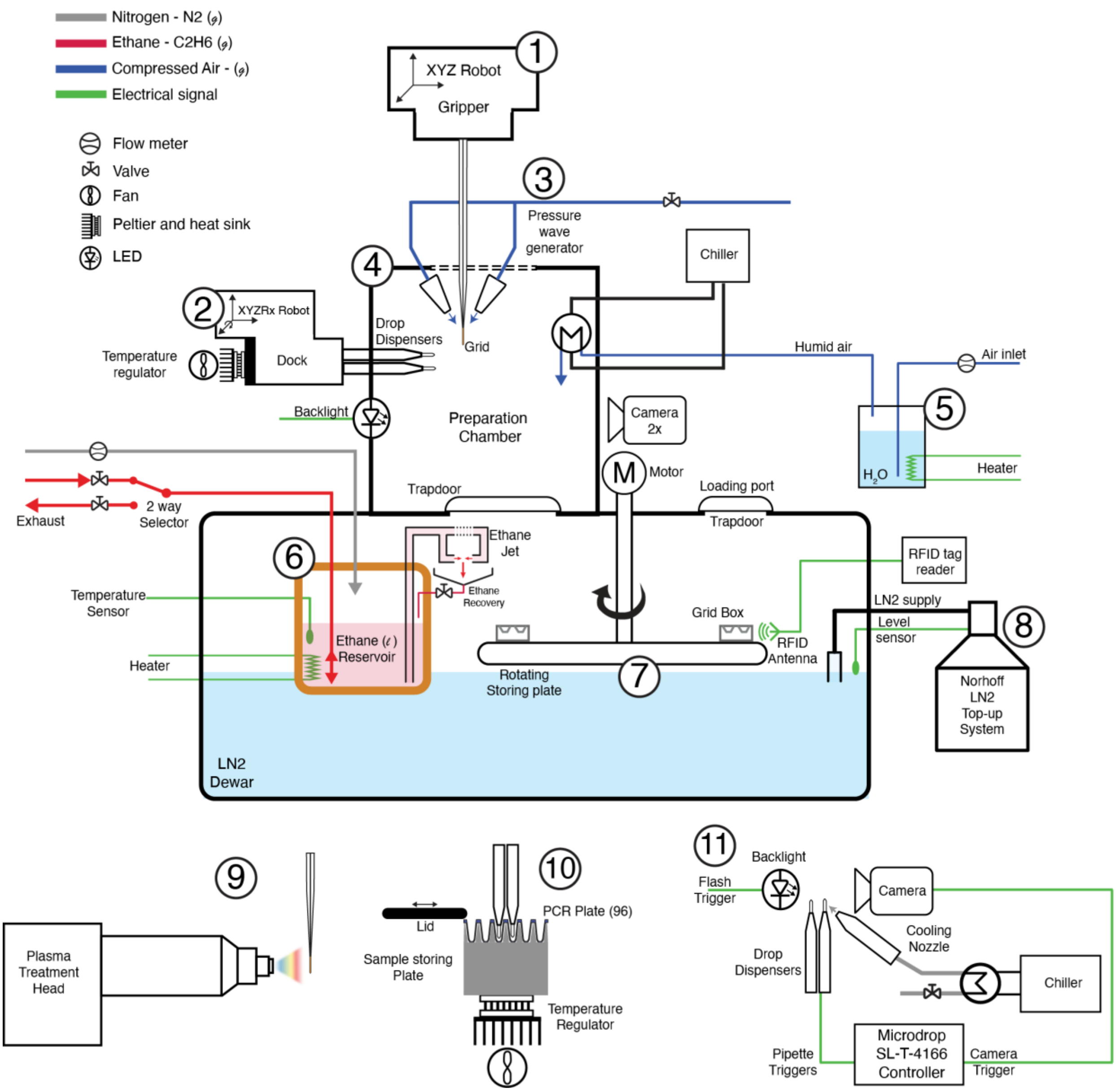

Fig. SN1. Schematics of the EasyGrid sample preparation machine

Simplified schematic diagram of the EasyGrid platform with components numbered as follows: (1) Grid handling module, (2) Drop dispenser module, (3) Pressure wave generator, (4) Preparation chamber, (5) Humidifier module, (6) Lliquid ethane jet-vitrification module, (7) Cryo-storage module, (8) LN_2 regulation system, (9) Plasma treatment module, (10) Sample storage plate and (11) Drop dispensing test module.

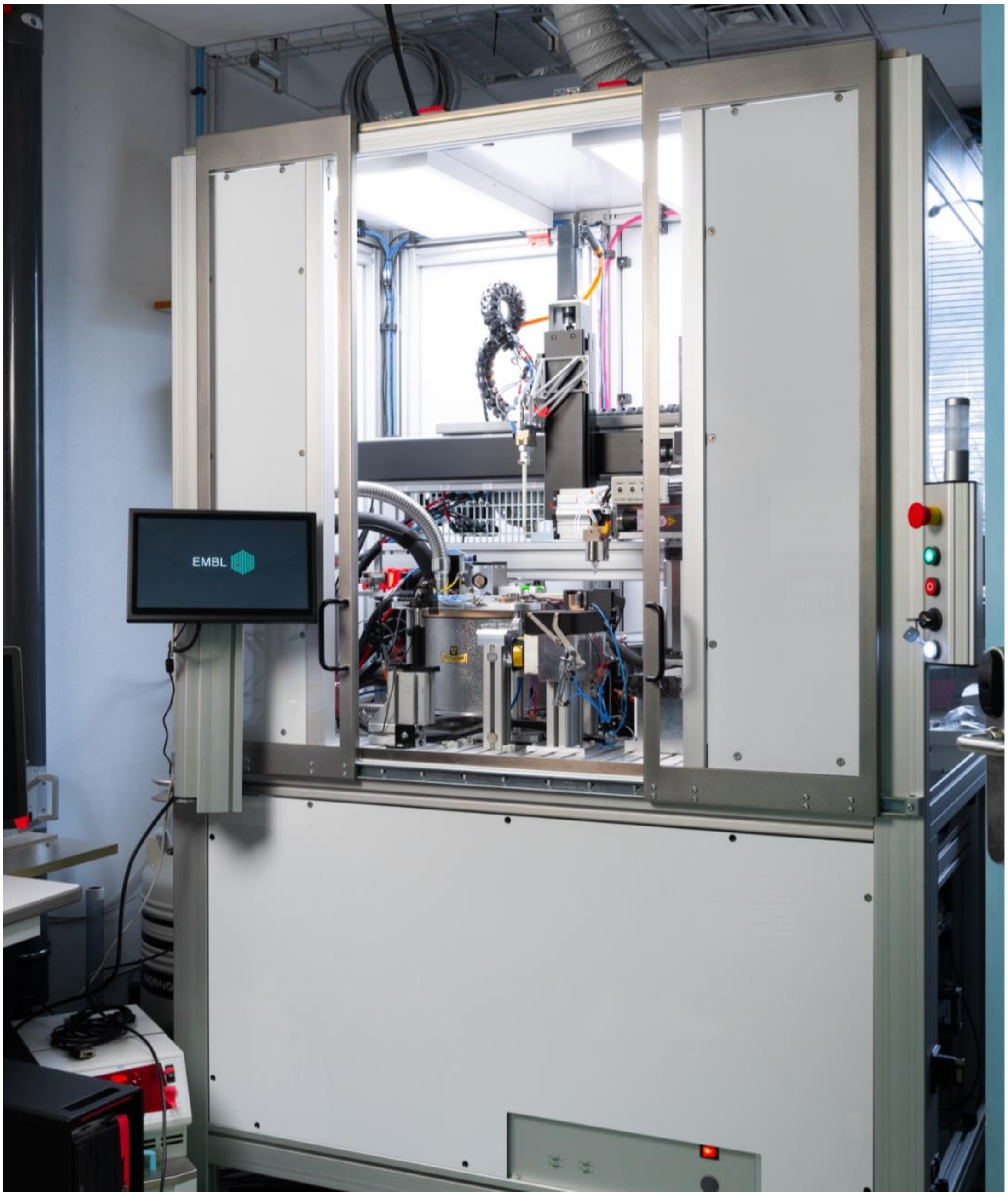

Fig. SN2. Photograph of the EasyGrid sample preparation platform

Graphical user interface (GUI)

The GUI of the EasyGrid machine (Suppl. Note Fig. SN3) provides access to the main functionalities of the system through an ergonomic interface. The main components of the GUI are the following:

1. Status panel
This panel displays the overall status of the machine as well as critical alarms and warnings.

2. Environment control panel
This panel permits setting and monitoring the temperature of the sample storage plate and drop dispenser heads, as well as the humidity and temperature of the preparation chamber.

3. Dewar control panel
This panel enables controlled cool-down (to cryogenic temperature) or heat-up (to room temperature) of the dewar and (re-)filling of the ethane jetting system.

4. High-level command panels
These panels allow parking the gripper and pipette robots in safety/default positions or aborting ongoing operations.

5. Drop dispense test panel
This panel displays stroboscopic movies of drops dispensed by either pipette. The dispensed drops can be visualized at different time points, milliseconds after dispensing, using the camera trigger delay slider.

6. Preparation chamber camera view
This panel displays live images taken in the preparation chamber to visualize the sample grid after drop dispensing and/or spreading.

7. Grid camera panel
This panel displays images recorded by two cameras set up perpendicularly at a “grid check” position outside of the humidity chamber. These views allow controlling for correct gripping of the sample support and the positioning of the gripper jaws. Besides, the rightmost image is overlaid with a pattern reflecting the current drop dispensing parameters (Fig. SN3, panel 11) to provide visual feedback about the planned dispensing procedure.

8. Sample preparation flowchart
This panel displays the status of the current sample preparation, guiding the user through the preparation steps i.e., loading of a new sample support, plasma treatment, optional dispense and spreading of a coating solution, dispense and spreading of the sample, vitrification and storage.

9. Grid loading panel
This panel displays the grid rack and permits to pick-up a new sample support from a given slot of the rack.

10. Plasma treatment control panel
This panel permits editing the number of times that the sample support will be driven through the locally generated plasma, and launching the plasma treatment procedure.

11. Drop dispensing and pressure wave generator control panel
In this panel, on the left, users can edit the drop dispensing procedure i.e., choose the dispensing mode (static for a discretized line or dynamic for continuous drop delivery along a trajectory), number of dispensing lines, number of drops per line, dispensing frequency and vertical/horizontal spacing between drop dispensing spots. On the right, users can edit the sample spreading procedure i.e., choose the number of pressure pulses applied to the grid, pulse duration, and delays either between pulses or between the sample spreading and vitrification procedures.

12. Grid storage and process launch panel
The user can select the box slot in which the prepared grid will be stored. Using the green buttons, they can initiate either a Prepare and Observe action (useful during optimization of the spreading process) or a Prepare and Store action, which performs sample dispensing and spreading, followed by jet vitrification and storage of the grid in the grid box at cryogenic temperature.

13. Pipeline panel
n this panel, the user can configure and initiate the entire preparation process, from empty grid pickup to sample storage, by selecting the desired steps and clicking the “Execute pipeline” button.

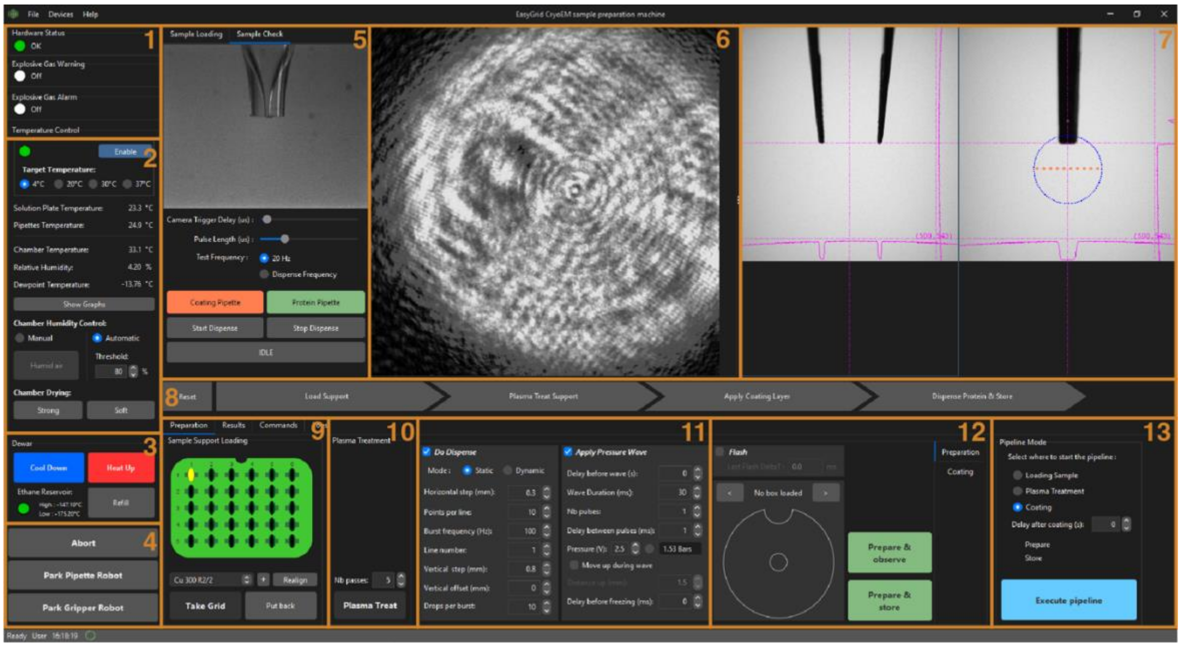

Fig. SN3. Graphical User Interface of the EasyGrid machine

Screenshot of the EasyGrid GUI with individual panels framed and numbered in orange as follows: (1) Status panel, (2) Environment control panel, (3) Dewar control panel, (4) High level commands, (5) Drop dispense test panel, (6) Preparation chamber camera view, (7) “Grid check” camera views with overlay of droplet dispensing pattern (red circles), (8) Flow chart of the sample preparation, (9) Grid loading panel, (10) Plasma treatment panel, (11) Drop dispensing control panel, (12) Pressure wave generator control panel.

2. EasyGrid Control instrument

Main components

EGC is a standalone cryo-EM sample quality assessment and ice thickness measurement device based on a digital holographic microscope (DHM) and a custom built cryo-observation column that permits imaging samples with the DHM at cryogenic temperature.
The system (Suppl. Note Figs. SN4&5) is composed of the following modules:

1. Motorized gripper

The motorized gripper is used to fetch samples one by one from storage boxes and to bring them to the DHM observation point. The gripper is made of epoxy resin to limit heat transfer to the sample during operation. Sample gripping is effected by a pneumatical jack and the vertical movement is carried out by a stepper motor. The typical travel time between the storage box and the observation point is ~5 seconds.

2. Cryo-observation column

The cryo-observation column keeps the sample at cryogenic temperature (below -170 ˚C) during the entire measurement process. It is composed of an external shell, an insulation layer, and an internal column. The bottom of the internal column is in physical contact with the liquid nitrogen to ensure efficient cooling. Plastic portholes of high optical quality $(\mathrm{scratch/dig}\ 20/10)$ are set up on both side of the observation point to allow for the DHM measurement beam to travel through the sample.

3. Cryo-storage module

Custom cryo-EM storage boxes equipped with eCryoID^{TM} tags can be inserted into the machine and placed on the storage carrousel through the user trapdoor. Upon insertion, the unique ID of the box is automatically read and registered in the EGC processing plan. The absolute-encoded motorized storage carrousel has a capacity for 10 boxes. Once loaded, the carousel can be rotated to place boxes underneath the observation column and screen the samples they contain.

4. Digital holographic microscope

The measurement device used for ice thickness control is a standard DHM (LyncéeTec T2103). The EGC system employs the software development kit (SDK) provided by LyncéeTec for DHM operation and data analysis.

5. LN_2 regulation system

Regulation of the LN_2 level in the storage dewar is carried out by a standalone LN_2 micro-dosing pump (Norhof #915) that delivers small amounts of LN_2 through an insulated tube based on feedback from a sensor placed in the dewar vessel.

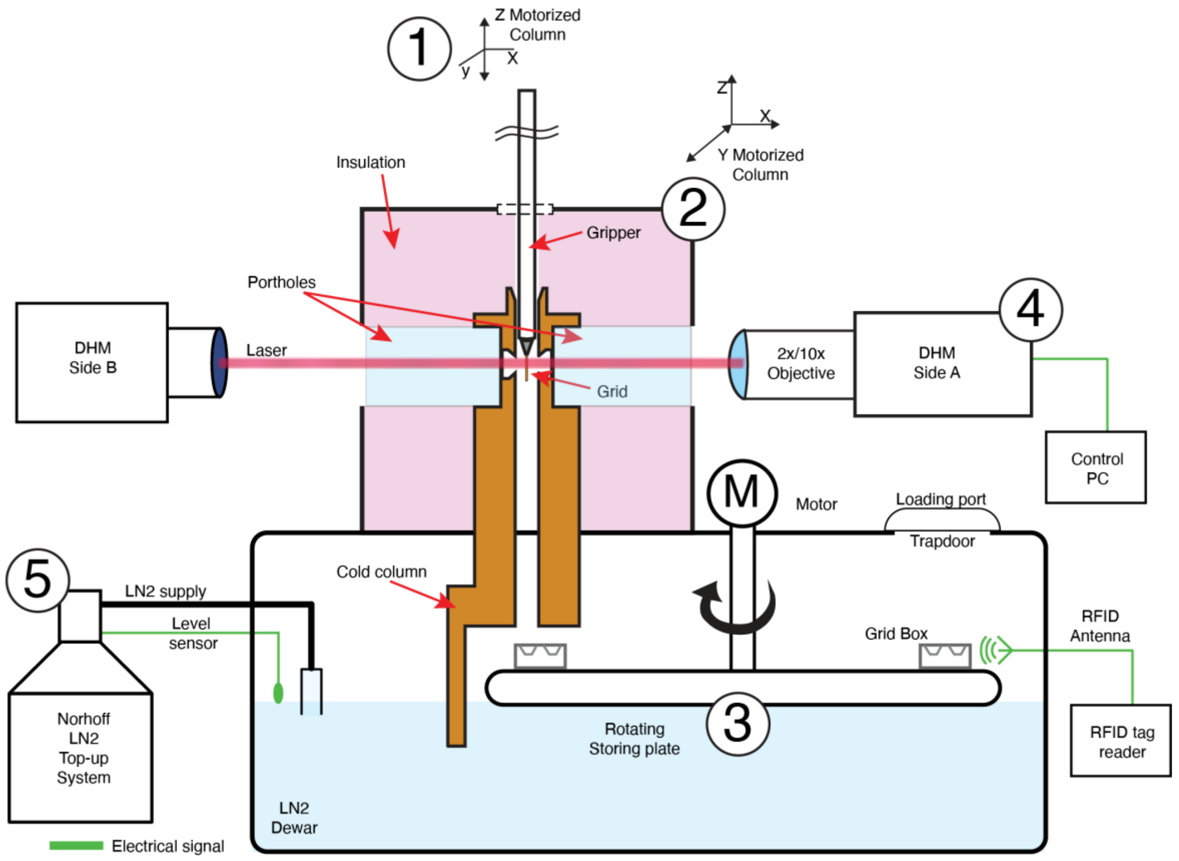

Fig. SN4. Schematics of the EasyGrid Control machine

Simplified schematic diagram of EasyGrid Control with components numbered as follows: (1) Motorized gripper, (2) Motorized observation column, (3) Cryo-storage carrousel, (4) Digital Holographic Microscope, (5) LN_2 regulation system.

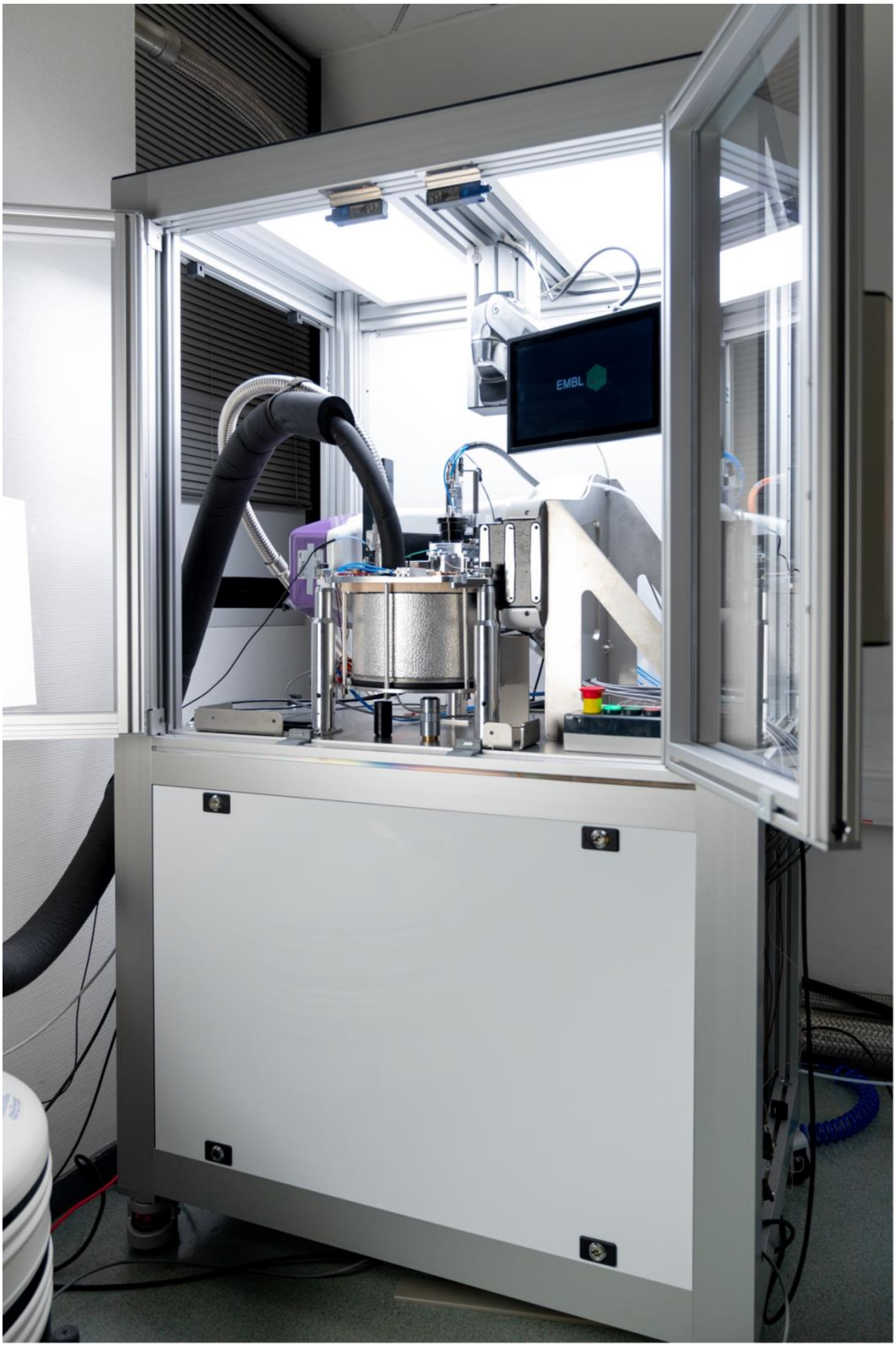

Fig. SN5. Photograph of the EasyGrid Control machine

Graphical user interface (GUI)

The GUI of EGC (Suppl. Note Fig. SN6) allows interacting with the machine using a simple, user-friendly interface. The main components of the GUI are the following:

1. Status panel

This panel displays the overall state of the machine and permits executing the initialization procedures of each module of EGC – or all at once using the “Full Machine Start” button.

2. Session management panel

This panel allows creating or loading a registration file which keeps track of imaged grids and data processing plans.

3. Manual measurement panel

This panel permits manually carrying out measurements on the currently loaded grid.

4. Intensity map

This panel displays an intensity image computed from the collected hologram.

5. Thickness map

This panel displays an thickness map computed from the collected hologram.

6. Advanced actions panel

This panel allows launching low-level, advanced actions, e.g.: starting the DHM live view, focusing the DHM, running scripts, etc.

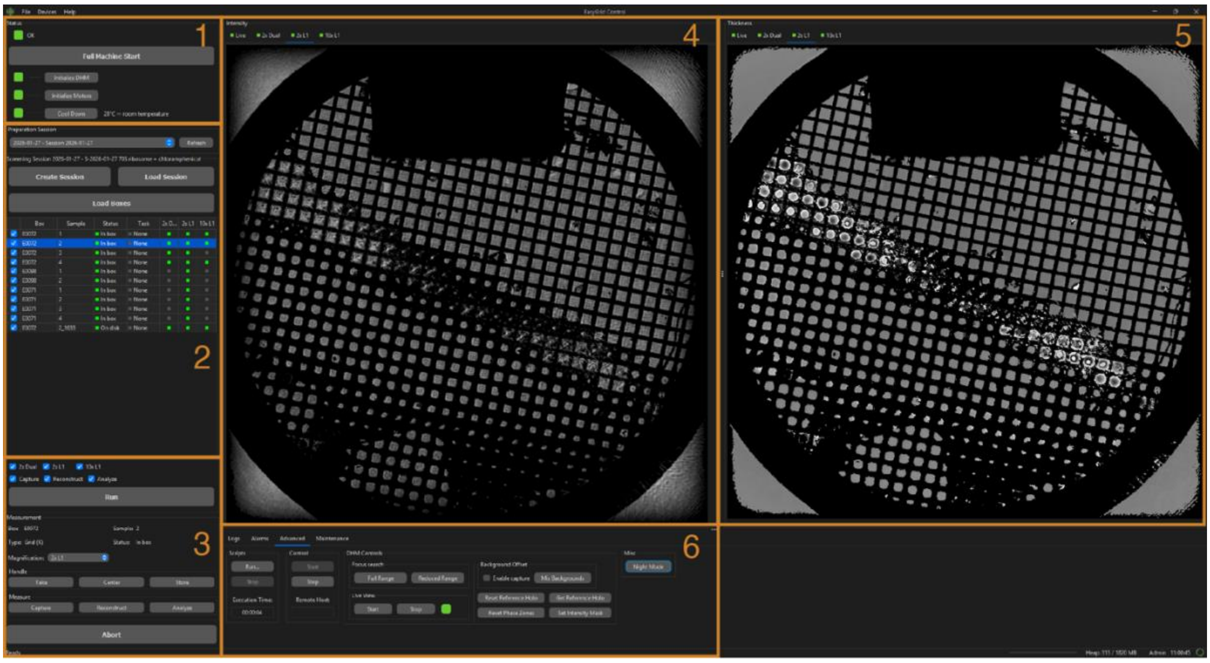

Fig. SN6. Graphical User Interface of the EasyGrid Control machine

Screenshot of the EasyGrid Control GUI with individual panels framed and numbered in orange as follows: (1) Status and high-level commands of the machine, (2) Session panel, (3) Manual measurement panel, (4) Intensity map of the imaged sample, (5) Thickness map of the imaged sample, (6) Advanced actions panel.

EGC includes a standalone grid analyzer tool (Suppl. Note Fig. SN7) to leverage grid measurements from EGC. The grid analyzer allows browsing squares and selecting them according to various criteria. The grid analyzer GUI components are the following:

1. Thickness map parameters.

These controls can modify the rendering of the grid thickness map.

2. Square selection parameters

These controls allow the user to select squares according to various criteria like thickness, distance from center, etc.

3. Grid thickness map

This panel displays the grid thickness map, with overlays highlighting the squares matching selected criteria.

4. Grid histogram
This histogram displays the distribution of squares in the selected thickness range.

5. Selected square measurements
This panel shows the measurement statistics for a single square, along with a close-up image and a histogram displaying the distribution of pixels in the selected thickness range.

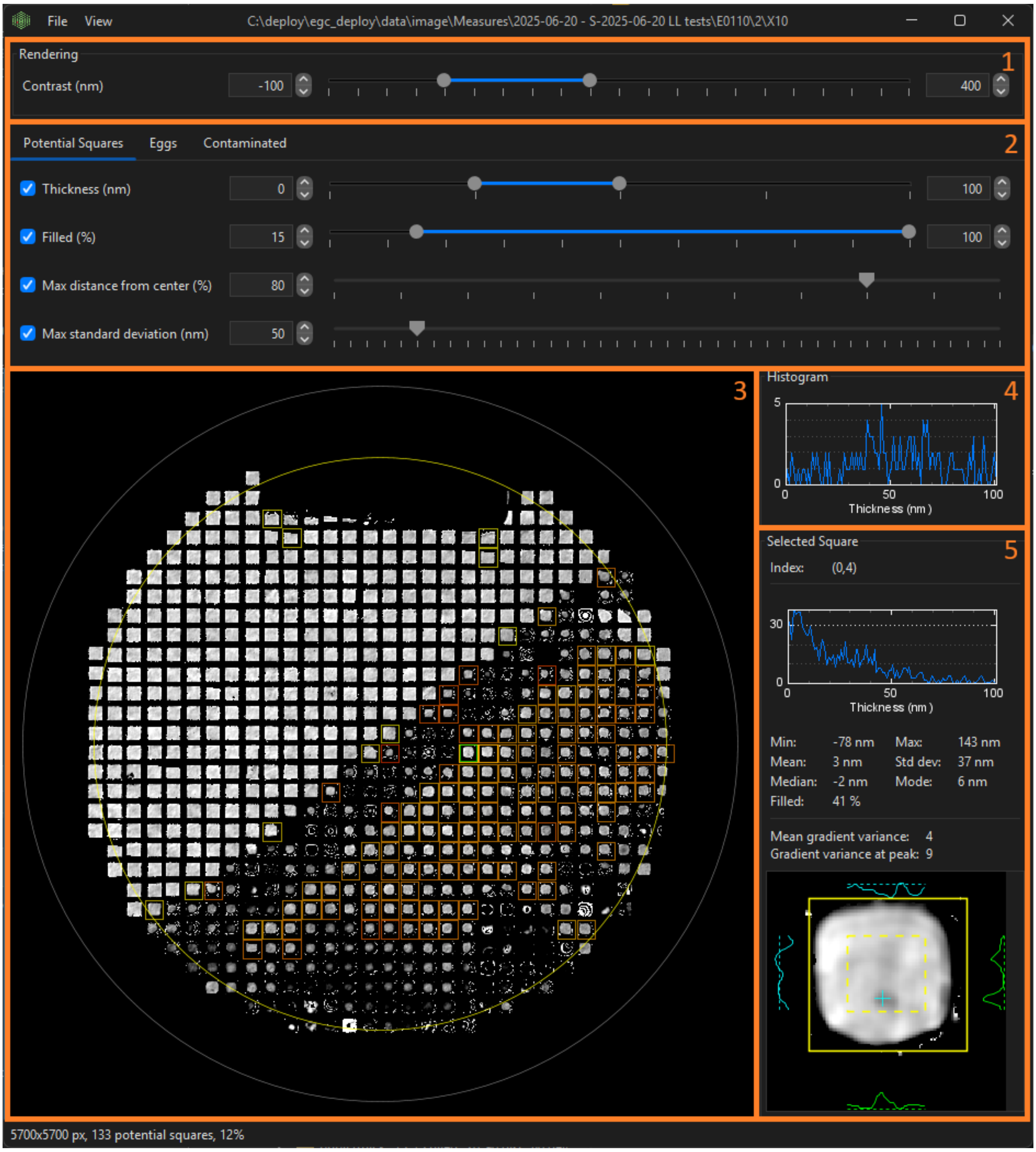

Fig. SN7. EasyGrid Control grid analyzer GUI

Screenshot of the EasyGrid Control grid analyzer GUI with individual panels framed and numbered in orange as follows: (1) Thickness map parameters, (2) Square selection parameters, (3) Grid thickness map, (4) Grid histogram, (5) Selected square measurements.

Image acquisition and processing steps

We have developed an algorithm allowing sample thickness mapping in EGC (Suppl. Note Fig. SN8). This process combines DHM imaging at two magnification levels (2x and 10x) to benefit from 10x magnification despite the small field of view (FOV) achievable with the 10x objective (~600 µm x 600 µm). Sample holograms acquired with this small FOV size usually lack an internal thickness reference required to convert the phase information derived from collected holograms into sample thickness estimations. By contrast, the FOV achievable with the 2x magnification objective (~3 mm x 3 mm) enables reference measurements in the empty “0 nm thickness” regions bordering the sample. The 2x hologram of a given sample is therefore useful to calibrate and interpret a tiled hologram of the same sample acquired at 10x magnification. Based on this principle, the EGC procedure for generating and calibrating 10x sample thickness maps is the following:

1. Image acquisition at 2x and 10x magnification

Before imaging grids with the DHM, a hologram of the background is recorded with no sample in the FOV (reference hologram; Suppl. Note Fig. SN9 a). The sample is then inserted in the FOV and a hologram of the entire grid is recorded using the 2x objective (Suppl. Note Fig. SN9 b). The 2x objective is then swapped with a 10x objective to acquire a tiled hologram of the grid (Suppl. Note Fig. SN9 c) in a 9x9 grid configuration. Because tiled acquisition requires moving the cryo-observation column horizontally, which modifies the background (and moving the grid vertically, which doesn’t), 10x references are acquired for each position of the cryo-column.

2. Pre-processing 2x data

The software development kit (SDK) provided with the DHM enables processing acquired holograms. First, the reference hologram is loaded to subtract the background signal from the sample hologram. Then, grid edges are detected, the position of the center of the grid and its radius are measured, and sub-regions employed for phase correction and phase offset adjustment during reconstruction are automatedly delineated in the corners of the image, outside of the grid. Optimal focus, also referred to as the reconstruction distance, is determined at grid edges in each corner of the image using a gradient of variance analysis. The image is rasterized into 9x9 sub-tiles and optimal focus is interpolated throughout the image to compensate for grid bending. A series of 9x9 intensity and phase images are then reconstructed at optimal foci using the sub-routine outlined in Suppl. Note Fig. SN8: the intensity image is first reconstructed and automatedly thresholded to produce a mask removing signal on grid bars from the output phase image. The hologram is then low-pass filtered (cut-off of 10 pixels) to mitigate aliasing artifacts caused by the grid squares because their size is close to the resolving limit of the optical system at 2x magnification. The 9x9 intensity or phase images are finally merged into composite 2x maps wherein the entire grid is in focus (Suppl. Note Fig. SN9 d&e). Resulting intensity maps resemble standard light microscopy images but phase maps, also called optical thickness maps, contain valuable information about local sample thickness.

3. Reference grid image subtraction

It has been observed that inserting even an empty grid support into the observation column significantly deforms the measured thickness signal compared to background measurements performed in the absence of any specimen. This effect was found to be reproducible across multiple experiments conducted over several months.

To eliminate signal contributions from the sample support and ensure that only the vitrified sample layer atop the support is measured, a reference grid image calibration step was implemented. This process involves acquiring images of empty sample holder grids at various orientations and positions within the observation column and merging them into a single reference grid background image while discarding grid areas blocking the DHM beam. The resulting reference image is subsequently processed using a median filter (kernel radius of 19) followed by a gaussian blur (sigma of 20) to smoothen its overall aspect.

The reference grid image is constructed once and remains valid for extended periods, provided no major modifications occur within the optical path of the DHM or the cryo-observation column. In the final step, this reference image is subtracted from images obtained in step 2. Consequently, when measuring empty Quantifoil grids, EasyGrid Control (EGC) typically reports an average thickness of 0 nm within intact grid squares, while broken squares yield negative thickness values. This methodology exposes the measurements to potential fluctuations in carbon foil thickness, which manifest as systematic offsets in the recorded data (further details and experimental data is available in the performance assessment section below).

4. Post-processing 2x data

Grid thickness measurements are referenced against the phase signal obtained from the background region surrounding the grid. However, to eliminate contributions from the carbon support film of the sample holder grid (that often displays slightly varying film thickness), a baseline offset may be required to ensure that a measured thickness of 0 nm corresponds to truly empty grid squares. Empty squares are identified based on two criteria: an initial thickness below a defined threshold and a characteristic square-shaped contour, as opposed to ice-filled squares, which exhibit higher thickness and curved boundaries. Once a sufficient number of empty squares are identified, a global offset is applied to the thickness measurements across the entire grid, such that these empty squares are normalized to a thickness of 0 nm.

5. Pre-processing 10x data

The DHM SDK is also used to process tiled 10x holograms. Initially, phase images of all tiles are reconstructed with default settings and stitched together to determine the position of the center of the grid and its radius. Similarly, to 2x hologram processing, optimal focus is determined at the grid edges, followed by focus interpolation and reconstruction of phase images for all tiles in relevant focus. Here, holograms are low-pass filtered with a cut-off of 20 pixels to mitigate aliasing artifacts caused by the grid holes because their size is close to the resolving limit of the optical system at 10x magnification. Reconstructions are finally merged to produce composite 10x maps wherein the entire grid is in focus (Suppl. Note Fig. SN9 f&g).

6. Reconstruction of calibrated thickness map at 10x magnification.

After pre-processing, 2x and 10x maps are combined to calibrate the sample thickness readouts in 10x tiles. Each tile position is first matched with their expected position in the 2x phase image. Individual squares are detected in each tile and square positions are registered. In the case of single squares appearing in multiple tiles due to image overlap, square instances with the largest consistent areas are saved and redundant squares are discarded. Data from each square in 10x tile are then normalized to the phase values recorded in the corresponding regions of the 2x map i.e., applying an offset to compensate the difference between the measured values in 2x and 10x. Finally, an affine transformation is applied to each square to produce a 10x map with minimal amounts of stitching artifacts. We refer to this map as sample thickness map (Suppl. Note Fig. SN10).

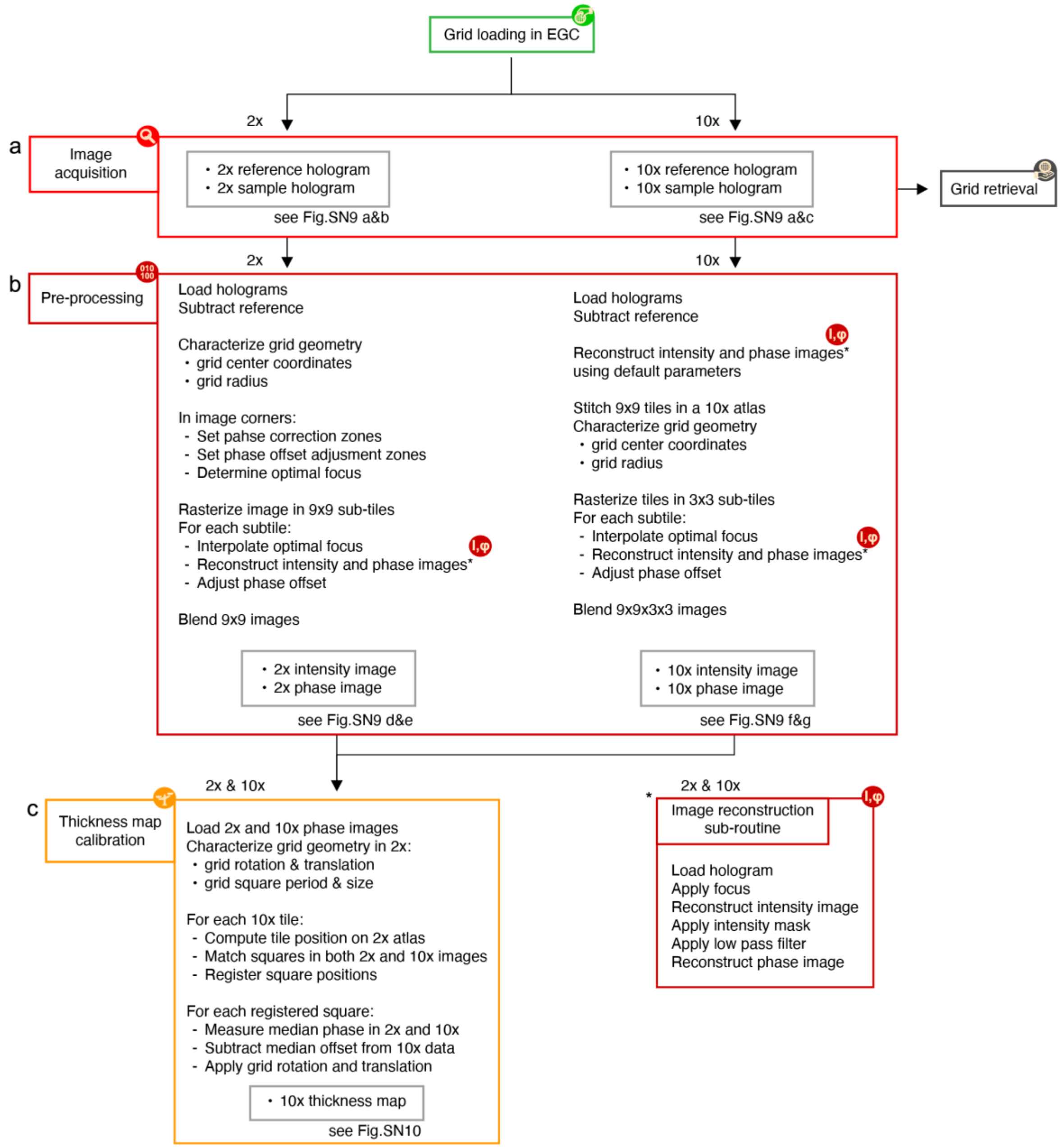

Fig. SN8. EGC image acquisition and processing scheme

EGC grid mapping scheme, from grid loading and hologram acquisition at 2x and 10x magnification (a) to pre-processing (b) and the combination of 2x and 10x data into a calibrated 10x sample thickness map (c).

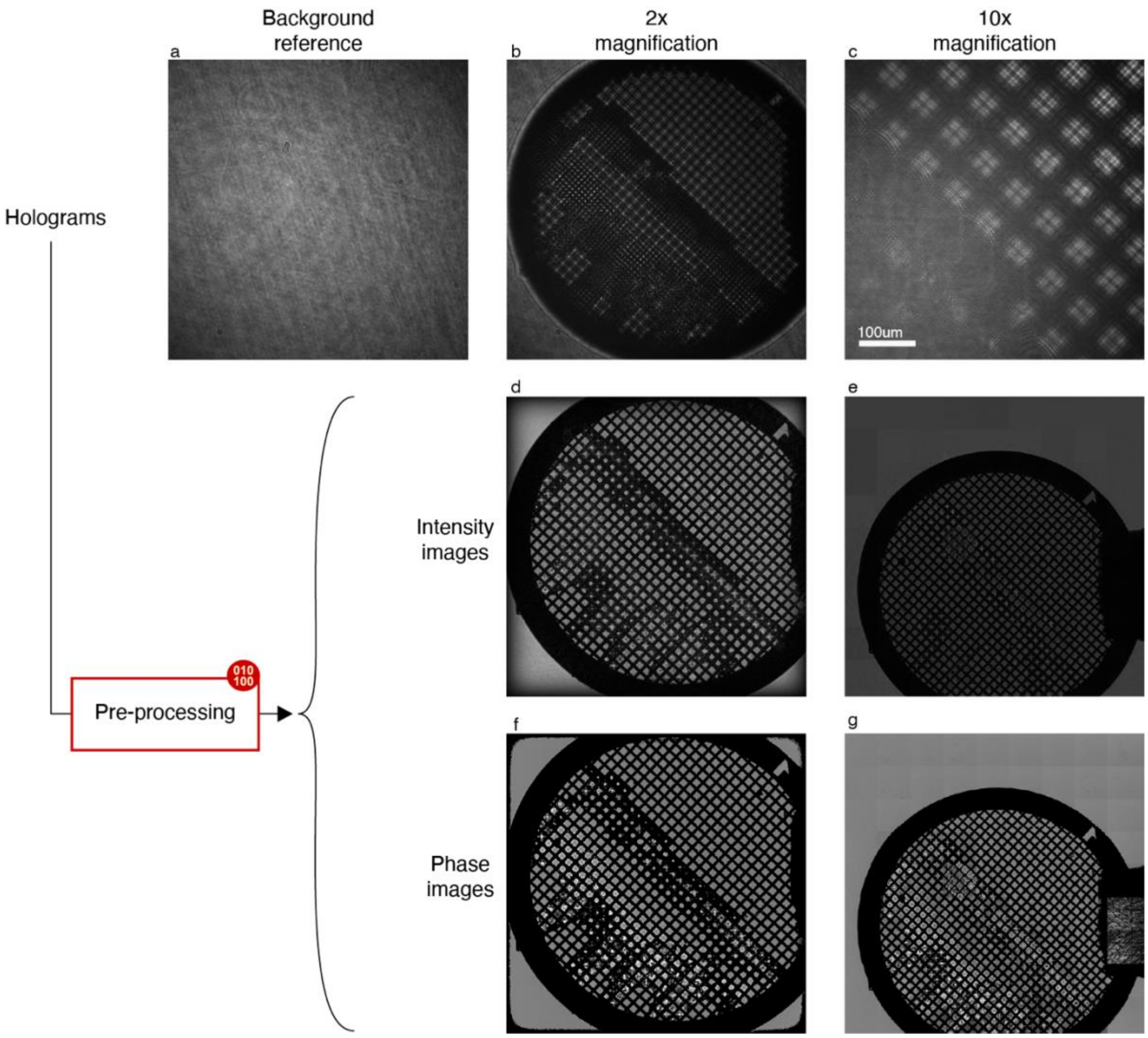

Fig. SN9. Example images produced with EGC at intermediate processing steps

a. Reference hologram with no sample in the field of view.

b. 2D projection of a hologram of a grid prepared with EasyGrid for single-particle analysis, acquired at 2x.

c. 2D projection of a hologram of one tile of this grid, imaged at 10x magnification. No thickness reference can be found in this tile; scale bar, 100 µm.

d. 2x intensity image reconstructed from the hologram in b.

e. 2x phase image reconstructed from the hologram in b. Note the gradient of thin ice in the central region of the grid.

f. 10x intensity image reconstructed from 9x9 tiled holograms similar to the one in c.

g. 10x phase image reconstructed from 9x9 tiled holograms similar to the one in c. Note how background values vary in each tile due to the absence of a thickness reference. This can be corrected by normalizing tiles using information from the 2x map displayed in e.

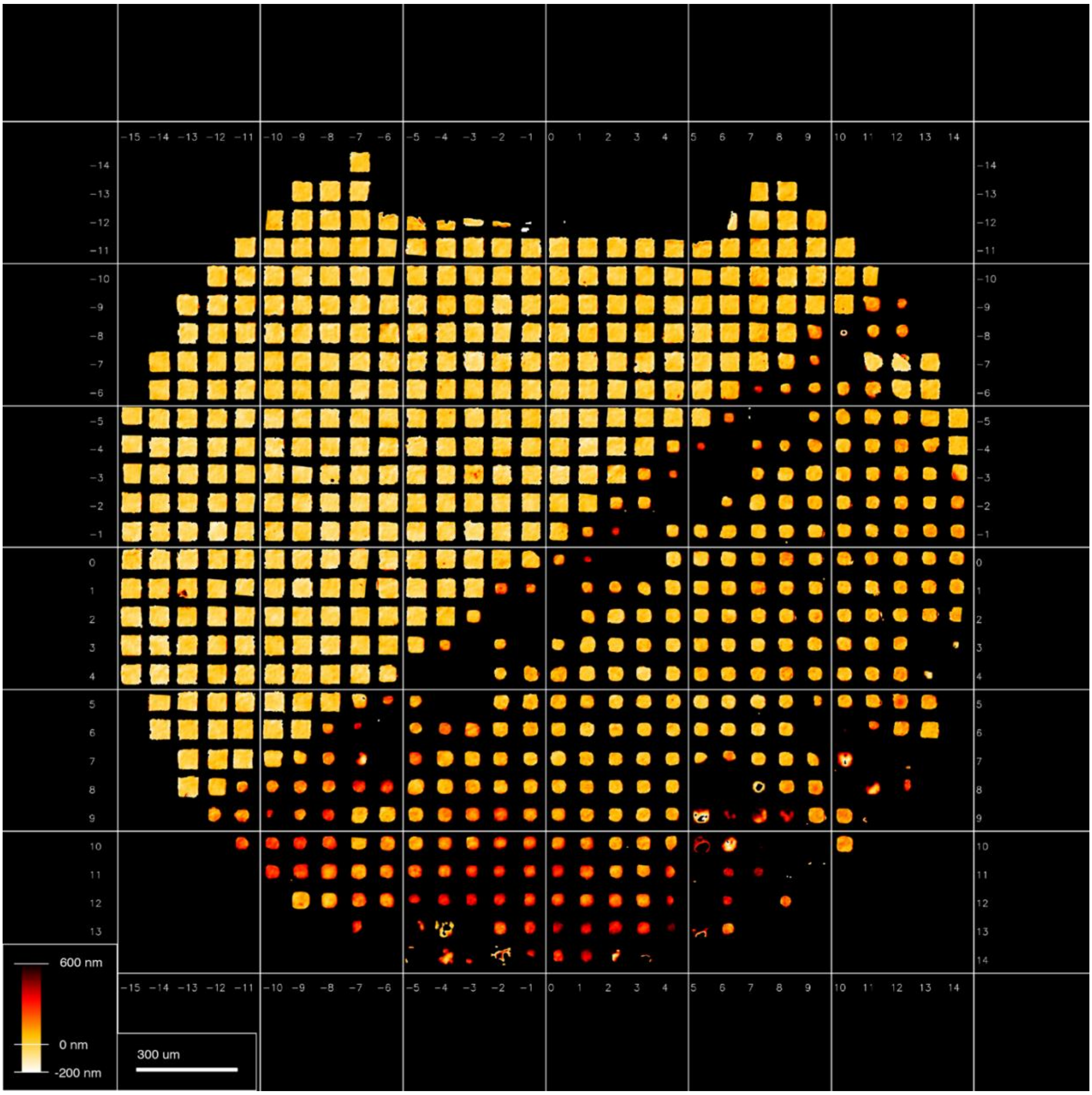

Fig. SN10. Calibrated 10x thickness map of a cryo-EM grid prepared with EasyGrid

EGC thickness map produced following the pipeline described in Fig. SN8. Note that the central region of the grid (columns 0 to 4, row 5 to 9 and columns 5 to 9, rows 0 to 4) displays promisingly thin ice for cryo-EM imaging and single-particle analysis.

Performance assessment

To evaluate the performance of our cryogenic sample thickness measurement system, two supplementary experiments were conducted. In the first experiment, empty cryo-electron microscopy sample support grids were measured to establish baseline reproducibility and to quantify the associated error margin. In the second experiment, comparative thickness measurements were performed on the same sample grid using EasyGrid Control and cryo-electron tomography.

Measurement of empty sample holder grids

We analyzed four Quantifoil R2/2 and four Quantifoil R1.2/1.3 cryo-EM sample grids, imaging them using the EGC machine according to the previously described 2x prescreening protocol. The grids were directly transferred from their original box to the EGC machine. The results for both sample batches were highly consistent (Figure SN11). The histograms of the median square thickness measurements revealed an average standard deviation of 17nm nm.

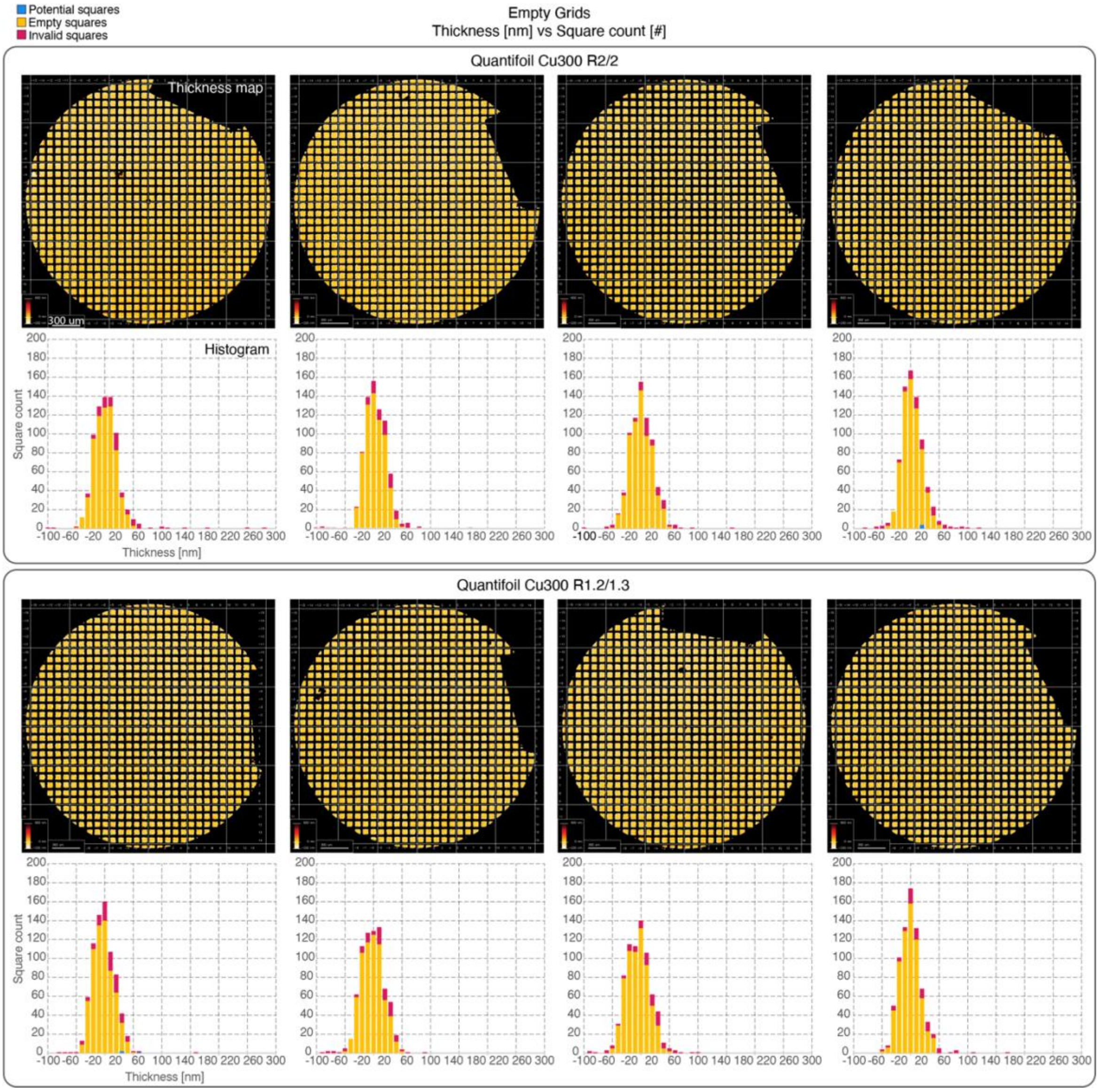

Fig. SN11. Measurement of empty Quantifoil grids.

Grid histograms generated by EGC categorize squares in 3 categories: (1) Empty squares (yellow) i.e., thickness mode between -50 nm and 50 nm, thickness standard deviation below 50 nm, filled between 0% and 25%,distance to the grid center below 80% of the grid radius; (2) Potential (green) i.e., thickness mode between 15 nm and 75 nm, thickness standard deviation below 50 nm, filled between 20% and 90%,distance to the grid center below 80% of the grid radius;
(3) Invalid (red), neither empty nor potential square.

Comparative measurement between EasyGrid Control and Cryo-Electron Tomography

Mouse Apoferritin was obtained from Protein Expression and Purification Core Facility at EMBL Heidelberg and used for cryoEM grids preparation at the concentration 4.4 mg/ml. The sample was clarified by centrifugation at 18213 rcf for 10 minutes and 3 µL was applied to Quantifoil R2/2 Copper 200 mesh grids glow discharged for 1 min at Fischione 1070 Plasma Cleaner in gas mixture of 20% of oxygen in argon. The sample was mounted on at the Vitrobot Mark IV (ThermoFischer Scientific) at 100% humidity and 4°C, blotted for 4 sec at 0 blotting force and plunge-frozen in liquid ethane.

The sample grid was transferred to the EasyGrid Control instrument for ice thickness mapping at 2x magnification. Four squares displaying progressively increasing ice thickness have been selected for further ice thickness determination using Cryo-Electron Tomography.

The subsequent data was collected on a 200 kV Glacios microscope equipped with a Falcon 4i camera operating in counting mode and a SelectrisX energy filer set to 10 eV, with Tomography software v5.17 (ThermoFisher Scientific). Tilt series were acquired at 4 different squares. A dose-symmetric scheme was applied (total dose 120 e-/A^2), using angles ranging from -60° to +60° with a 3° tilt increment, and performed at a 79,000x magnification, corresponding to a pixel size of 1.55 Å. The defocus was comprised between -0.5 and -3 mm using a -0.25 µm increment.
The motion and CTF corrections of the tilt series were performed using WARP 1.0.9, before to be aligned using AreTomo 1.3.4 and reconstructed using WARP 1.0.9. The pixels of tomograms were bined to 12.4 Å.

The tomograms were segmented into 35 slices through the sagittal plane using ChimeraX-1.7.1. The gray values of the 35 slices were summed and plotted as a function of the distance in pixels using ImageJ2 2.14.0/1.54f. The thickness of the ice layer was determined by measuring the peak width(s).

As shown in Fig. SN12, the error bars for the thickness measurements obtained using EGC exhibit noticeable overlap with those from the corresponding cryo-ET values. This indicates that EGC provides reliable ice thickness measurements at cryogenic temperatures.

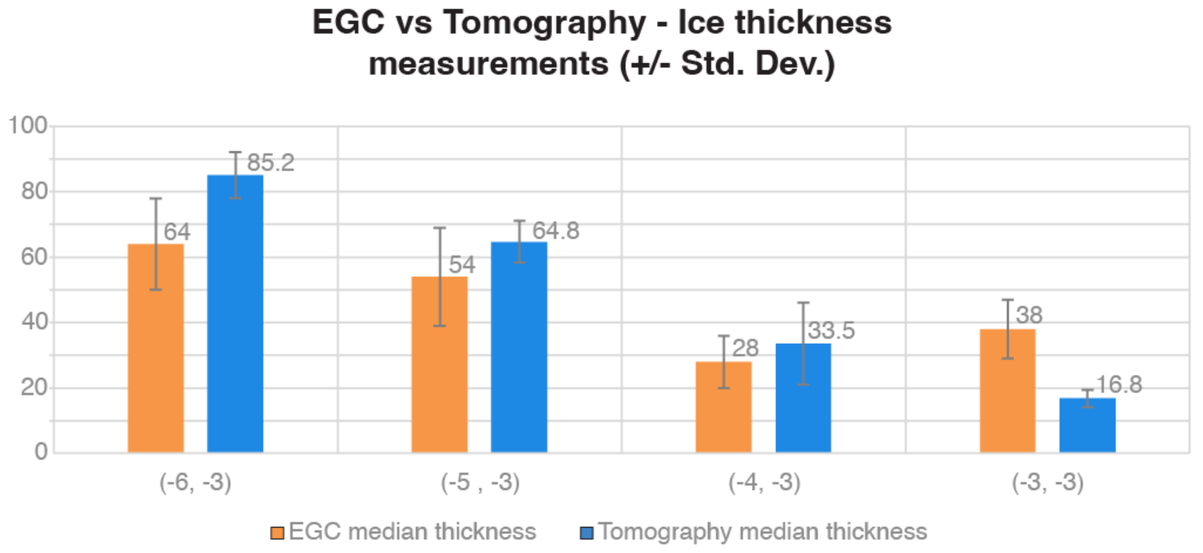

Fig. SN12. Comparative ice thickness measurement with EasyGrid Control and Cryo-ET.

The grid squares are identified by their XY coordinates from the EGC thickness map (not shown) i.e., (-6, -3), (-5, -3), (-4, -3), (-3, -3).

Note S2: Additional information on pressure wave based spreading system behavior

Effect of pressure wave parameters

The pressure wave-based sample spreading system represents a novel approach in the field of cryo-EM sample preparation. Consequently, a systematic investigation was required to elucidate the influence of various pressure wave parameters on vitrified grid's ice thickness profiles. Due to the inherent variability of purified protein samples and their unpredictable behavior on sample support grids, milliQ water was used for this study. We must note that results obtained within this study cannot be generalized for all purified protein sample solutions, and dedicated optimization for de-novo samples must be carried out following the optimization protocol described in Suppl. Note S3.
In accordance with the standard preparation protocol, all sample holder grids underwent 5 cycles of plasma treatment using the EasyGrid system. Subsequently, half of the surface of each grid was coated with Blue Dextran (BD) (Merck; ref. D4772) by dispensing 100 droplets of 2 mg/mL BD in a horizontal line at the central axis. The BD spreading process was performed under conditions of >80% humidity at 4 °C using a 2.0-bar pressure wave for a duration of 30 ms, followed by a 10-second drying period at ambient humidity and temperature.
To assess the repeatability of the system, three sample grids were systematically prepared for each experimental condition. These grids were subsequently transferred to the EGC for the generation of sample thickness maps and histograms depicting the average square thickness distribution.

Effect of air pressure

The EG instrument is equipped with an electronically controlled manometer, enabling on-the-fly adjustment of the supply air pressure within the pressure wave generator. To elucidate the influence of air pressure on the sample spreading mechanism, four spreading experiments with triplicates were conducted at progressively increasing air pressures, ranging from 1.2 bar to 1.8 bar, on BD-coated Quantifoil R2/2 grids using Milli-Q water. All other preparation parameters were maintained constant throughout the experiments, including a pressure wave duration of 80 ms, no post-spreading waiting time, and a total of 100 dispensed drops.
The findings (Figure SN13) demonstrate a subtle influence of air pressure on the sample spreading mechanism and a high similarity between grids prepared under the same conditions. The average number of usable grid squares exhibiting the ideal ice thicknesses (within the 15 nm to 75 nm range) were recorded, together with the number of empty and invalid squares at 1.2 bar, 1.55 bar, and 1.8 bar.
In conclusion, a clear trend is observed: increasing air pressures leads to thinner sample layers and larger spread areas. However, highest pressures displace more sample, reducing the number of potentially usable squares. The optimal ice thickness was consistently achieved at 1.55 bar.

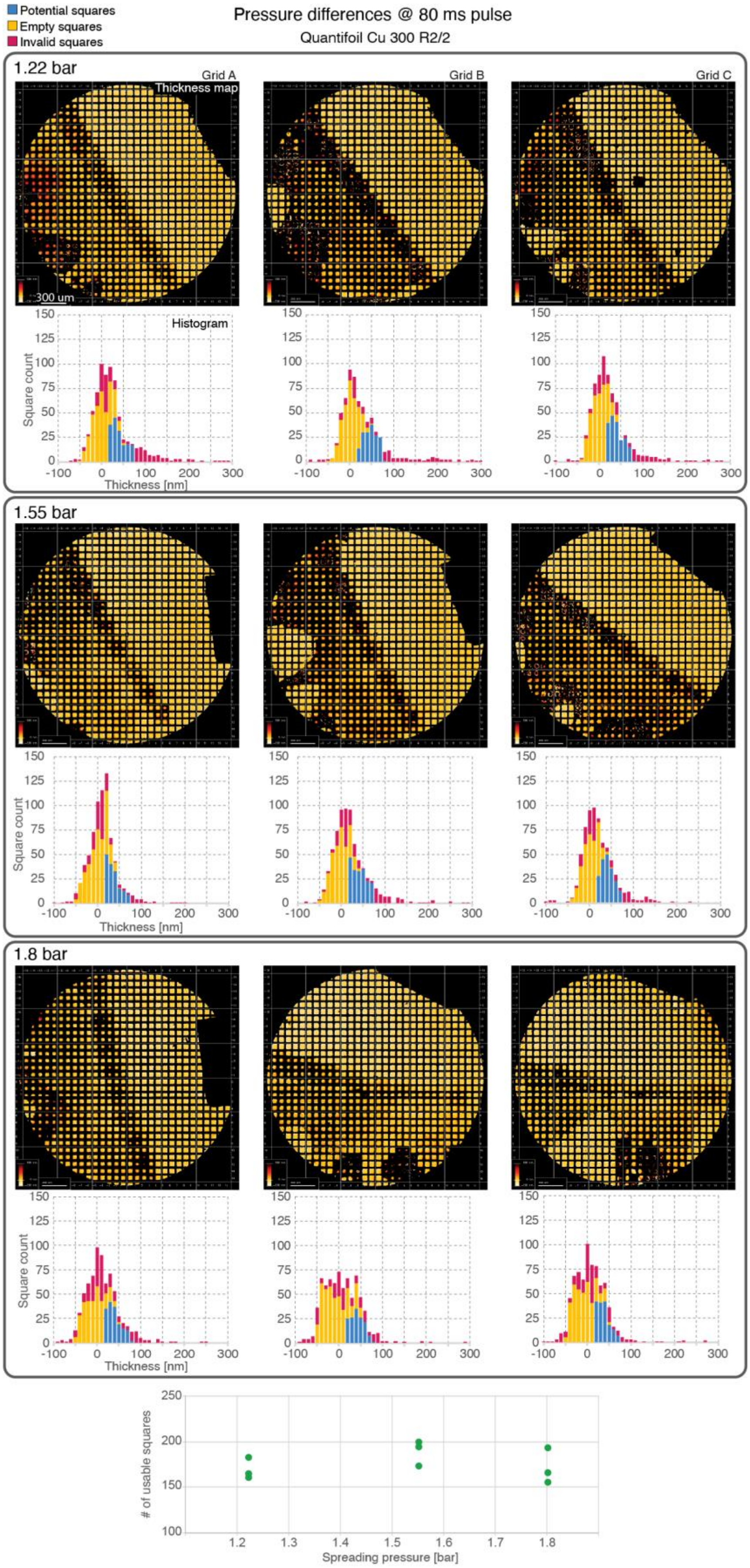

Fig. SN13: Effect of air pressure on sample spreading

Effect of pulse duration

To highlight the influence of pressure wave duration on the sample spreading, we applied Milli-Q water onto BD-coated Quantifoil Cu 300 R2/2 grids and conducted four spreading experiments in triplicates. The pulse durations were progressively increased from 10 ms to 120 ms. All other preparation parameters were maintained constant throughout the experiments, including the total number of dispensed drops (100) and the air pressure that was maintained at 1.55 bar. We did not use post-spreading waiting time.

The outcomes of these experiments (Figure SN14) demonstrate a significant influence of pressure wave duration on the sample spreading mechanism, along with a high similarity between grids prepared under the same conditions. The average number of grid squares exhibiting ice thickness within the 15 nm to 75 nm range were recorded as potentially usable along with counts of empty and invalid squares for a 10 ms, 50 ms, 80 ms, and 120 ms pulse duration.

In conclusion, a trend is observed: at shorter durations, the sample spreading is minimal. As the duration increases, spreading improves progressively reaching a maximum at 80ms. Beyond this point, the reproducibility is impaired and sample spreading area reaches a plateau. This may be attributed to the onset of turbulent flow conditions within the preparation chamber for longer pulse durations. An optimal ice thickness was consistently achieved at 80 ms.

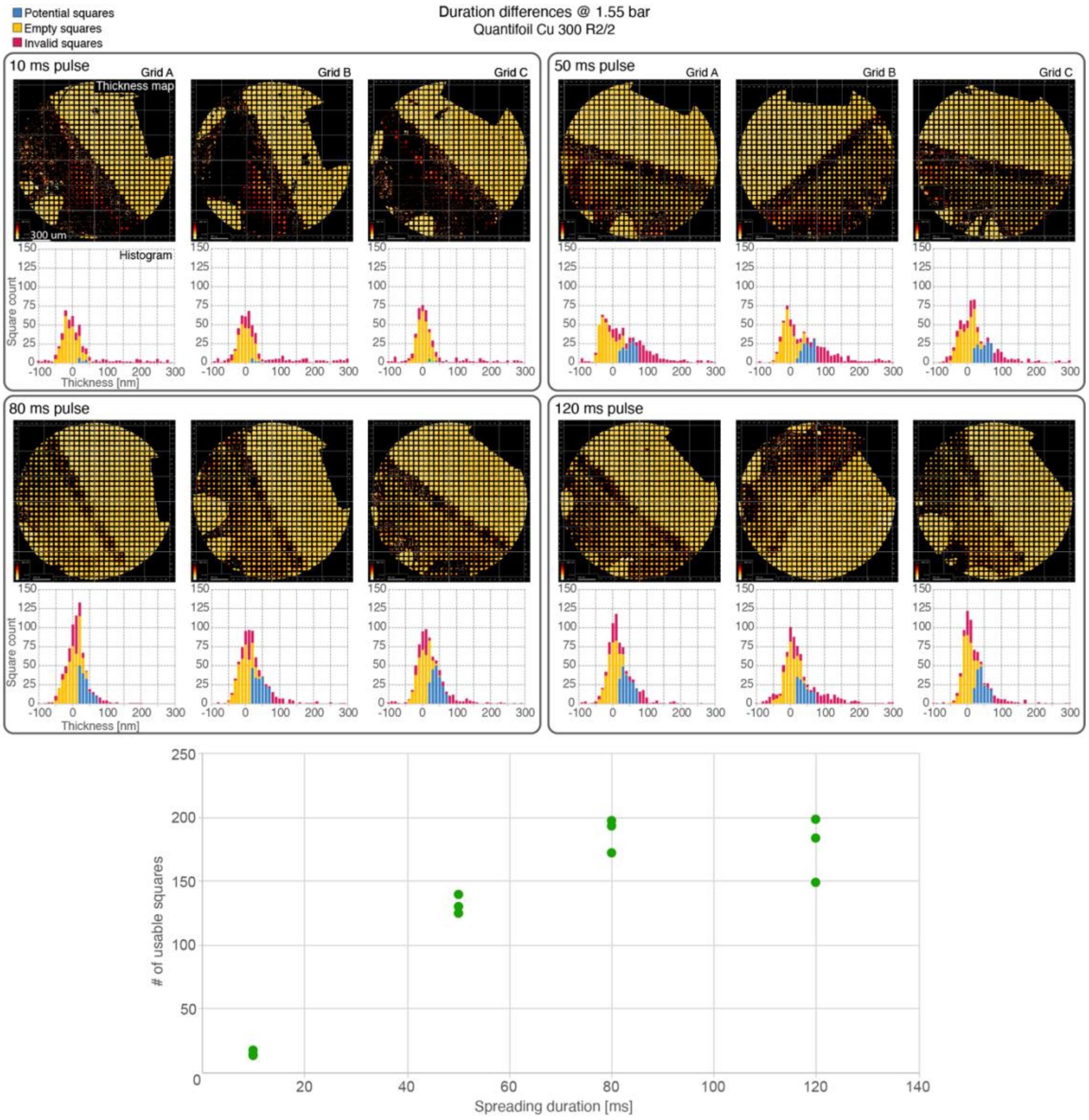

Fig. SN14: Effect of pressure wave pulse duration on sample spreading

Assessement of repeatibility using protein samples

To assess the repeatability of the sample preparation process not only with Milli-Q water (as in the previous section) but with a protein sample, we used a guanidinase solution concentrated at 1.4 mg/mL in a buffer containing 150 mM NaCl and 25 mM HepesKOH pH 7.5. We conducted three sample experiments, each with a distinct set of preparation parameters, and subsequently screened them at the EGC and on our Glacios cryo-electron microscope. Each experiment was performed in triplicate to highlight the reproducibility of our workflow.
The sample spreading parameters for the three experiments were as follows: (1) 30 ms pulse duration, no post-spreading delay; (2) 50 ms pulse duration, no post-spreading delay; (3) 50 ms pulse duration, 200 ms post-spreading delay. All preparations were performed on BD-coated Quantifoil Cu 300 R2/2 grids. Other preparation parameters were kept constant across all experiments, including the total number of dispensed drops (100) and the air pressure (1.55 bar).
The results of this experiment (Fig. SN15) show high overall grid reproducibility when using the same preparation parameter set. They also confirm that longer pulse durations result in thinner ice, and further show that a 200 ms post-spreading delay enables controlled and reproducible post-spreading evaporation, allowing fine-tuning of sample thinning.

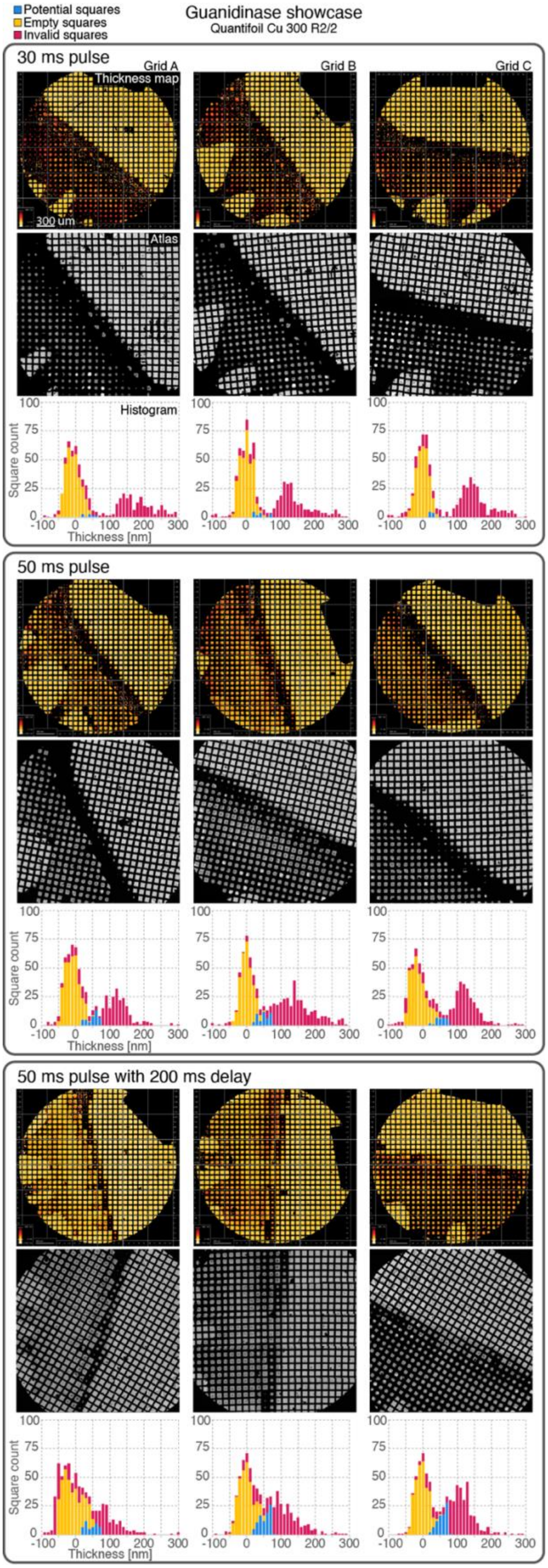

Fig. SN15: Reproducibility essays using Guanidinase protein solution

Note S3. Preparation parameters optimization protocol

To address the highly variable behavior of different biological solutions on the surface of sample holder grids, we have developed a standardized parameter optimization protocol for SPA sample preparation using EasyGrid instruments (Figure SN15). This protocol incorporates three iterative feedback loops: (i) imaging via the camera integrated within the EasyGrid preparation chamber, (ii) ice thickness mapping performed using the EGC instrument, and (iii) Cryo-EM screening and data collection. De novo samples are initially evaluated for their interaction with the support grids during the spreading process, which is facilitated by pressure waves. In this initial assessment, we employ the default sample preparation parameter set:

Number of drops	100
Number of dispensing spots	10
Coating	1 layer of BD
Pulse number	1
Pulse duration	80 ms
Supply pressure	1.55 bar
Grid type	R2/2
Post spreading waiting time	0 ms

Table SN 1: default preparation parameters

An image of the prepared grid is automatically captured at room temperature using the preparation chamber's integrated camera immediately after the spreading process (spreading-to-observation delay ~60 ms). The operator reviews the image and adjusts the preparation parameters until the sample spreading is deemed satisfactory. Key adjustable parameters include the number of pulses, pulse duration, air pressure, coating type and thickness, and grid type.

Once optimal sample spreading is achieved, a small set of grids is vitrified using the same dispensing and spreading parameters, while the post-spreading waiting time is systematically varied, typically from 0 ms to 300 ms. The vitrified grids are then transferred to the EGC instrument for ice thickness mapping. If any grid exhibits satisfactory ice thickness, it is selected for subsequent Cryo-EM data collection. Otherwise, the operator returns to the parameter optimization step. Should the selected grid fail to yield a structure of sufficient resolution, the operator revisits the sample preparation parameters for further refinement.

All preparation parameters and environmental conditions (e.g., chamber temperature and humidity, dispensing pipette temperature) are automatically recorded for each trial by the EasyGrid instrument. This systematic data logging facilitates parameter tracking, enabling efficient optimization of the preparation process.

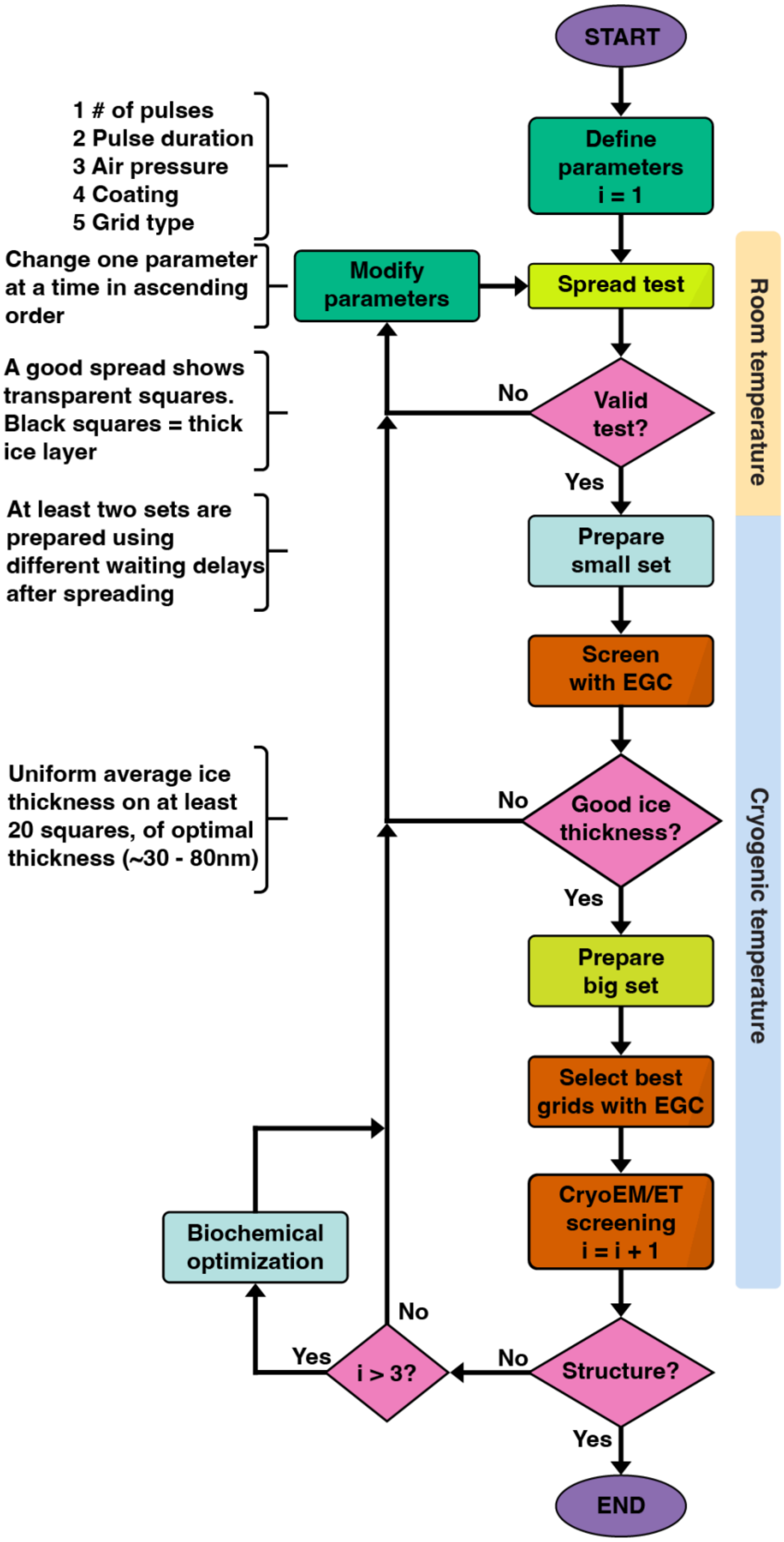

Fig. SN16. Visual representation of the sample optimization protocol used on the EasyGrid instruments
