# Supplementary figures and images for: EasyGrid: a versatile platform for automated cryo-EM sample preparation and quality control
Source: Nat Methods. 2026 Jun 23;23(7):1359–67. doi: 10.1038/s41592-026-03127-5 (PMC13345917; doi:10.1038/s41592-026-03127-5)

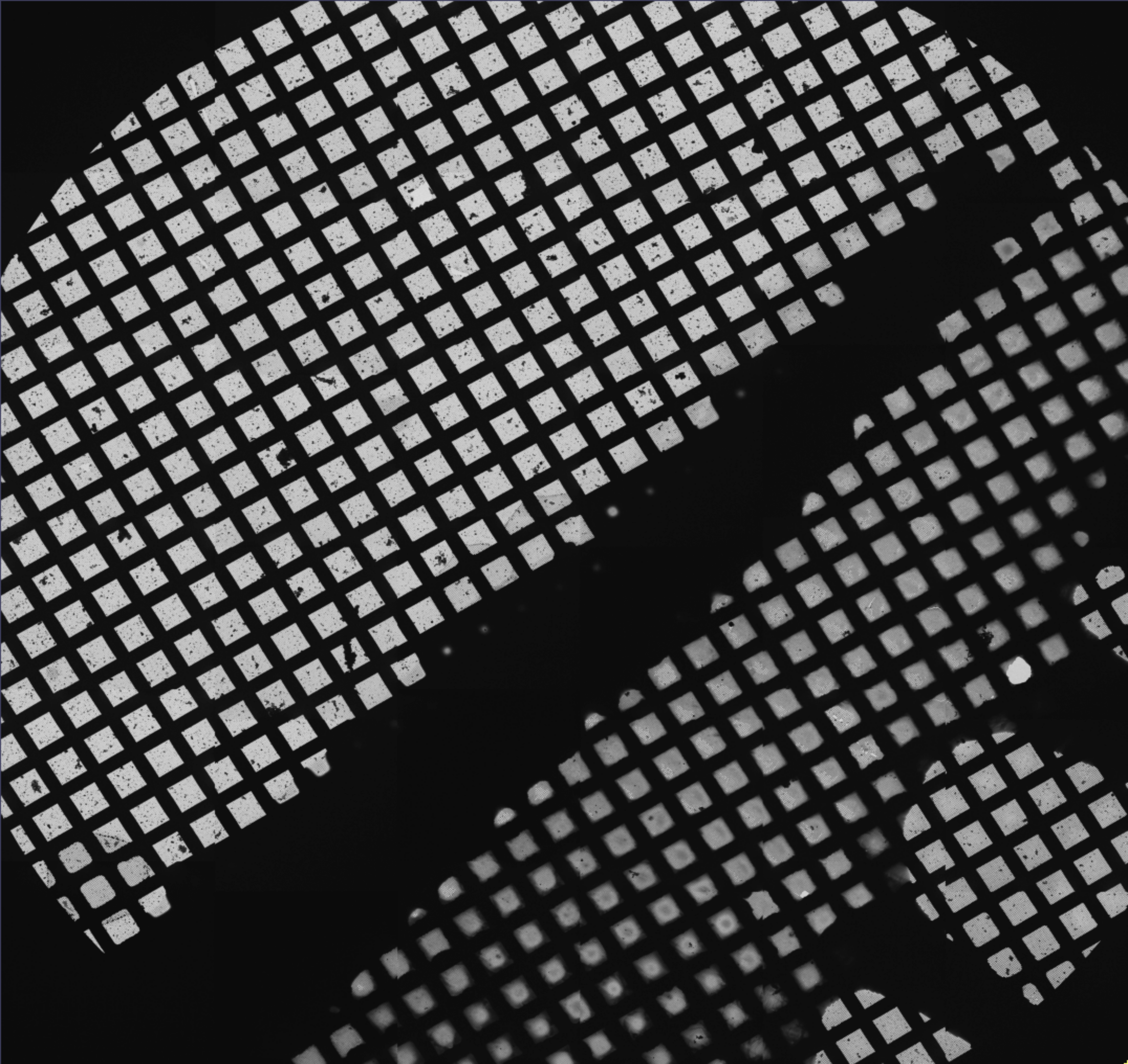

Supplement: Supplementary file 6 — Original images of the figure in high resolution. [file 41592_2026_3127_MOESM6_ESM.zip › Figure3/Fig3_SPApipe_gridAtlas.jpg]

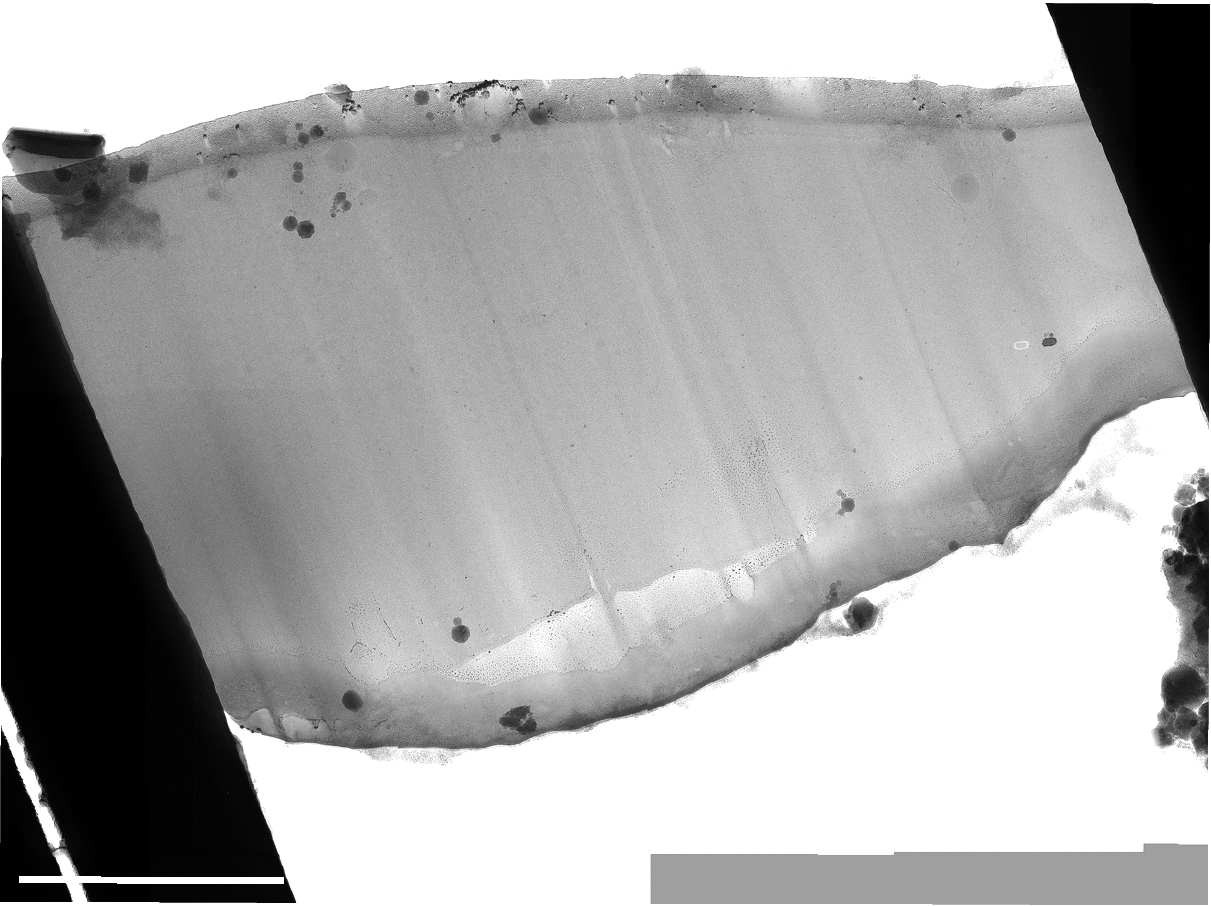

Supplement: Supplementary file 7 — Original images of the figure in high resolution. [file 41592_2026_3127_MOESM7_ESM.zip › Figure4/Fig4_CellsAdh_lamella2.png]

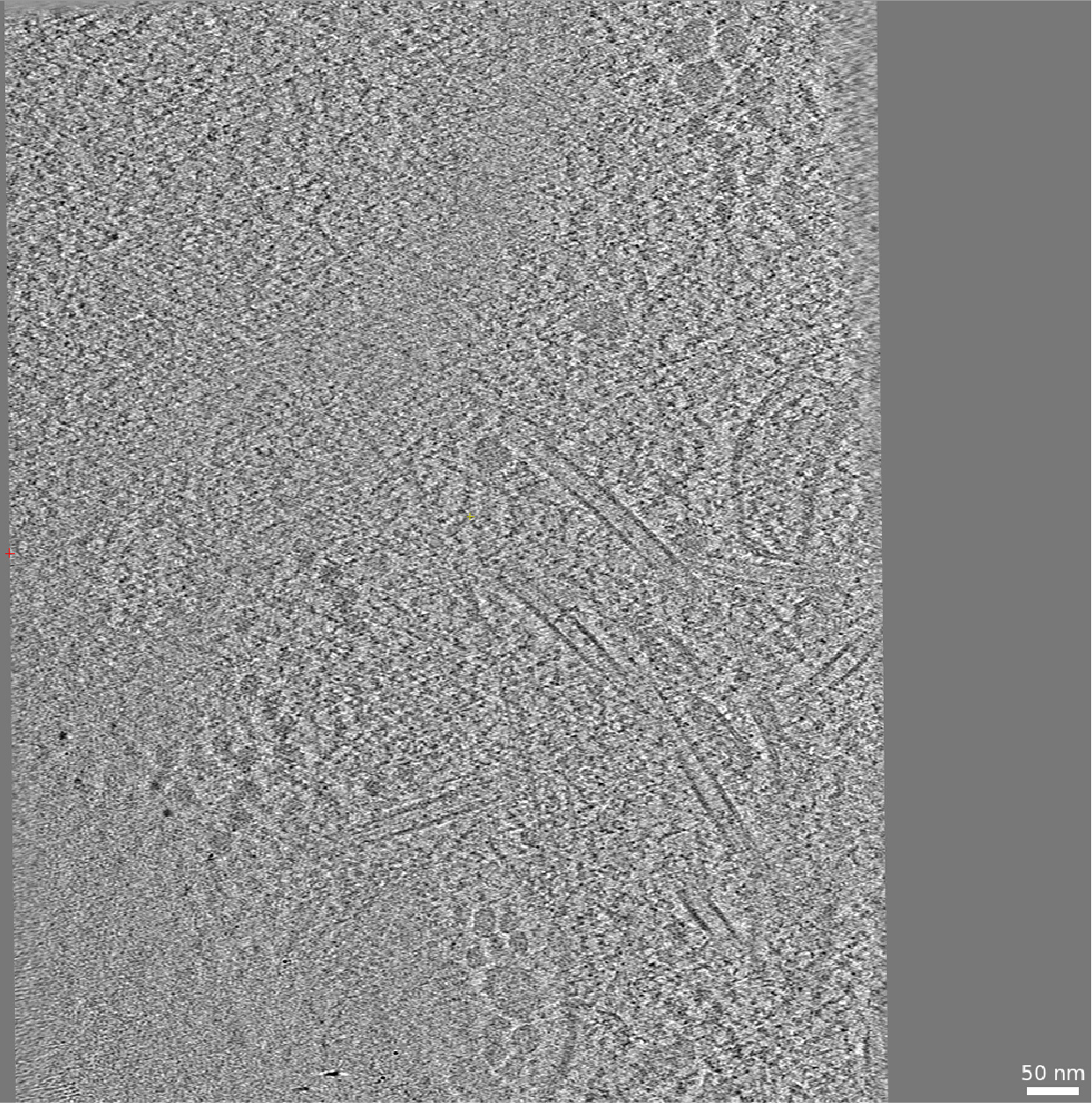

Supplement: Supplementary file 7 — Original images of the figure in high resolution. [file 41592_2026_3127_MOESM7_ESM.zip › Figure4/Fig4_CellsAdh_micropraph.png]

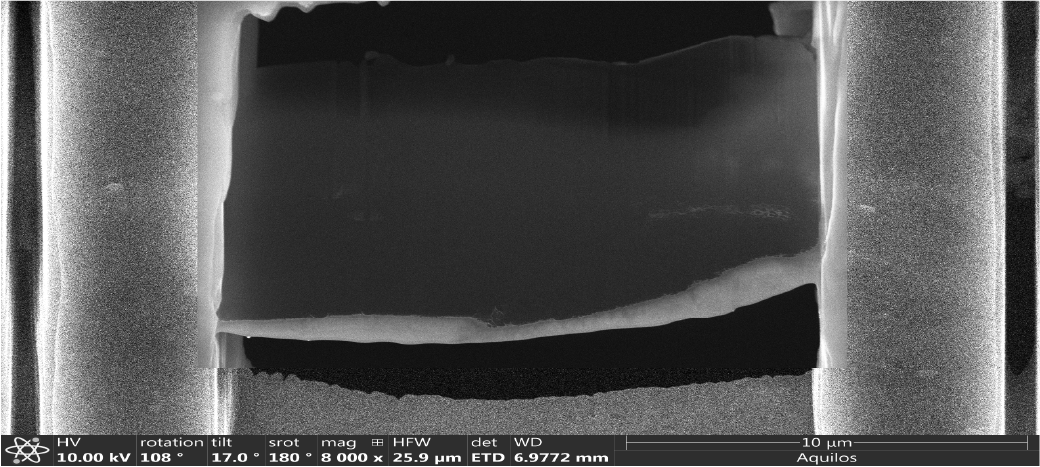

Supplement: Supplementary file 7 — Original images of the figure in high resolution. [file 41592_2026_3127_MOESM7_ESM.zip › Figure4/Fig4_CellsAdh_lamella1.png]

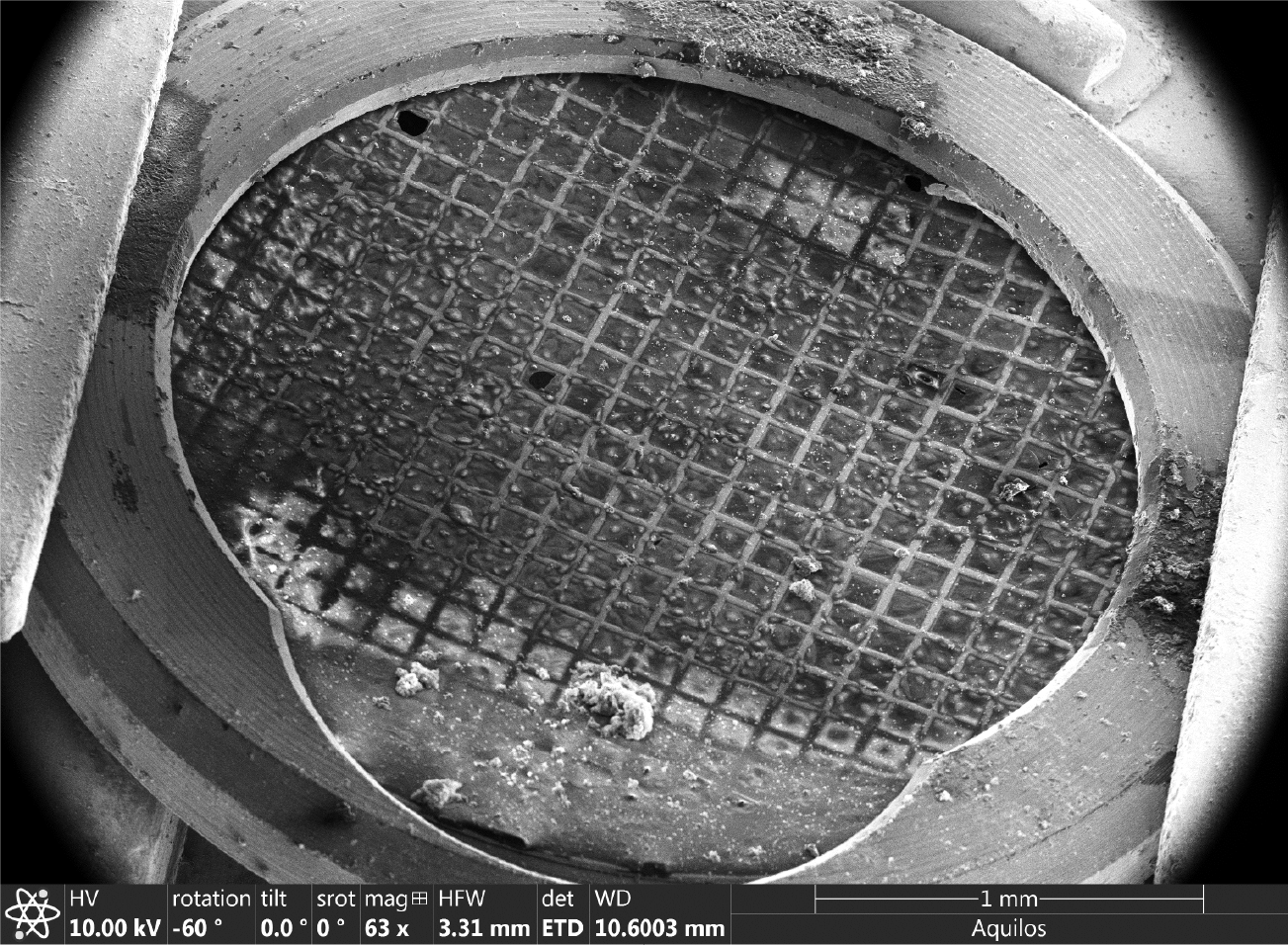

Supplement: Supplementary file 7 — Original images of the figure in high resolution. [file 41592_2026_3127_MOESM7_ESM.zip › Figure4/Fig4_CellsAdh_cryoETgrid.png]

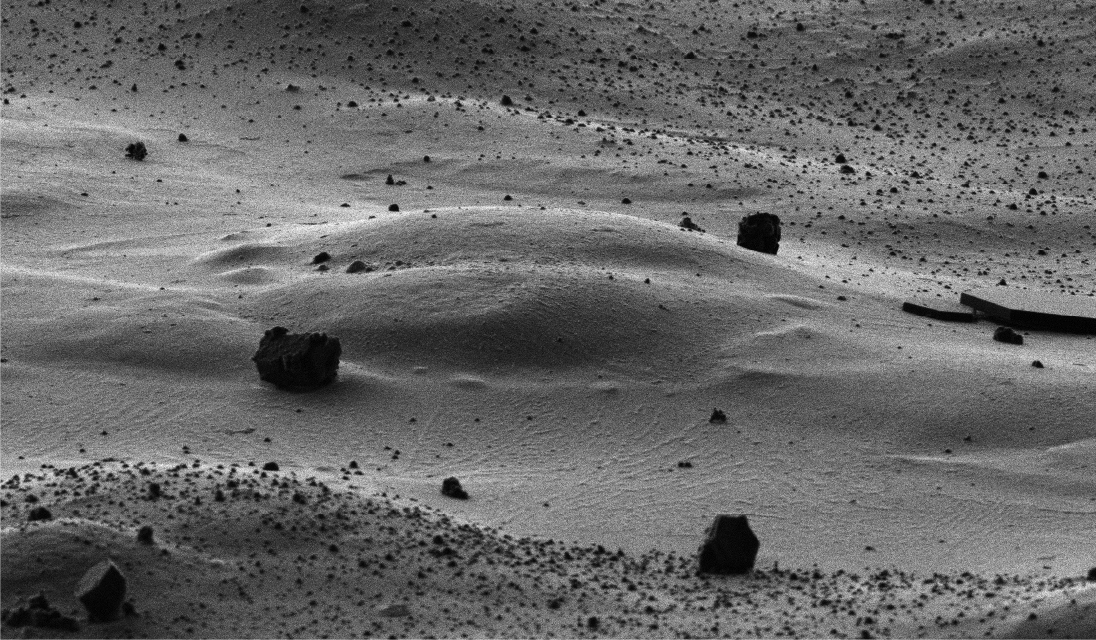

Supplement: Supplementary file 7 — Original images of the figure in high resolution. [file 41592_2026_3127_MOESM7_ESM.zip › Figure4/Fig4_CellsAdh_cellOnGrid.png]

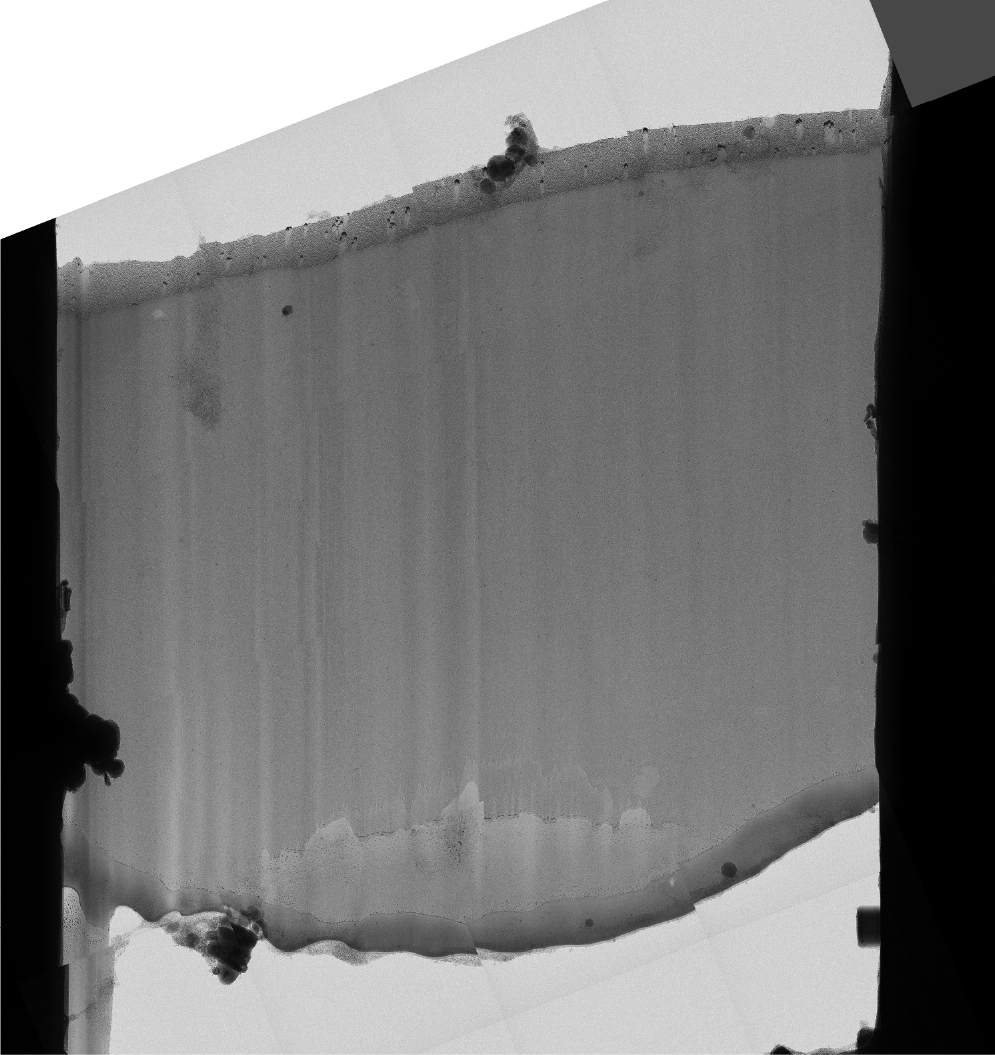

Supplement: Supplementary file 8 — Original images of the figure in high resolution. [file 41592_2026_3127_MOESM8_ESM.zip › Figure5/Fig5_iceQ_lamellaEG2.png]

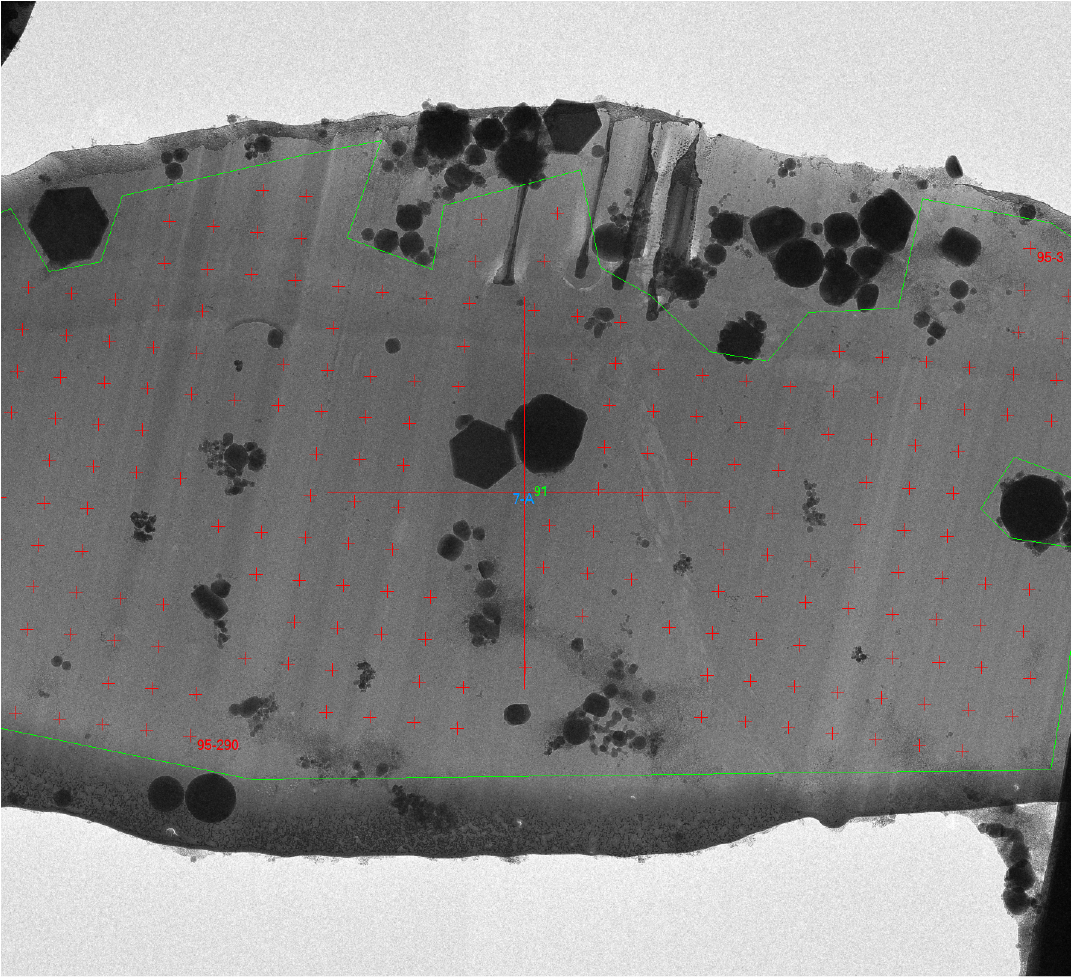

Supplement: Supplementary file 8 — Original images of the figure in high resolution. [file 41592_2026_3127_MOESM8_ESM.zip › Figure5/Fig5_iceQ_lamellaEG2acquisition.png]

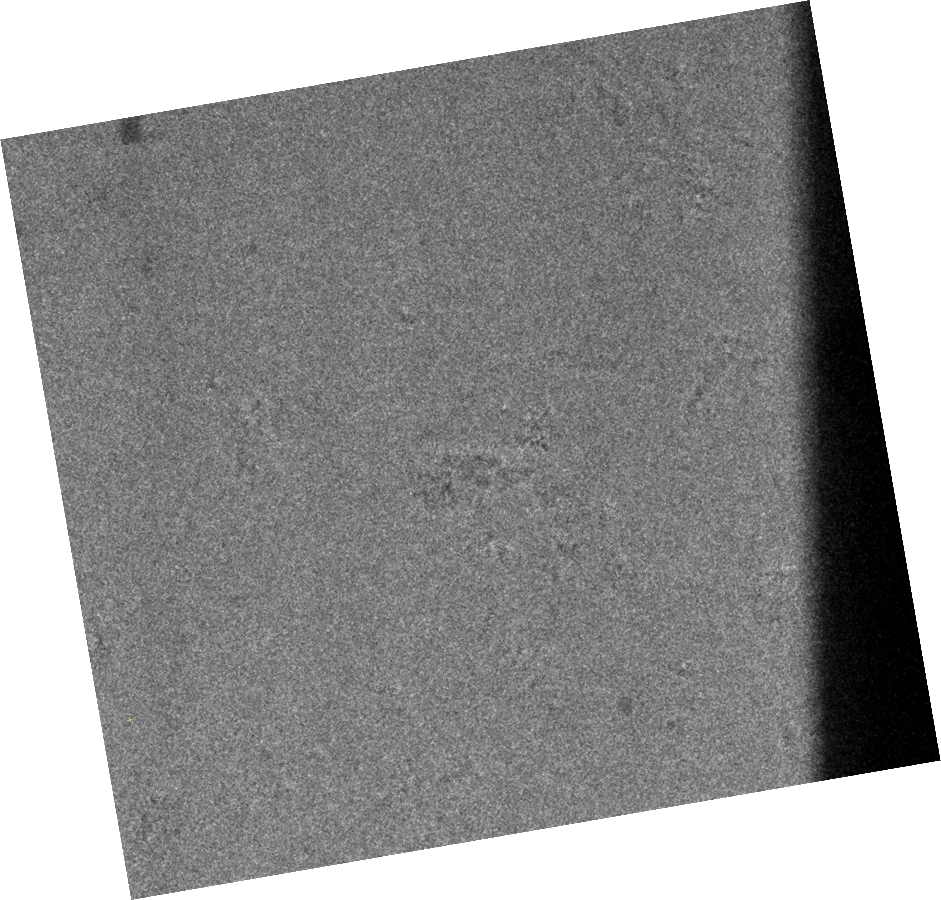

Supplement: Supplementary file 8 — Original images of the figure in high resolution. [file 41592_2026_3127_MOESM8_ESM.zip › Figure5/Fig5_iceQ_micropragh1.png]

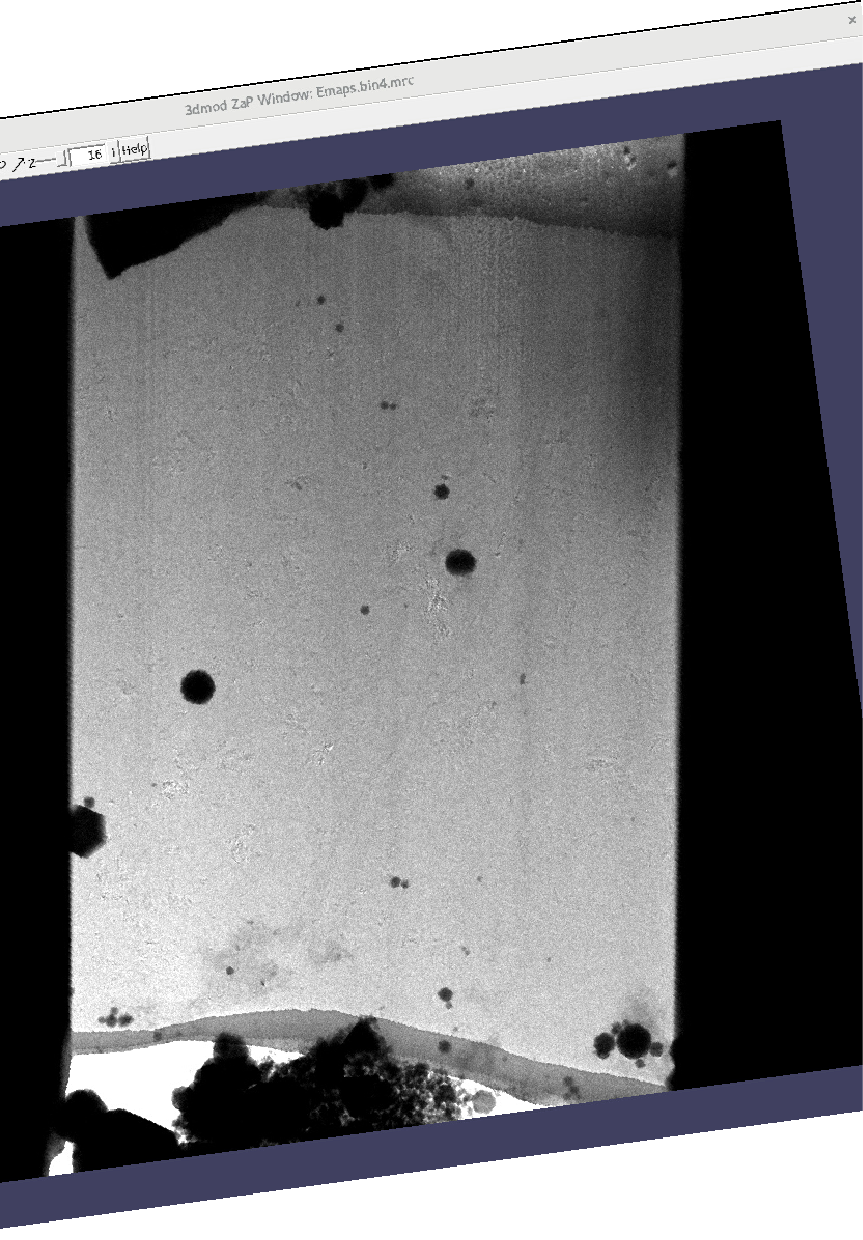

Supplement: Supplementary file 8 — Original images of the figure in high resolution. [file 41592_2026_3127_MOESM8_ESM.zip › Figure5/Fig5_iceQ_lamellaGP2.png]

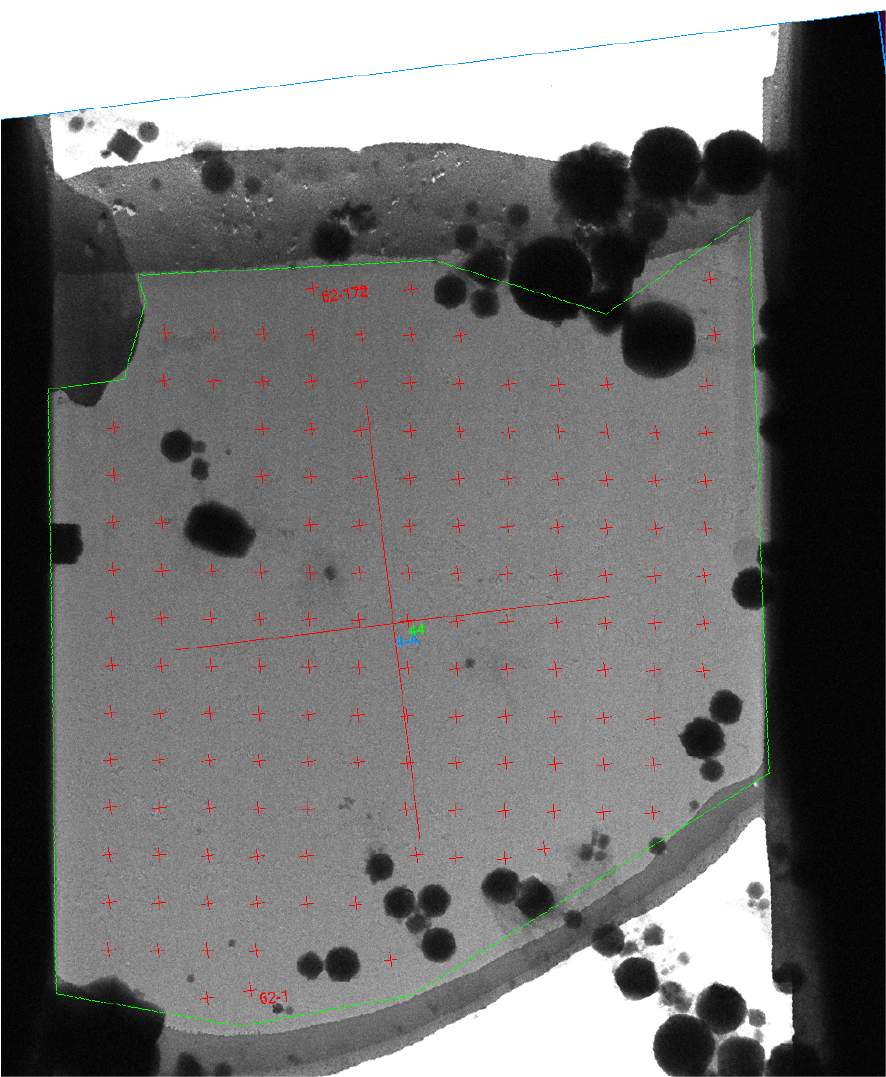

Supplement: Supplementary file 8 — Original images of the figure in high resolution. [file 41592_2026_3127_MOESM8_ESM.zip › Figure5/Fig5_iceQ_lamellaGP2acquisition.png]

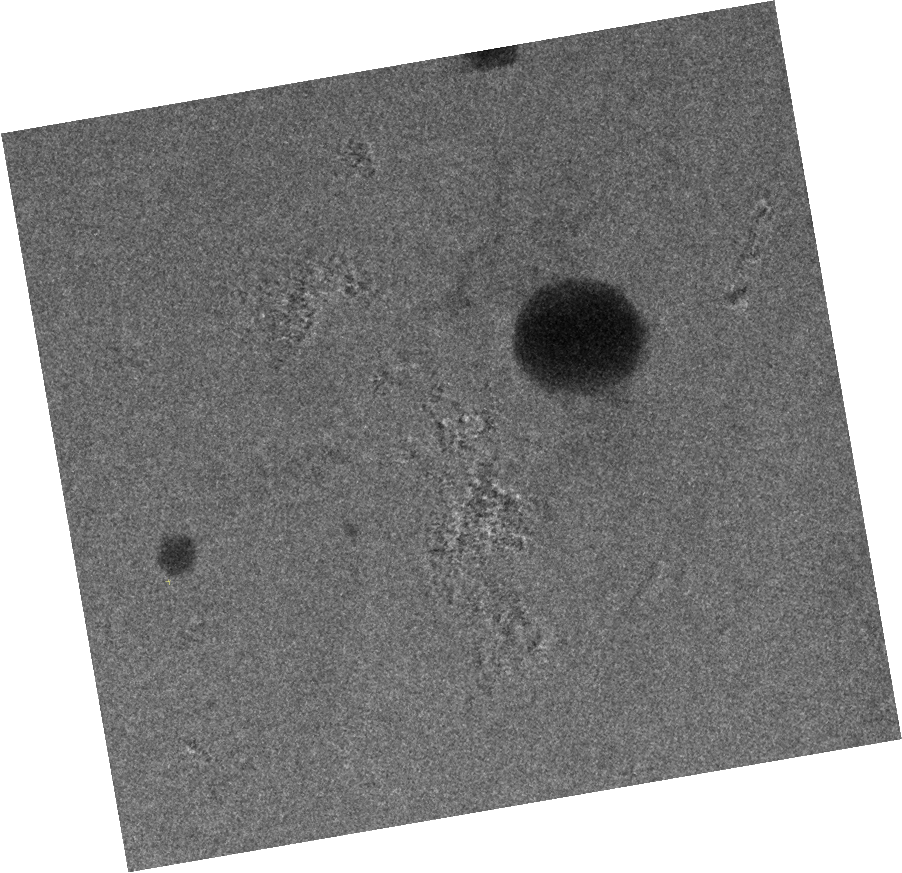

Supplement: Supplementary file 8 — Original images of the figure in high resolution. [file 41592_2026_3127_MOESM8_ESM.zip › Figure5/Fig5_iceQ_micrograph2.png]

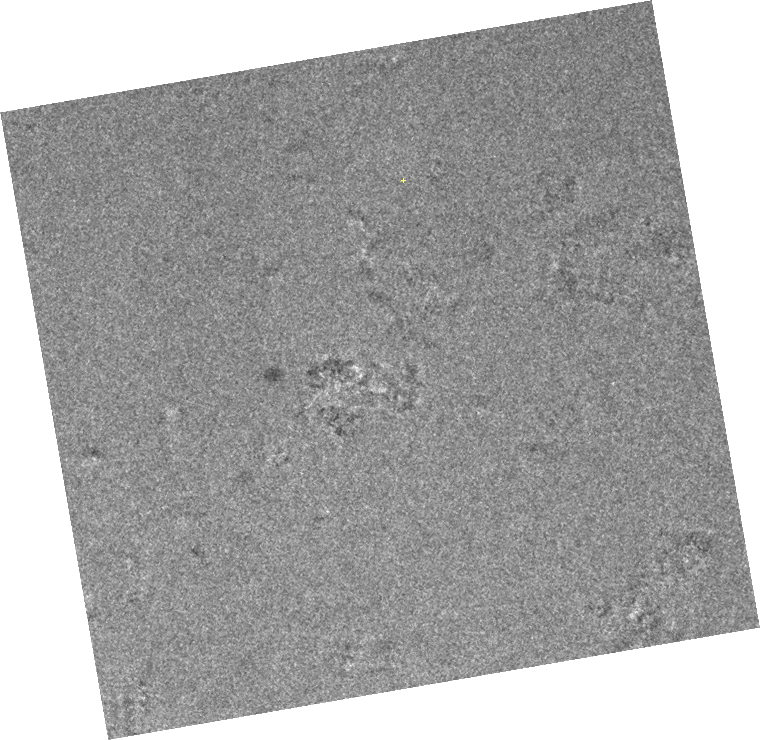

Supplement: Supplementary file 8 — Original images of the figure in high resolution. [file 41592_2026_3127_MOESM8_ESM.zip › Figure5/Fig5_iceQ_micrograph3.png]

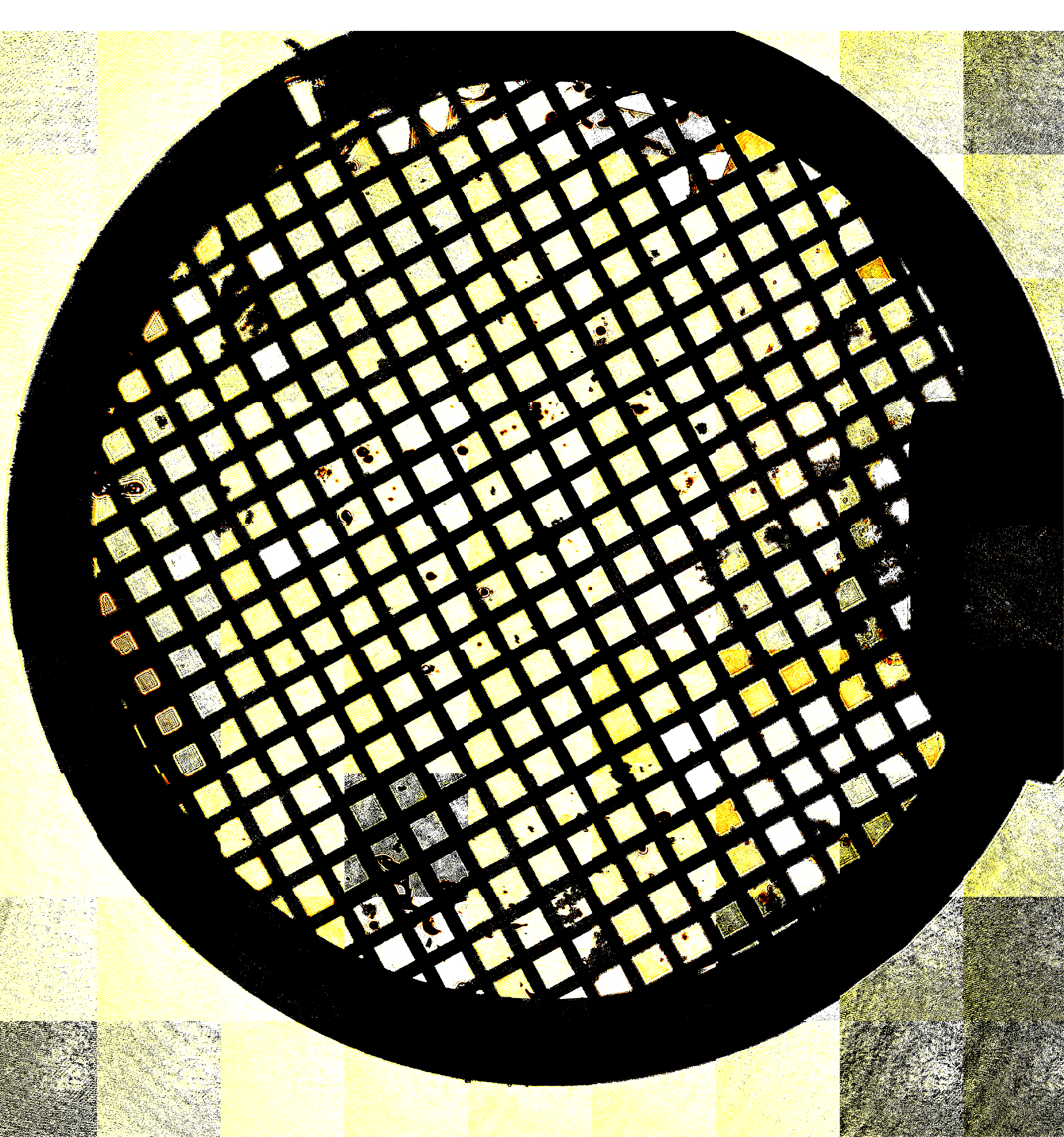

Supplement: Supplementary file 9 — Original images of the figure in high resolution. [file 41592_2026_3127_MOESM9_ESM.zip › ExtendedFigure1/SupFig1-EGCgridSorting_EGCcells2.png]

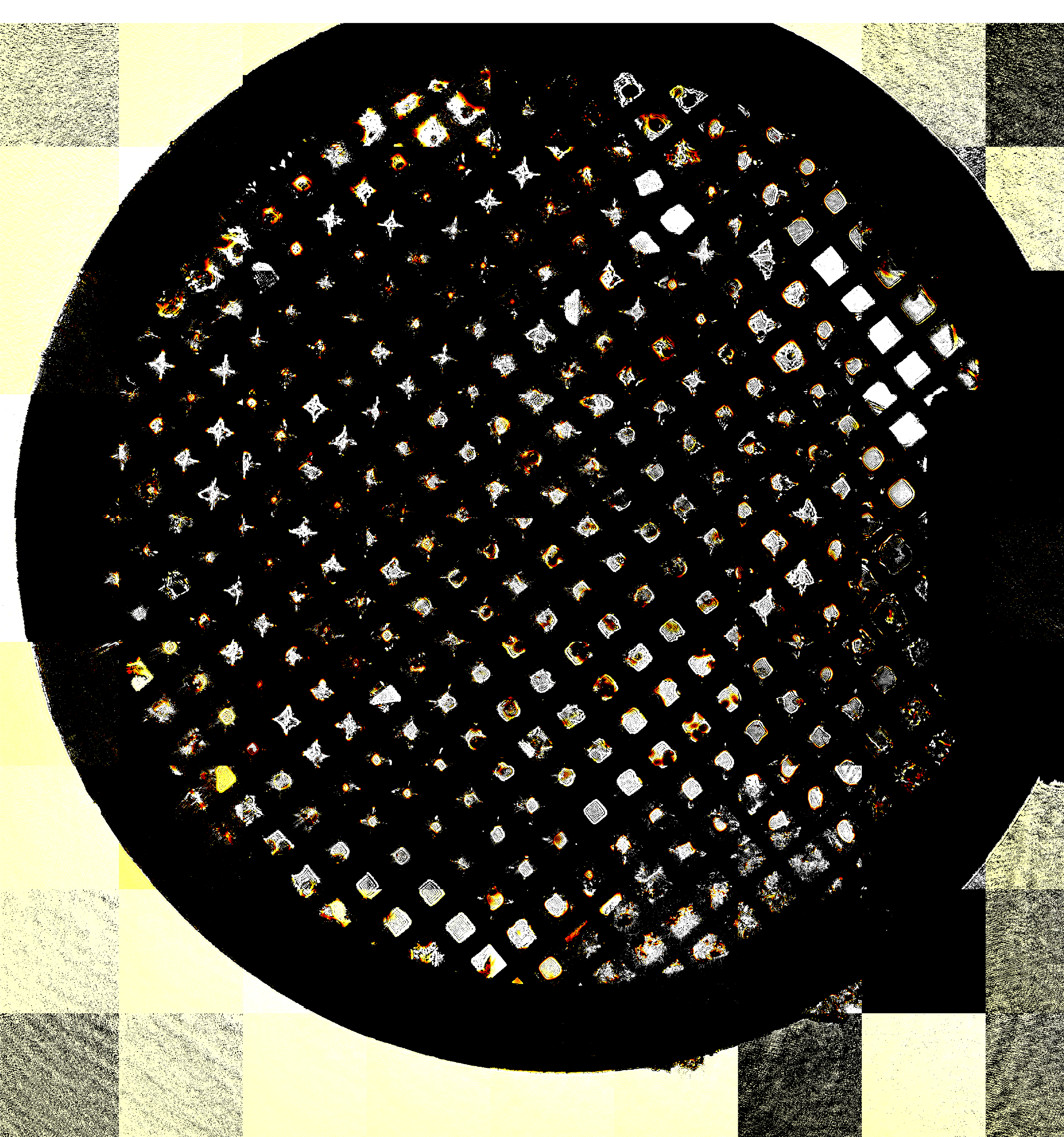

Supplement: Supplementary file 9 — Original images of the figure in high resolution. [file 41592_2026_3127_MOESM9_ESM.zip › ExtendedFigure1/SupFig1-EGCgridSorting_EGCcells3.png]

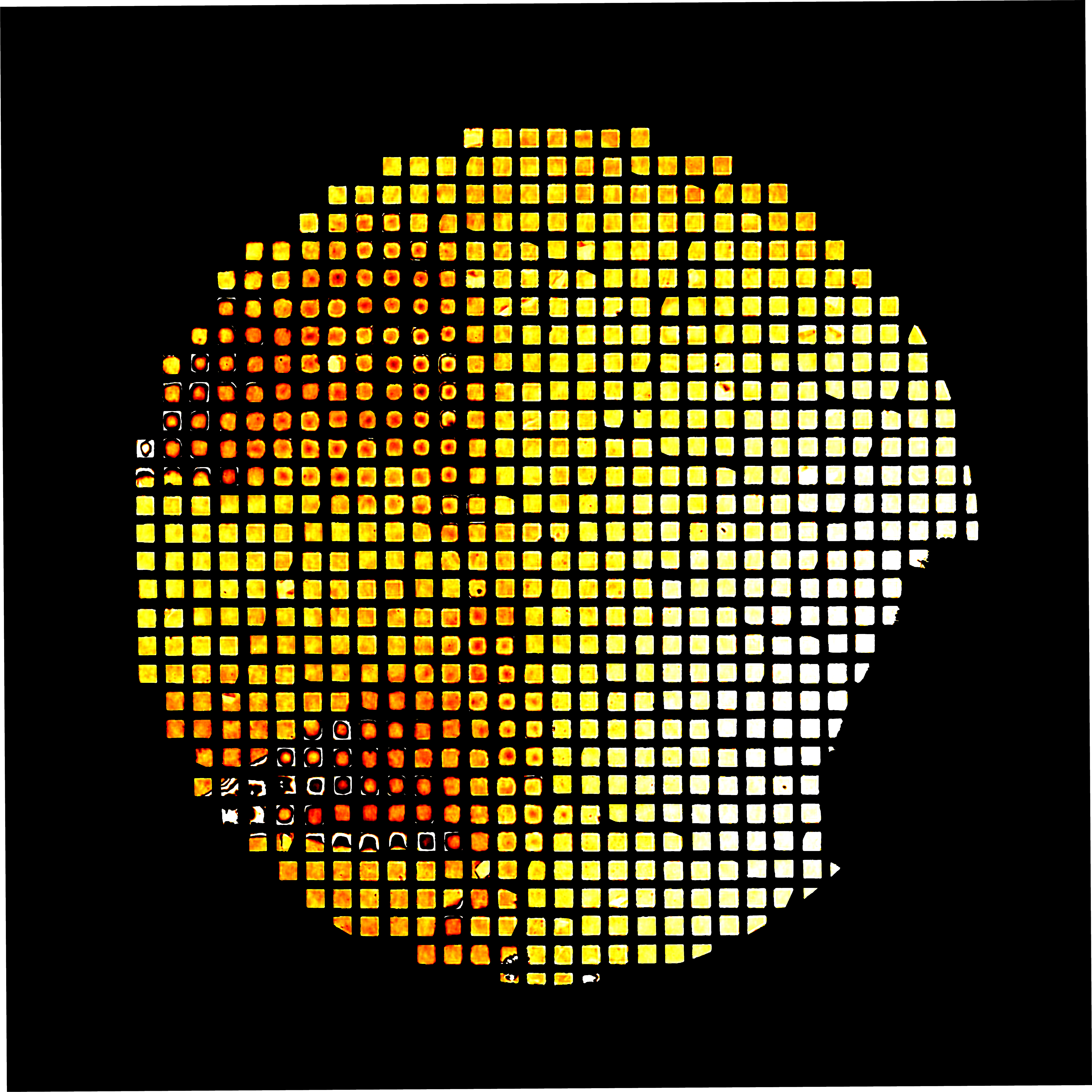

Supplement: Supplementary file 9 — Original images of the figure in high resolution. [file 41592_2026_3127_MOESM9_ESM.zip › ExtendedFigure1/SupFig1-EGCgridSorting_EGCgrid1.png]

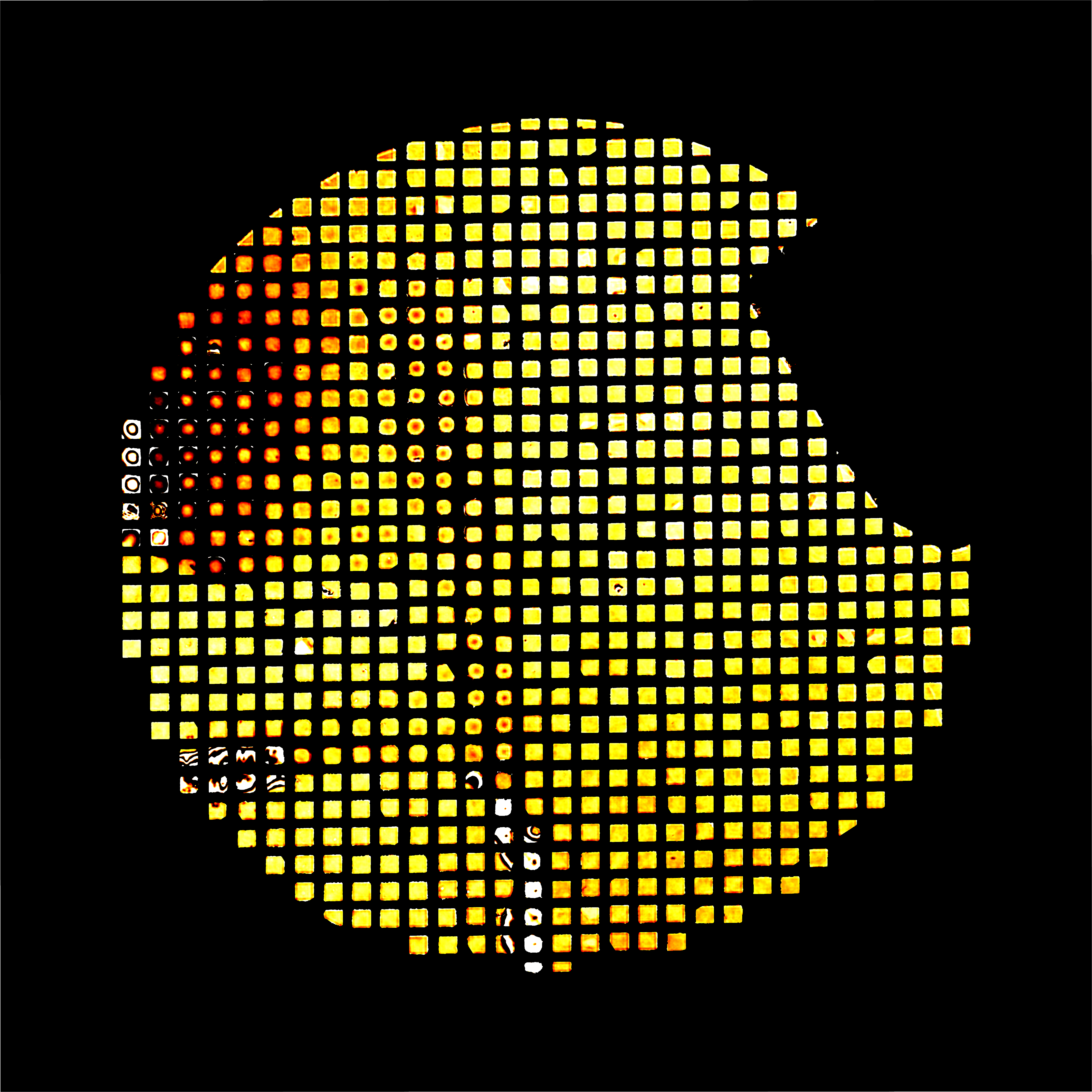

Supplement: Supplementary file 9 — Original images of the figure in high resolution. [file 41592_2026_3127_MOESM9_ESM.zip › ExtendedFigure1/SupFig1-EGCgridSorting_EGCgrid3.png]

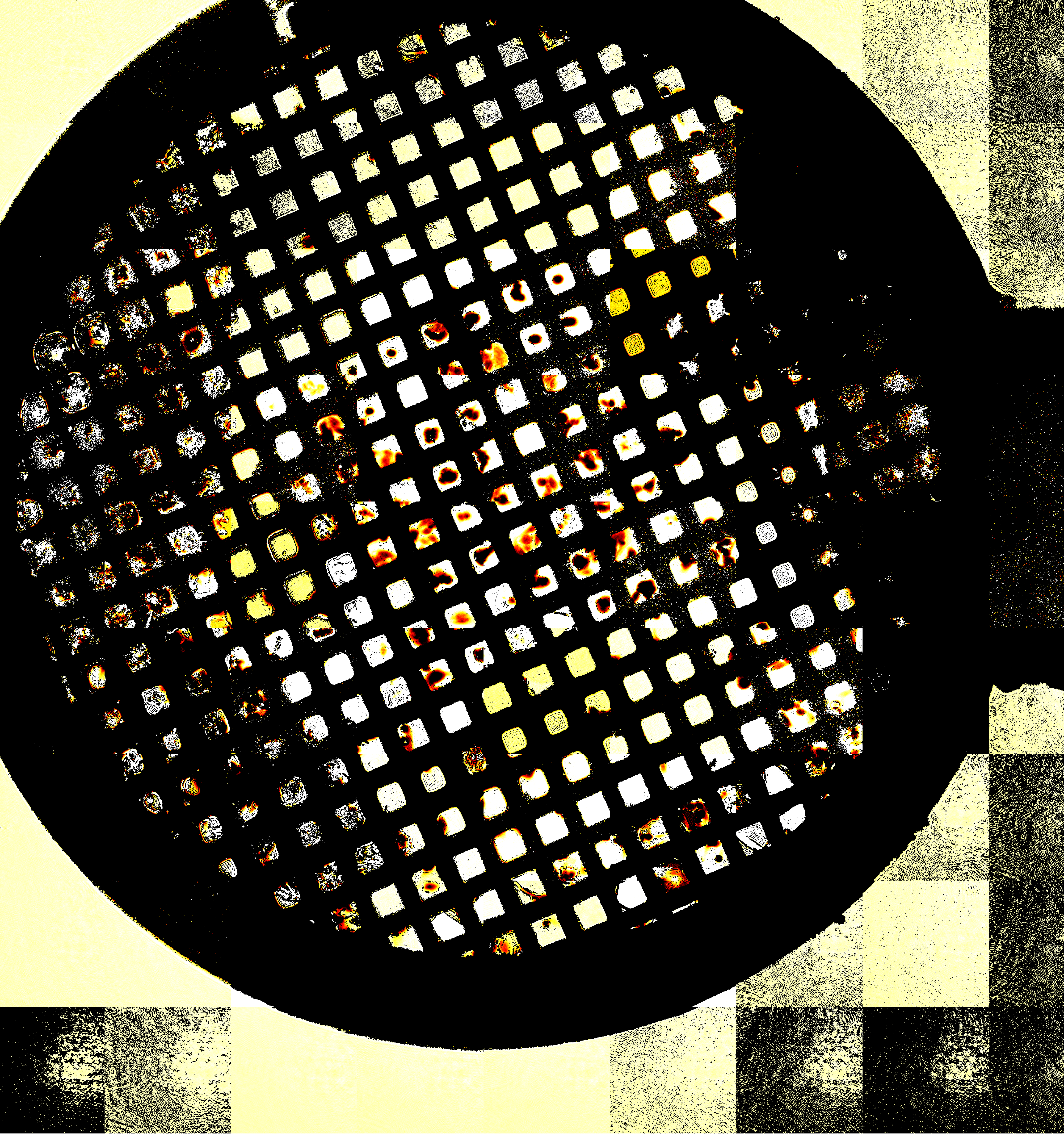

Supplement: Supplementary file 9 — Original images of the figure in high resolution. [file 41592_2026_3127_MOESM9_ESM.zip › ExtendedFigure1/SupFig1-EGCgridSorting_EGCcells1.png]

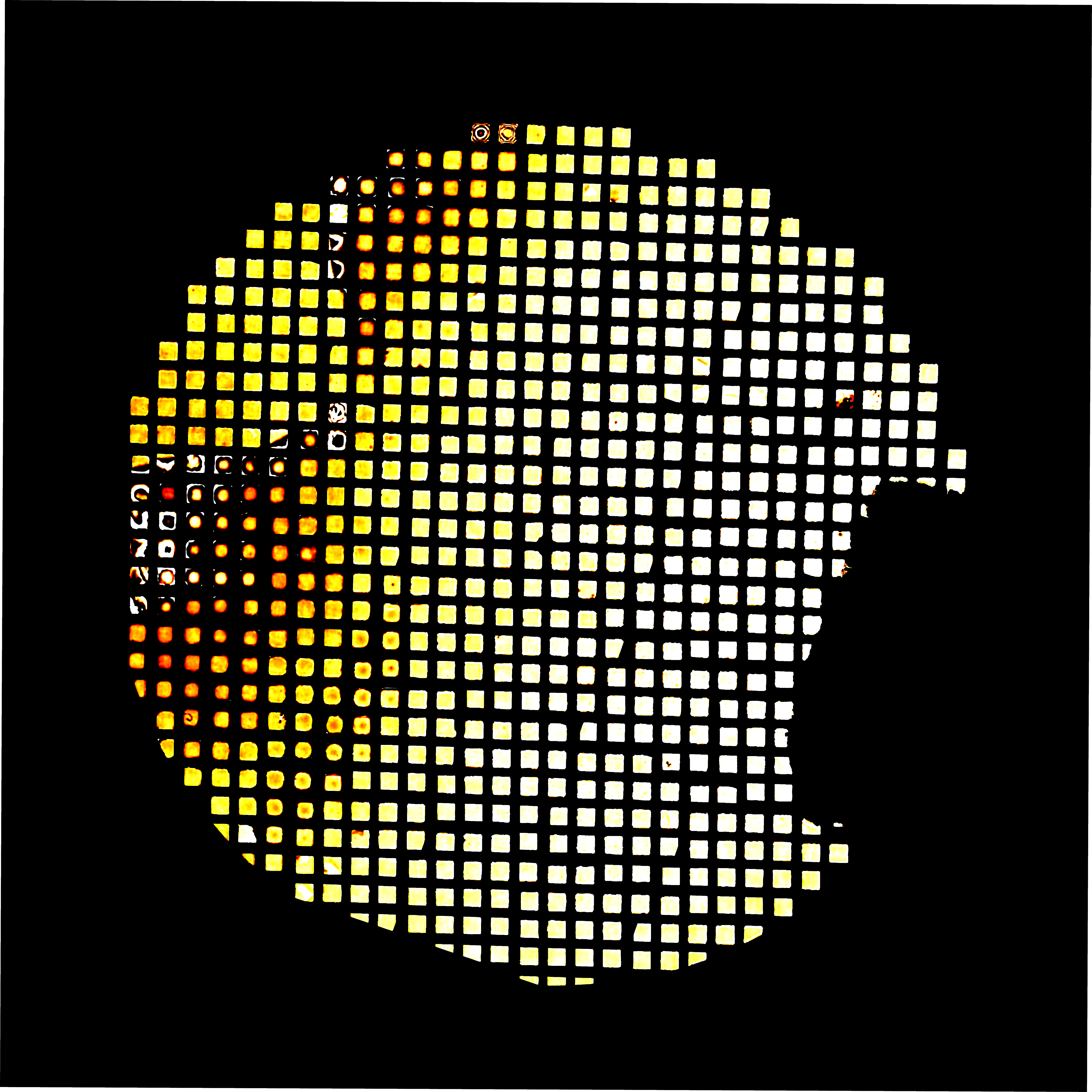

Supplement: Supplementary file 9 — Original images of the figure in high resolution. [file 41592_2026_3127_MOESM9_ESM.zip › ExtendedFigure1/SupFig1-EGCgridSorting_EGCgrid2.png]

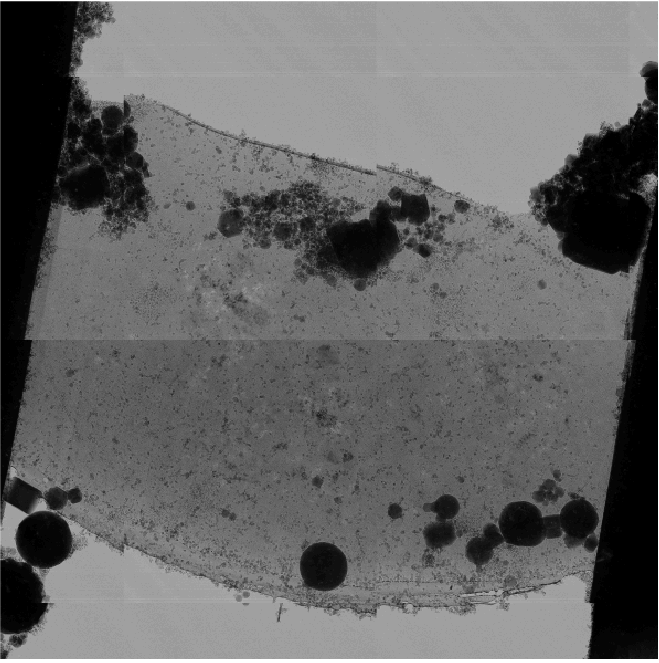

Supplement: Supplementary file 10 — Original images of the figure in high resolution. [file 41592_2026_3127_MOESM10_ESM.zip › ExtendedFigure8/SupFig8-ABsum159_GP2lamella2.png]

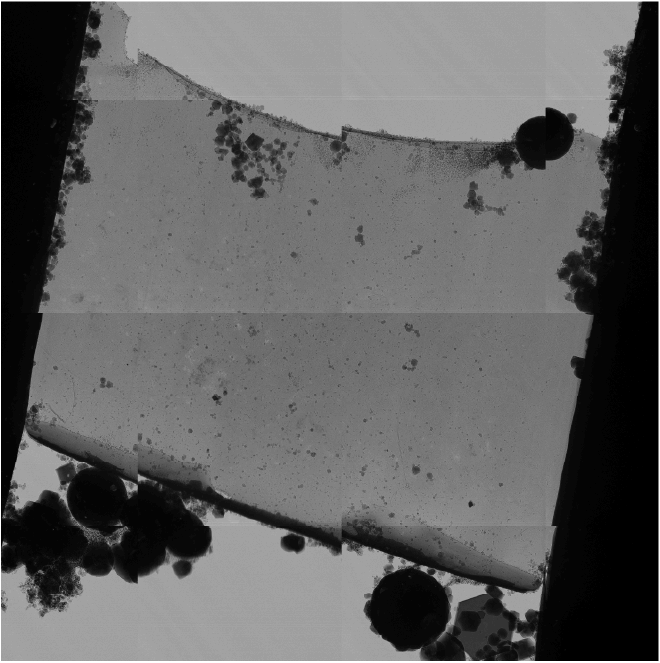

Supplement: Supplementary file 10 — Original images of the figure in high resolution. [file 41592_2026_3127_MOESM10_ESM.zip › ExtendedFigure8/SupFig8-ABsum159_GP2lamella3.png]

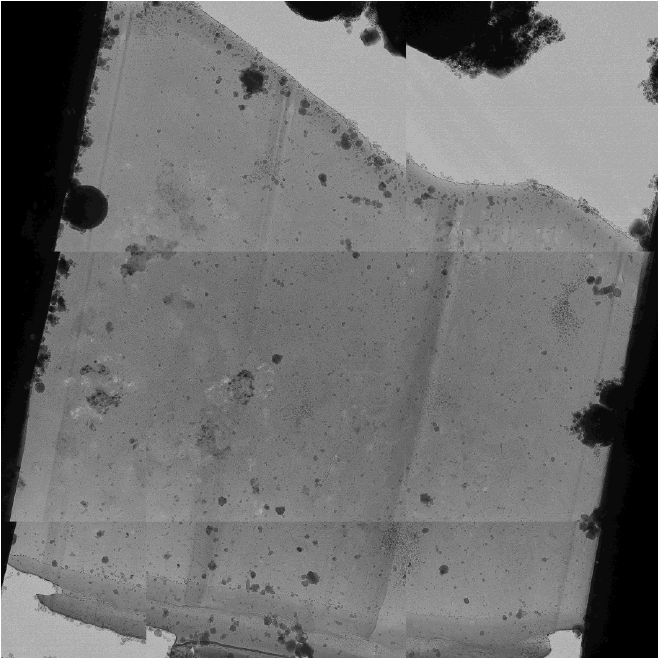

Supplement: Supplementary file 10 — Original images of the figure in high resolution. [file 41592_2026_3127_MOESM10_ESM.zip › ExtendedFigure8/SupFig8-ABsum159_GP2lamella1.png]

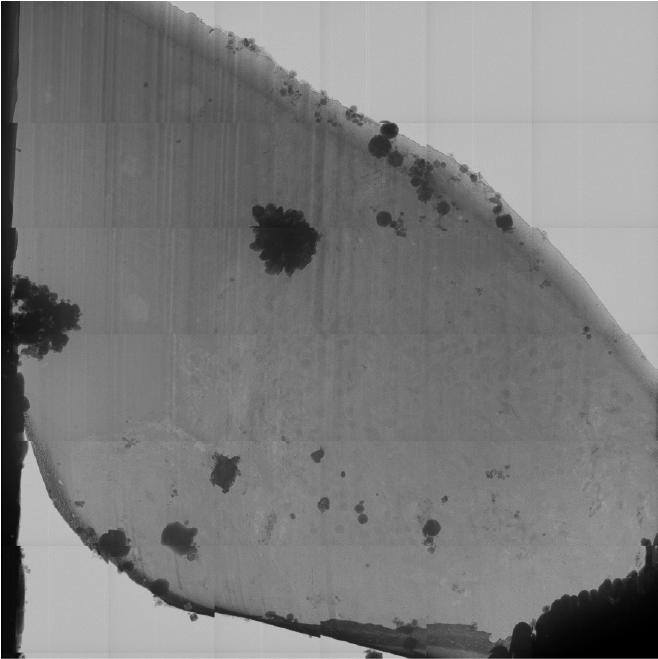

Supplement: Supplementary file 10 — Original images of the figure in high resolution. [file 41592_2026_3127_MOESM10_ESM.zip › ExtendedFigure8/SupFig8-ABsum159_EG2lamella1.png]

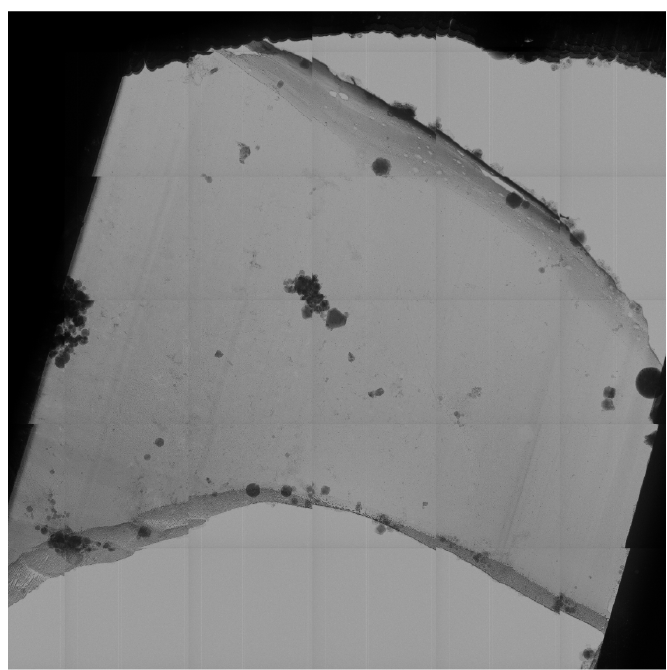

Supplement: Supplementary file 10 — Original images of the figure in high resolution. [file 41592_2026_3127_MOESM10_ESM.zip › ExtendedFigure8/SupFig8-ABsum159_EG2lamella2.png]

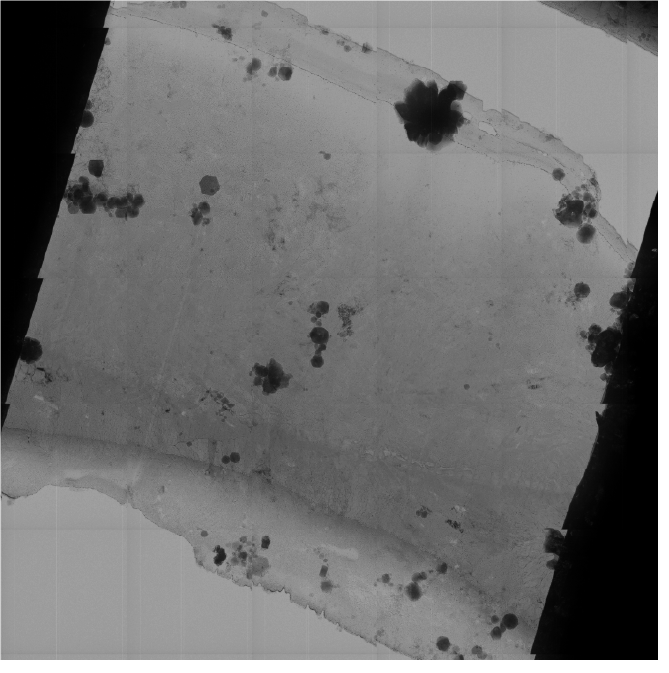

Supplement: Supplementary file 10 — Original images of the figure in high resolution. [file 41592_2026_3127_MOESM10_ESM.zip › ExtendedFigure8/SupFig8-ABsum159_EG2lamella3.png]
