# Supplementary material for: EasyGrid: a versatile platform for automated cryo-EM sample preparation and quality control
Source: Nat Methods. 2026 Jun 23;23(7):1359–67. doi: 10.1038/s41592-026-03127-5 (PMC13345917; doi:10.1038/s41592-026-03127-5)
Supplement: Supplementary file 3 — Peer Review File [file 41592_2026_3127_MOESM3_ESM.pdf]

nature portfolio

Peer Review File

EasyGrid: a versatile platform for automated cryo-EM sample preparation and quality control

Corresponding Author: Mr Gergely Papp

This file contains all editorial decision letters in order by version, followed by all author rebuttals in order by version.

A version of this paper was originally rejected for publication by Nature Methods, however that decision was reconsidered after appeal by the authors.

Version 1:

Decision Letter:

2nd Apr 2024

Dear Dr. Papp,

Your Article entitled "EasyGrid: a versatile platform for automated cryo-EM sample preparation and quality control" has now been seen by 3 reviewers, whose comments are attached. While they find your work of potential interest, they have raised serious concerns which in our view are sufficiently important that they preclude publication of the work in Nature Methods, at least in its present form.

As you will see, the reviewers raise concerns about a lack of compelling demonstrations showing the approach would be useful for more challenging samples, as well as a need to include data to demonstrate good general performance across samples and grid types. Characterizing AWI and ice thickness across diverse samples will also be important.

Should further experimental data allow you to fully address these criticisms we would be willing to look at a revised manuscript (unless, of course, something similar has by then been accepted at Nature Methods or appeared elsewhere). This includes submission or publication of a portion of this work somewhere else. We hope you understand that until we have read the revised paper in its entirety we cannot promise that it will be sent back for peer-review.

If you are interested in revising this manuscript for submission to Nature Methods in the future, please contact me to discuss your appeal before making any revisions. Otherwise, we hope that you find the reviewers' comments helpful when preparing your paper for submission elsewhere.

Sincerely,
Arunima

Arunima Singh, Ph.D.
Senior Editor
Nature Methods

Reviewers' Comments:

Reviewer #1:
Remarks to the Author:
Olivier et al. describe an exciting advance in cryo-EM/cryo-ET specimen technology. In this work, they develop an automated system called EasyGrid for cryo-EM sample preparation and quality control. EasyGrid facilitates the sample screening process and enables the mapping of ice thickness across the grid. With the EasyGrid, fine structures of apoferritin, yeast ribosomes and KR2 rhodopsin have been determined at about 2 Å resolution. They further show that large cells could exhibit better vitrification quality when prepared with EasyGrid compared to plunge-freezing. This study would appeal to researchers in the fields of cryo-EM/cryo-ET. The followings are my comments.
1. The interaction of proteins with the air-water interface is widely appreciated as a significant challenge that currently impedes

successful cryo-EM reconstruction and achievable resolution. From the 2D classification results in Figs. S3, S4 and S5, the orientations of particles are rich. It will be helpful to show the spatial distributions of protein particles in vitreous ice layer by cryo-ET to investigate whether the EasyGrid could alleviate the air-water interface effect.
2. An additional case with protein that is susceptible to the air-water interface should be tested using EasyGrid, because the structures of apoferritin, yeast ribosomes and KR2 rhodopsin are relatively robust. This will strengthen the significance of EasyGrid.
3. Is the EasyGrid system compatible with the grid covered with a continuous supporting film (e.g. amorphous carbon film, graphene)? It will be helpful to show the uniformity of ice thickness on such grid.
4. Can the authors comment on the reproducibility of the methodology? How many times did they repeat the experiments to get similarly thin ice sample? The statistics of ice thickness should be provided.
5. Can the authors provide a movie showing the liquid spreading process on the grid when using the pressure wave?
6. How fast is the cooling rate of jet-vitrification in EasyGrid system to ensure the ice quality in cells?
7. The authors mentioned “By contrast, we did not observe concentration differences between grids prepared with EasyGrid or with the Vitrobot using the selfsame sample solution”. The control results of Vitrobot should be provided.
8. What’s the meaning of “PCR-format” in the section of “Dual-nozzle picoliter drop dispenser”?

Reviewer #2:
Remarks to the Author:
The paper described the development of EasyGrid, fro better cryo-EM sample preparation both fo single particles (SPA) and tomography (ET). The instrument combines in-line plasma treatment to increase hydrophobicity of grids, dispensing of the sample in pL volumes, pressure-waves based spreading of the sample under humidity, followed by ethane-jets for vitrification. This is also followed byimagign with light interferometry to allow judging for sample thickness. The utility o the device is demonstrated with three samples (pretty standard choices) but also for cryo-ET vitrifying cells.

The technology is a leap forward compared to previous attempts, combining various rather ingenious solutions that have been described by others for cryo-EM or other applications, to put together an excellent process. The development is Cleary significant fo 5th field, but also in my opinion original. combining so many engineering solution in a way that is tailored for a problem at hand, is really original and inspiring.

The presentation is very good; the method is explained clearly, in logical steps and is very easy to read and understand.

The authors are careful with their conclusions, which are valid. While they are being honest that "to approach samples that are currently difficult to prepare for SPA, we will upgrade EasyGrid to perform automated screens of biochemical parameters" I need to add that demonstrating that for one or two such samples, would had been really exciting. While such data are not necessarily essential for publication, if the authors could provide such a case, where Easygrid made a project that was not possible before possible, it would be very convincing for future readers.

The references and presentation are excellent. The phrase "in-line plasma treatment" in the abstract needs to be clear it is referring to the grid surface.

In Figure 1A the green line for "zooming" is not working simply because the two renderings are not identical. Why not use identical rendering with same components, colours, so as the zoom is to an identical 3d object, even if from a a different angle? To show that the green box and the green dotted lines are not needed. The bright red and the green plate and carousels can be used as reference if present in the zoom-out. Also, the top row of (a) shoudl be followed by current (b) and the zoom-out and zoom-in shoudl be (c) and (d) in my view.

In Figure 2 the red line around parts of plane c and d is not working visually and I do not think it is needed, it is rather clear that SEM and TEM are not parts of the devise.

Reviewer #3:
Remarks to the Author:
Key results
In this work, the authors present a modular sample preparation device called EasyGrid, whose main novel aspect is its pressure-wave-based droplet spreader. The second novel aspect of this work is the use of digital holographic microscopy to provide ice thickness information on grids post-freezing. As evidence for the applicability of EasyGrid, the authors show high resolution (~2 Å) reconstructions of several single particle samples, mapping of ice thicknesses on frozen grids with the EGC module, and a significant reduction in crystalline ice through jet freezing of cellular samples.

Significance
Cryo-TEM imaging and screening of grids is an expensive endeavor. The EGC module promises to be a significant advancement towards screening grids ahead of cryo-TEM imaging. If the authors can develop the EGC module to be consistent across different grid types (or even just for carbon and gold foil grids) and to provide reliable ice thickness information, this will be a very significant improvement to the way grids are screened for cryo-EM.

Good sample preparation for in situ tomography imaging is one of the limiting steps in the cryo-tomography workflow. Jet-

freezing provides a way to rapidly cool thicker samples while producing vitreous ice.

Pressure-wave-based droplet spreading is a novel way of spreading the sample on a grid. In my opinion additional quantification is required to show the utility of the method, including how much of the grid has useful squares, how reproducible the method is, and what the resulting ice thicknesses are, especially for the SPA experiments.

The work presented here falls on the same lines of recent work including that of Ravelli et al (2020) for the Vitrojet – a jet freezing device with high-magnification light imaging of the grid to determine, to some extent, ice thickness; and the work of Koning et al (2022) developed with Linkam, which uses suction and high-magnification light imaging to monitor the progress of sample thinning. Both these recent works also automate the sample preparation process, like EasyGrid. To my mind, the two novel aspects shown in this work (pressure-wave-based droplet spreading and DHM mapping of ice thickness) need to be better quantified to justify publication in Nature Methods. The authors admit in lines 242-248 that the EGC module for mapping ice thicknesses needs to be more generalizable to deal with different grid types and more accurately measuring ice thicknesses, and in this current version only “provides grid atlases enabling sample screening”, which is not so different from existing work. Once said improvements are implemented, however, DHM for grid screening itself will indeed be a significant advancement for cryo-EM grid screening, especially if it has a convincing cost-benefit analysis vs TEM screening.

Data, methodology, and analysis
The section on ice quality assessment on lamellae was well done. Quantification was done to show ice quality, and the analysis was thorough and thoughtful, and an automated assessment of ice quality was developed.

Major comments and questions
- Can the authors provide some statistics about the success rate of SPA grids prepared by the EasyGrid system? What proportion of grids prepared are good enough for TEM imaging? How reproducible are grids prepared under the same conditions? How much optimization needs to be done every time a new sample is used?
- Can the authors provide some statistics about the number of useful squares per SPA grid prepared with the pressure-wave-based sample spreading module? Some cryo-EM sample preparation devices (e.g. chameleon, Vitrojet) only produce 15~30 useful squares per grid, so multiple grids often must be prepared for a full Krios data collection.
- Can the authors provide data on the spread of ice thicknesses in foil holes produced by EasyGrid? Since producing thin ice is an essential goal for high resolution SPA, this data would be reassuring to scientists working with non-test samples.
- Please comment on whether the pressure wave affects the morphology of the cells on the grid.

Minor comments and questions
- I’m curious if ice thickness can be controlled by adjusting the pressure of the wave?
- Line 73: How much is the EGC module expected to cost? A comparison of the setup and imaging cost of the EGC / DHM module vs screening on a TEM is needed to justify the claim of it being “inexpensive”.
- Line 140: Can the EGC module be used with all types of grid boxes?
- Line 179: Could the structure of KR2 not be solved by conventional plunge freezing techniques?
- Line 267: What concentration of apoferritin was used?
- Line 270: Why was a pre-coating of apoferritin on the grids needed?
- Line 309: Why was a coating of Blue Dextran needed for ribosomes and rhodopsin? Why not for apoferritin? Did having BD affect contrast or ice crystallinity in the images?
- Line 312: What concentration of ribosomes was used?
- Line 864: What property of the sample determines the number of passages in front of the plasma treatment? Is there a generalizable rule, or should this be determined for each sample?

Clarity and context
Paper is written clearly and is easily understood. Figures are very well done.

** For Nature Portfolio general information and news for authors, see http://npg.nature.com/authors.

Version 2:

Decision Letter:

Our ref: NMETH-A54831B-Z

9th Dec 2025

Dear Dr. Papp,

Thank you for submitting your revised manuscript "EasyGrid: a versatile platform for automated cryo-EM sample preparation and quality control" (NMETH-A54831B-Z). It has now been seen by the original referees and their comments are below. The reviewers find that the paper has improved in revision, and therefore we'll be happy in principle to publish it in Nature Methods, pending minor revisions to satisfy the referees' final requests and to comply with our editorial and formatting guidelines.

We are now performing detailed checks on your paper and will send you a checklist detailing our editorial and formatting requirements within two weeks or so. Please do not upload the final materials and make any revisions until you receive this additional information from us.

TRANSPARENT PEER REVIEW
Nature Methods offers a transparent peer review option for new original research manuscripts submitted from 17th February 2021. We encourage increased transparency in peer review by publishing the reviewer comments, author rebuttal letters and editorial decision letters if the authors agree. Such peer review material is made available as a supplementary peer review file. **Please state in the cover letter 'I wish to participate in transparent peer review' if you want to opt in, or 'I do not wish to participate in transparent peer review' if you don't.** Failure to state your preference will result in delays in accepting your manuscript for publication.

Please note: we allow redactions to authors' rebuttal and reviewer comments in the interest of confidentiality. If you are concerned about the release of confidential data, please let us know specifically what information you would like to have removed. Please note that we cannot incorporate redactions for any other reasons. Reviewer names will be published in the peer review files if the reviewer signed the comments to authors, or if reviewers explicitly agree to release their name. For more information, please refer to our FAQ page.

ORCID
IMPORTANT: Non-corresponding authors do not have to link their ORCIDs but are encouraged to do so. Please note that it will not be possible to add/modify ORCIDs at proof. Thus, please let your co-authors know that if they wish to have their ORCID added to the paper they must follow the procedure described in the following link prior to acceptance:
https://www.springernature.com/gp/researchers/orcid/orcid-for-nature-research

Author names using non-Roman characters
Nature Portfolio journals can support presentation of author names using non-Roman characters in the HTML version of the article. If you wish to, please include author names in parentheses after the Roman-character spelling; see example online here. Currently supported scripts are: Arabic, Chinese, Cyrillic, Devanagari, Greek, Hebrew, Hangul, Japanese and Persian. You will be asked to verify the rendering is correct at proof stage.

Thank you again for your interest in Nature Methods. Please do not hesitate to contact me if you have any questions. We will be in touch again soon.

Sincerely,
Arunima

Arunima Singh, Ph.D.
Senior Editor
Nature Methods

Reviewer #1 (Remarks to the Author):

The work on automated cryo-EM sample preparation remain impressive, and the authors have improved the manuscript over the past 18 months. I recommend this work for publication after addressing the following issues.
1. Figure 2c and Figure 3b are confusing to me. Based on my understanding, Figure 2c is a grid atlas showing vitreous ice layers present in most grid squares, whereas Figure 3b only displays a limited linear region with ice layers. Why is this the case? Is this method only capable of preparing small-area ice layers? The authors should clarify this to facilitate reader comprehension.
2. In Figure 2c, there is only a black arrow mentioned, with no white arrow.
3. In the sentence of line 54-55, recent works on sample thickness should be appropriately cited including:
https://doi.org/10.1016/j.bpj.2019.12.017
https://www.nature.com/articles/s41592-022-01693-y
https://www.nature.com/articles/s41592-021-01130-6

Reviewer #2 (Remarks to the Author):

This is a significantly improved version of an already appealing paper that was describing EasyGrid. I was positive about the work then, and the authors have here address my comments well, notably including original structure determination experiments utilising their technology. I also see they addressed multiple other comments from the other referees, in a manner that appears competent to me.

I have no specific comments.

Reviewer #3 (Remarks to the Author):

In my original review, I requested better quantification of DHM ice thickness measurements and pressure wave droplet spreading to justify the utility of EasyGrid and its publication in Nature Methods. The authors have conducted an extensive and thorough characterization of both these aspects of their system. Their results and additional data that they have provided in response to other reviewer comments are convincing, and demonstrate the broad utility of the device for both SPA and cell freezing for cryoET/lamella preparation. I expect the ability to rapidly obtain absolute ice thickness measurements from multiple grids in the DHM module to significantly improve the current, slow and expensive method of screening grids in a cryoTEM. This device is a significant advance in the sample preparation field, and the highlights of this work, for me, are the authors' ingenuity, meticulousness in their data collection and analysis, and obvious skill in engineering.

Version 3:

Decision Letter:

12th May 2026

Dear Gregely,

I am pleased to inform you that your Article, "EasyGrid: a versatile platform for automated cryo-EM sample preparation and quality control", has now been accepted for publication in Nature Methods. The received and accepted dates will be January 11, 2024 and May 12, 2026. This note is intended to let you know what to expect from us over the next month or so, and to let you know where to address any further questions.

Over the next few weeks, your paper will be copyedited to ensure that it conforms to Nature Methods style. Once your paper is typeset, you will receive an email with a link to choose the appropriate publishing options for your paper and our Author Services team will be in touch regarding any additional information that may be required. It is extremely important that you let us know now whether you will be difficult to contact over the next month. If this is the case, we ask that you send us the contact information (email, phone and fax) of someone who will be able to check the proofs and deal with any last-minute problems.

After the grant of rights is completed, you will receive a link to your electronic proof via email with a request to make any corrections within 48 hours. If, when you receive your proof, you cannot meet this deadline, please inform us at rjsproduction@springernature.com immediately.

Authors may need to take specific actions to achieve compliance with funder and institutional open access mandates.
If your research is supported by a funder that requires immediate open access (e.g. according to Plan S principles or the NIH public access policy) then you should select the gold OA route, and we will direct you to the compliant route where possible. Because authors warrant under our subscription licensing terms that they haven't committed to licensing any version of their article under a licence inconsistent with the terms of our agreement – including the applicable embargo period – publication under the subscription model isn't suitable for authors whose funders require no embargo.

If you have any questions about our publishing options, costs, Open Access requirements, or our legal forms, please contact ASJournals@springernature.com

If you have posted a preprint on any preprint server, please ensure that the preprint details are updated with a publication reference, including the DOI and a URL to the published version of the article on the journal website.

You may wish to make your media relations office aware of your accepted publication, in case they consider it appropriate to organize some internal or external publicity. Once your paper has been scheduled you will receive an email confirming the publication details. This is normally 3-4 working days in advance of publication. If you need additional notice of the date and time of publication, please let the production team know when you receive the proof of your article to ensure there is sufficient time to coordinate. Further information on our embargo policies can be found here: https://www.nature.com/authors/policies/embargo.html

To assist our authors in disseminating their research to the broader community, our SharedIt initiative provides you with a unique shareable link that will allow anyone (with or without a subscription) to read the published article. Recipients of the link with a subscription will also be able to download and print the PDF.

As soon as your article is published, you will receive an automated email with your shareable link.

If you are active on Twitter/X or Bluesky, please e-mail me your and your coauthors' handles so that we may tag you when the paper is published.

You can now use a single sign-on for all your accounts, view the status of all your manuscript submissions and reviews, access usage statistics for your published articles and download a record of your refereeing activity for the Nature journals.

Please note that you and any of your coauthors will be able to order reprints and single copies of the issue containing your article through Nature Portfolio's reprint website, which is located at http://www.nature.com/reprints/author-reprints.html. If there are any questions about reprints please send an email to author-reprints@nature.com and someone will assist you.

Please feel free to contact me if you have questions about any of these points.

Best regards,
Arunima

Arunima Singh, Ph.D.
Senior Editor
Nature Methods

** Visit the Springer Nature Editorial and Publishing website at www.springernature.com/editorial-and-publishing-jobs for more information about our career opportunities. If you have any questions please click here.**

Open Access This Peer Review File is licensed under a Creative Commons Attribution 4.0 International License, which permits use, sharing, adaptation, distribution and reproduction in any medium or format, as long as you give appropriate credit to the original author(s) and the source, provide a link to the Creative Commons license, and indicate if changes were made. In cases where reviewers are anonymous, credit should be given to 'Anonymous Referee' and the source.
The images or other third party material in this Peer Review File are included in the article's Creative Commons license, unless indicated otherwise in a credit line to the material. If material is not included in the article's Creative Commons license and your intended use is not permitted by statutory regulation or exceeds the permitted use, you will need to obtain permission directly from the copyright holder.
To view a copy of this license, visit https://creativecommons.org/licenses/by/4.0/

Response to reviewers

Reviewer #1:
Remarks to the Author:
The work on automated cryo-EM sample preparation remain impressive, and the authors have improved the manuscript over the past 18 months. I recommend this work for publication after addressing the following issues.

We sincerely thank the reviewer for their supportive and positive feedback. We are also deeply grateful for their insightful comments throughout the review process, which have enabled us to present a more convincing and comprehensive paper. Below, we address each of the final recommendations in detail.

1. Figure 2c and Figure 3b are confusing to me. Based on my understanding, Figure 2c is a grid atlas showing vitreous ice layers present in most grid squares, whereas Figure 3b only displays a limited linear region with ice layers. Why is this the case? Is this method only capable of preparing small-area ice layers? The authors should clarify this to facilitate reader comprehension.

In Figure 2c, we demonstrate the capabilities of the EasyGrid control instrument and compare the thickness map obtained from the EasyGrid control (EGC) with the atlas generated using a Glacios Cryo-Electron Microscope. The sample grid shown was not further analyzed for ice layer thickness or spread area size. In Figure 3b, we illustrate the sequential steps of sample preparation using the EG instrument. The images provided are intended to visually represent the different stages of the preparation process, without any optimization for the specific sample. As noted throughout the manuscript, the behavior of samples on Cryo-EM grids can vary significantly depending on both the preparation parameters and the sample itself. Therefore, an optimization process for preparation parameters (as shown in Supplementary Figure SN16) must be applied to each new sample.
The captions of Figures 2 and 3 has been adjusted to comply with the reviewer's comments.

2. In Figure 2c, there is only a black arrow mentioned, with no white arrow.

Corrected. Thank you for pointing out this error.

3. In the sentence of line 54-55, recent works on sample thickness should be appropriately cited including:
https://doi.org/10.1016/j.bpj.2019.12.017
https://www.nature.com/articles/s41592-022-01693-y
https://www.nature.com/articles/s41592-021-01130-6

We appreciate the reviewer's suggestion to include these relevant works. The recommended references have been added to the manuscript, and the corresponding text has been revised for clarity (lines 55–56).

Reviewer #2:
Remarks to the Author:
This is a significantly improved version of an already appealing paper that was describing EasyGrid. I was positive about the work then, and the authors have here address my comments well, notably including original structure determination experiments utilising their technology. I also see they addressed multiple other comments from the other referees, in a manner that appears competent to me.

I have no specific comments.

We sincerely thank the reviewer for their positive feedback and for the time and effort dedicated to reviewing our work.

Reviewer #3:
Remarks to the Author:
In my original review, I requested better quantification of DHM ice thickness measurements and pressure wave droplet spreading to justify the utility of EasyGrid and its publication in Nature Methods. The authors have conducted an extensive and thorough characterization of both these aspects of their system. Their results and additional data that they have provided in response to other reviewer comments are convincing, and demonstrate the broad utility of

the device for both SPA and cell freezing for cryoET/lamella preparation. I expect the ability to rapidly obtain absolute ice thickness measurements from multiple grids in the DHM module to significantly improve the current, slow and expensive method of screening grids in a cryoTEM. This device is a significant advance in the sample preparation field, and the highlights of this work, for me, are the authors' ingenuity, meticulousness in their data collection and analysis, and obvious skill in engineering.

We are grateful to the reviewer for their relevant comments and suggestions, which have enabled us to refine our system and enhance the overall quality of our work.
